# The Influence of Varying Fluorination Patterns on the Thermodynamics and Kinetics of Benzenesulfonamide Binding to Human Carbonic Anhydrase II

**DOI:** 10.3390/biom10040509

**Published:** 2020-03-27

**Authors:** Steffen Glöckner, Khang Ngo, Björn Wagner, Andreas Heine, Gerhard Klebe

**Affiliations:** 1 Institut für Pharmazeutische Chemie, Philipps-Universität Marburg, Marbacher Weg 6, 35037 Marburg, Germany; steffen.gloeckner@uni-marburg.de (S.G.); khang.ngo@uni-marburg.de (K.N.); andreas.heine@staff.uni-marburg.de (A.H.); 2 F. Hoffmann-La Roche AG, Pharmaceutical Research & Early Development, Roche Innovation Center Basel, 4070 Basel, Switzerland; bjoern.wagner@roche.com

**Keywords:** Carbonic Anhydrase, Macromolecular X-ray Crystallography, kinITC, Thermodynamics, Kinetics, Fluorination, Acidity

## Abstract

The fluorination of lead-like compounds is a common tool in medicinal chemistry to alter molecular properties in various ways and with different goals. We herein present a detailed study of the binding of fluorinated benzenesulfonamides to human Carbonic Anhydrase II by complementing macromolecular X-ray crystallographic observations with thermodynamic and kinetic data collected with the novel method of kinITC. Our findings comprise so far unknown alternative binding modes in the crystalline state for some of the investigated compounds as well as complex thermodynamic and kinetic structure-activity relationships. They suggest that fluorination of the benzenesulfonamide core is especially advantageous in one position with respect to the kinetic signatures of binding and that a higher degree of fluorination does not necessarily provide for a higher affinity or more favorable kinetic binding profiles. Lastly, we propose a relationship between the kinetics of binding and ligand acidity based on a small set of compounds with similar substitution patterns.

## 1. Introduction

The intuitively contradictory nature of the highly polarized carbon-fluorine bond on the one hand and the low polarizability of the fluorine atom itself on the other causes curious results when fluorine is incorporated in organic small molecules. Perfluorination of hydrocarbons leads to fluorocarbons, molecules of hydrophobic and lipophobic nature that neither engage in interactions with water molecules nor with hydrocarbons, but rather keep to themselves and range among the most inert classes of chemicals conceived by men [1]. Instead of perfluorination, medicinal chemists install single fluorine atoms and fluorinated structural motifs at specific sites of a lead scaffold. The interest in the selective installation of fluorine atoms in organic molecules can be readily visualized by the vast number of fluorinating agents and pertinent synthetic strategies as well as the multitude of fluorinated structural motifs [2,3]. There is ample reason for the utilization of fluorine in medicinal chemistry, given the breadth in which this chemical element can alter physicochemical properties of drug-like substances. Fluorine induces conformational changes in alkyl chains, which can be utilized to enforce the biologically active conformation of macrocyclic ligands in solution [3,4]. Additionally, fluorine can alter the pharmacokinetic rate and path of metabolism as well as the distribution of a drug in tissue [5]. The latter comes at the advantage, that the naturally occurring isotope ^19^F can be traced by magnetic resonance spectroscopy and therefore allows the tracking of fluorine-containing compounds in the body [5]. The magnetic resonance of fluorine has also been utilized in NMR-based fragment screening [6]. Furthermore, the introduction of fluorine can have an impact on the nature of protein-ligand binding itself as described, for example, for the first approved fluorinated drug fludrocortisone [3,7]. Opposed to the inert hydrophobic and lipophobic fluorocarbons, the incorporation of fluorinated structural motifs in organic molecules and the accompanying change in charge-distribution can be used to assess the importance of the hydrogen-bond donor ability of OH functionalities of a ligand to bind to a protein as well as protein stability, as the C–F bond formidably mimics the polarity of the C–OH bond, but lacks the hydrogen atom [8,9]. To sum up the practically non-polarizable nature of the fluorine atom and its strongly polarizing effect, the term ‘polar hydrophobicity’ was coined [10]. This inherently contradictory term can be used to increase a molecule’s hydrophobic character and at the same time increase the polarity of proximal functional groups. A prominent example in this respect is the replacement of a carboxylic-acid function with a 2,6-difluorophenol moiety, as difluorination reduces the phenol’s acidity constant (p*K*_a_) [11]. Moreover, fluorine can be used to isosterically replace more common functional groups. The replacement of OH with CF_2_H does not only uphold the C–OH dipole, but additionally mimics the electronic arrangement of the hydroxy function itself: the fluorine atoms replace the oxygen lone-pairs, and increase the acidity of the carbon-bound proton sufficiently to act as hydrogen-bond donor [3]. Another important aspect in this respect is the fluorination of aromatic compounds, which does not only change metabolism and membrane permeability of these substances, but also distinctly changes the aromatic quadrupole moment and in consequence interactions of the π-system. This principle was used exemplarily to fine-tune π-stacking interactions for ligands of Carbonic Anhydrase (CA), the protease Cathepsin L, and the bacterial adhesin FimH [12,13,14]. Furthermore, various fluorination patterns of the phenyl core of benzenesulfonamide (BSA) inhibitors of CA showed an increased affinity compared to their non-fluorinated counterparts [15,16,17]. 

In 2007, Krishnamurthy et al. investigated benzensulfonamides (BSAs) **1**, **3**, **4**, **5**, **8**, **9**, and **14** (Table 1) by isothermal titration calorimetry (ITC) in order to assess the individual thermodynamic contributions of the different types of interactions of these ligands to the overall difference in Gibbs free energy of binding to bovine carbonic anhydrase II (bCAII) and concluded that 65 % were contributed by the interaction between sulfonamide anion and Zn^II^ cofactor, 10 % by the hydrogen-bond network established between ligand and protein, and 25 % by hydrophobic interactions between aromatic ring and protein [18].

**Figure 1:**
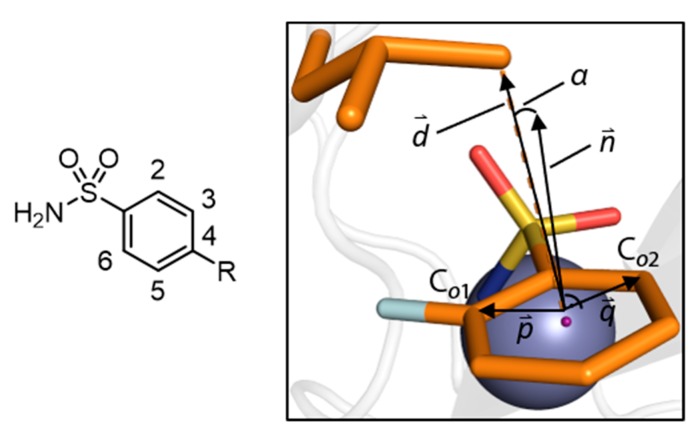
Derivation of metrics listed in Table1 and used for calculations. Calculation of α and the meaning of quantities depicted here and used in the process are described in Materials and Methods.

Scott et al. later used molecules **1**, **3**, **4**, and **5** among other to establish a quantitative structure activity relationship (QSAR) based on thermodynamics of binding of Carbonic Anhydrase inhibitors (CAIs) [15]. In the present study, we conducted a fluorine-scan, similar to the one described by Olsen et al. [19] for the serine protease thrombin, to characterize thermodynamically and kinetically the active site of human Carbonic Anhydrase II (hCAII) by kinITC with fluorinated compounds 3–14 (Table 1). The general structure of investigated compounds, along with geometric measures for quantitative description of binding modes, are given in Figure 1. Considering the circumstance, that comparably little kinetic data derived from ITC experiments has been published so far, it seems appropriate to provide further validation of the method, especially under the prerequisite of an adjusted measurement protocol for the reliable extraction of both thermodynamic and kinetic data. Hence, the above compounds are convenient in this respect, as thermodynamic data for subsets of these are available from the previous studies.

## 2. Materials and Methods 

### 2.1. Calculation of Interface Areas

Interface areas were calculated with the Linux version of the program dr_sasa in mode 4 [24].

### 2.2. Protein Expression and Purification

Human Carbonic Anhydrase II (hCAII) was expressed and purified according to a protocol by Gaspari et al. [25]. with additional 60 µM ZnCl_2_ (Sigma Aldrich, St. Louis, MO, USA) in the overnight culture and during cell growth, while protein expression was carried out with 1 mm ZnCl_2_, based on the work of Cimmperman et al. [26]. Protein material was dialyzed against 10 mm HEPES (Carl Roth, Karlsruhe, Germany) buffer at pH 7.8 at 26 °C after the final purification step. The dialysis buffer was filtrated through a Thermo Scientific Nalgene Rapid-Flow PES Bottle Top Filter with a pore size of 0.2 µm (Thermo Fisher Scientific, Waltham, MA, USA) and used for the measurements. Dialyzed protein was aliquoted and stored at −80 °C.

### 2.3. Isothermal Titration Calorimetry

Measurements were carried out on an ITC200 (Malvern, Kassel, Germany) at 25 °C with a stirring speed of 1000 rpm and 180 s of spacing between consecutive injections (cf. Figure S1, Supplementary Materials). The instrument response time was determined to 4.36 ± 0.1 s as described in the Supplementary Materials (cf. Figure S2, Supplementary Materials). This value is in accordance with values generally expected for an ITC200. 

The evaluation of ITC data with respect to kinetic parameters is based on the fact that ITC data bear inherent kinetic information, as the power signal resulting from a reaction is recorded over time [27]. With an increasing number of injections, the equilibration time of the signals is prolonged, which is visible by the return to baseline level, until the point of mid-titration (the inflection point of the fitted isotherm). After mid-titration, the equilibration time for the remaining signals declines. Thus, kinetic information is present throughout the whole titration experiment, but with different accuracies, depending on the location of the injections in the course of the experiment. Signals before mid-titration differ distinctly from baseline level and therefore from instrument noise and have a higher certainty than signals after mid-titration and are in consequence essential for kinetic analysis. This may become problematic, however, if the experimenter is facing the reality of an only partially active protein sample, which is not uncommon [28]. A reduced fraction of protein competent to bind the ligand will reduce the stoichiometry of binding and therefore the number of signals prior to mid-titration, which can render kinetic analysis hardly possible. As this was the case for hCAII expressed in our laboratory, we developed a novel titration protocol to accommodate this.

Titration experiments were designed in a way that ensured a sufficient number of injections prior to mid-titration and was reported in one of our previous studies [20]. All compounds except **6** and **10**–**13** were commercially available. Synthesis of compounds **6** and **10**–**13** are located in the Supplementary Materials. The purities of all compounds were determined to be larger than 95% by analytical HPLC and were taken into account for the preparation of 30 mm DMSO (Carl Roth, Karlsruhe, Germany) stocks.

Samples were prepared under the prerequisite of a total DMSO content of 3% (*v*/*v*) and a total sample volume of 300 µL. The ligand DMSO stock was diluted further for a total volume of DMSO of 9 µL and mixed with 291 µL of buffer. Protein stock solution was mixed with buffer and subsequently 9 µL of DMSO. Prior to usage, sample solutions were vortexed shortly and centrifuged. 

Prior to loading the measurement cell was rinsed with demineralized water and buffer. An excess of sample solution in the filling cone was removed. Loading of the syringe was succeeded by purging and refilling. A downward movement of the plunger corresponding to 0.03 µL was performed [29]. An injection of 0.3 µL preceded every titration experiment and was discarded before analysis. Due to the high affinity of compounds **11**–**13**, a displacement setup was necessary for their characterization. Displacement titrations were set up according to suggestions by Velazquez-Campoy and Freire [30]. Then, 4-carboxy benzenesulfonamide (4CBS, Sigma Aldrich, St. Louis, MO, USA) was used as reference ligand to inhibit hCAII. The hCAII-4CBS complex was then titrated with the ligand of interest.

### 2.4. Isothermal Titration Calorimetry (ITC) Data Analysis

The AFFINImeter software suite (Version 2.1710, S4SD–AFFINImeter, Santiago di Compostella, Spain) was used for the analysis of titration data [27,31,32,33]. Thermodynamic data were determined with the global fit approach from the individually processed experimental data. Globally determined thermodynamic data were used to fit kinetic data anew. For the global analysis of alkylated compounds **11**–**13**, three direct titrations of the ligand of interest, three direct titrations of 4CBS and at least two displacement titrations were included. A blank titration of ligand in buffer, ligand into 4CBS, and 4CBS in buffer were additionally subtracted from the respective titration data before global fitting. Thermodynamic and kinetic values are provided in Tables S2–S4 in the Supplementary Materials. Raw and processed experimental data, isotherms and equilibration-time curves can be found in the Supplementary Materials. 

### 2.5. Macromolecular Crystallography

Crystallization of hCAII was carried out in a hanging-drop setup in buffer containing 2.7 m ammonium sulfate, 0.1 m TRIS (Carl Roth, Karlsruhe, Germany) at pH 7.8, which was additionally saturated with *para*-chloromercuribenzoic acid (Sigma Aldrich, St. Louis, MO, USA), using 24-well plates (Hampton Research, Aliso Viejo, CA, USA) with siliconized cover slips (Jena Bioscience, Jena, Germany). 2.0 µL of protein solution (*c* = 10 mg mL^−1^) in the final purification buffer were mixed with 2.0 µL of the above crystallization buffer on a cover slip and placed over the well containing 0.5 mL of crystallization buffer with silicon grease as sealant. Crystallization occurred within a day. Soaking was done in a buffer containing 3.0 m ammonium sulfate (Carl Roth, Karlsruhe, Germany) and 0.1 m TRIS (Carl Roth, Karlsruhe, Germany) at pH 7.8 overnight. Flash freezing in liquid nitrogen was preceded by submersion of the crystal for 5 s in a solution containing 3.0 m ammonium sulfate, 0.1 m TRIS, d-glucose (anhydrous, Fluka) 25% (*w*/*v*) at pH 7.8 which was additionally saturated with the respective ligand.

Diffraction data were collected on BL14.1 and 14.2 at the BESSY II electron storage ring operated by the Helmholtz-Zentrum Berlin (Berlin, Germany) [34]. Data were indexed, integrated, and scaled with XDS and XDSAPP2.0 [35,36]. Structures were solved by molecular replacement with PDB-entry 3KS3 (PDB: Protein Data Bank) in Phaser from the CCP4 suite [37,38]. Crystallographic models were built in Coot and subsequently refined in Phenix until *R*-factors converged [39,40]. ReadySet from the Phenix suite was used to add riding hydrogen atoms [40]. The final models along with structure factor files were deposited in the PDB with the accession codes shown in Table 1. PyMOL was used to create crystallographic images [23].

Statistics for diffraction data and refinement are provided in the Supplementary Materials in Table S1.

### 2.6. Calculation of the Deviation Angle α

The meaning of the quantities and objects referred to in this paragraph are shown in Figure 1. The deviation angle α between the normal vector ($\vec{n}$) of the phenyl ring plane and the deviation vector ($\vec{d}$) was measured for the reference conformation of Leu198 using PyMOL with the NumPy library for vector construction and the vg library for vector operations [21,22,23]. Directional vectors for the phenyl ring plane ($\vec{p}$, $\vec{q}$) were calculated by subtracting the position vector of the phenyl centroid from the respective position vectors of the *ortho* carbon atoms (C*_o_*_1_, C*_o_*_2_). $\vec{n}$ was determined *via* the cross product of the resulting vectors with the cross function from the vg library. $\vec{d}$ was calculated by subtraction of the position vector of the phenyl centroid from the position vector of the respective C_δ_ atom of Leu198.

### 2.7. pK_a_ Measurements

The p*K*_a_ values were determined by SiriusT3 Fast UV p*K*_a_ method, which is based on the spectrophotometric (UV-metric) titration method reported in reference [41]. The compound of interest was prepared as 10 mm stock solution in DMSO and a fixed aliquot size of 3 μL was added to 1.5 mL of water containing 0.15 m KCl as background electrolyte. The pH of the dilute sample solution was adjusted to pH 2 by addition of 0.5 m HCl and then titrated with standardized base (0.5 m KOH) to pH 12 at 25 °C under argon atmosphere. The SiriusT3 Fast UV p*K*_a_ method uses a proprietary linear buffer system adapted from the literature [42] to achieve rapid stabilization of the pH after each titrant addition. During the titration UV/vis spectra were collected as a function of the pH readings. The p*K*_a_ of the sample is calculated from the pH readings and UV spectra collected.

### 2.8. Associated Content

#### PDB Accession Codes

Atomic coordinates and experimental details for the crystal structures of **1** and **2** are available under the PDB entries 6GDC and 6GM9 and were published previously [20]. Crystal structures for compounds investigated herein will be released upon publication under the PDB entries listed in Table 1.

## 3. Results

### 3.1. Crystallographic Data

**Figure 2:**
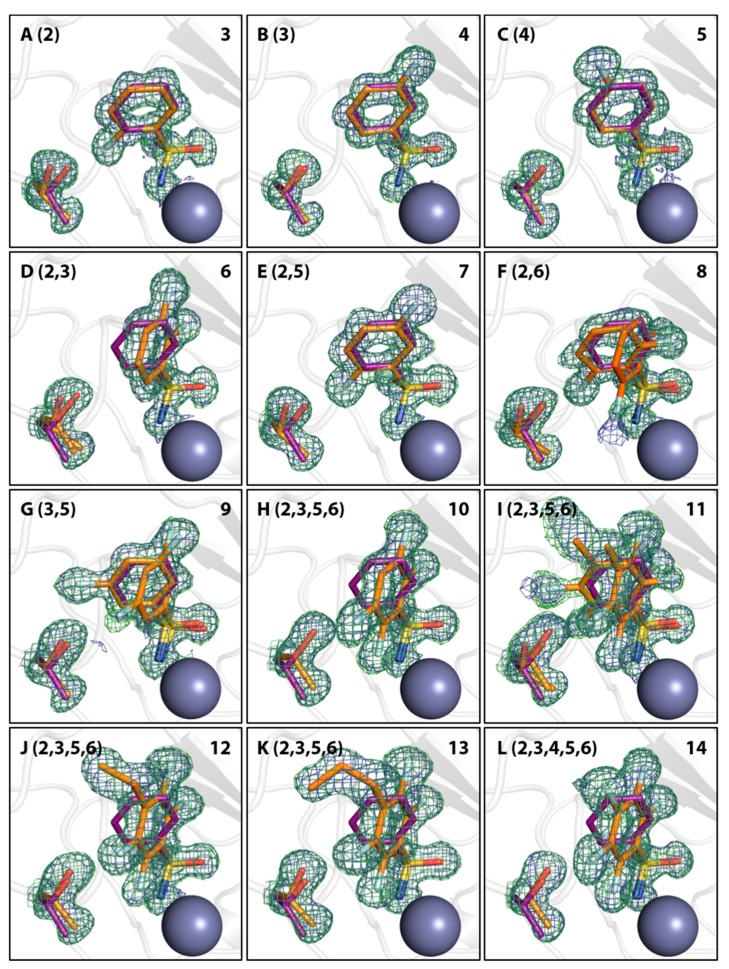
Binding modes of fluorinated compounds and side chains of the respective Thr200 residue in orange in images (**A**–**L**). Fluorinated positions at the phenyl ring relative to the sulfonamide bearing carbon atom (position 1) are given in parentheses. Compound numbers according to Table 1 are given in the upper right corner. Compound **1** is shown in all images for comparison in purple. Omit 2mFo-DFc density is shown in blue at 1 σ and omit mFo-DFc density in green at 3 σ. The Zn^II^ cofactor is shown as gray sphere. Notably, fluorination patterns in compounds **8**, **9**, and **11** induce a second alternative binding pose.

Crystallographic models of compounds 3, 4, 5, 8, and 9 in complex with hCAII were already available in the PDB under the accession codes 2WEG, 2WEO, 1IF4, 1IF5, and 1IF6. However, structures for all compounds in Table 1 were newly determined to improve the resolution and maintain the same crystallization and soaking conditions for all complexes. In general, two different conformations of fluorinated compounds in the hCAII active site were observed, which orient the phenyl ring differently, as depicted in Figure 2 **3**, **4**, **5**, **7**, **8**, **9**, and **11** (mean value of torsion angle *τ*_mean_ = 54.2° with a standard deviation of ± 3.4°), and mimics that of non-fluorinated compound **1**, which will be referred to as ‘reference conformation’. The deviating orientation adopted by compounds **6** and **8**–**14** (*τ*_mean_ = 2.2° ± 1.9°) will be referred to as ‘alternative conformation’. It is worth noting that the reference conformation enables the key interaction between the aromatic portion and the side chain of Leu198 [43]. In Table 1, the torsion angle *τ* between the nitrogen, coordinated to the Zn^II^ cofactor, sulfur, the *ipso*-carbon atom C1 and C2 are listed. The contact between the aromatic ring and Leu198 results in a short distance to one of the terminal C_δ_ methyl groups, the distance between C_δ_ and the centroid of the ring is listed in Table 1, together with the deviation from the direction of the normal vector of the phenyl-ring plane. In the reference conformation, the angular deviation is very small. In the second alternative conformation, adopted by compounds **6** and **8**–**14**, it amounts to approximately 40°.

**Figure 3:**
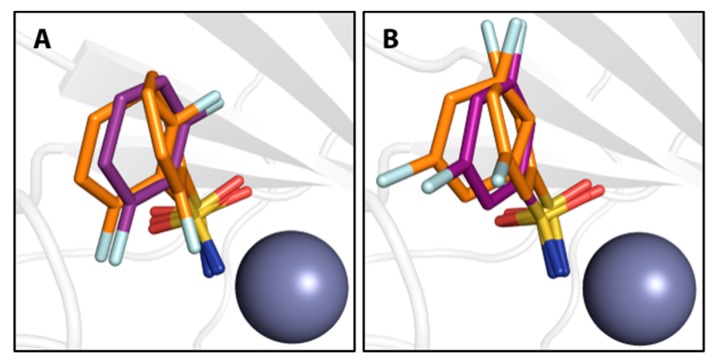
Compounds **8** (**A**) and **9** (**B**) in orange compared with ligand conformations observed in previously determined PDB entries 1IF5 and 1IF6 (purple), respectively. The Zn^II^ cofactor is shown as gray sphere.

At first, monofluorinated compounds **3**–**5** will be considered. Comparable to **1** and **2**, compound **5** merely orients its *para* fluoro substituent toward the entrance of the active-site funnel, virtually adopting the same geometry as 1 and 2 with respect to both, the ligand pose and the orientation of the adjacent residue Thr200. The latter residue is identically oriented as in the *apo* hCAII structure (PDB code 3KS3). Compounds **3** and **4** (Figure 2) orient the fluorine atoms in opposite directions. Notably, the fluorine atom of **3** is located within a distance of the hydroxy function of Thr200 that allows for the inference of a hydrogen bond, as already pointed out by Scott et al. [15]. Compound **4** positions the fluorine atom toward the rim of the hydrophobic wall. This seems reasonable to expect with respect to the preference of fluorine atoms and fluorinated motifs to occupy hydrophobic pockets [3]. With this binding pose, **4** adopts a conformation very similar to **1** and **2** and Thr200 also remains in an unchanged orientation, whereas **3** pushes Thr200 slightly out of position (rmsd ~0.2 Å), likely due to steric repulsion with the *ortho* fluoro substituent. Interestingly, the combination of **3** and **4** with respect to the fluorination patterns to reveal the difluoro derivatives 6 and 7 results in different orientations of the latter two compounds. Whereas **7** is virtually a superposition of the binding modes of **3** and **4**, **6** adopts the alternative orientation of the phenyl ring. Supposedly, this shift results from steric repulsion with the terminal methyl group of Val121. With respect to Thr200, **7** induces the same geometry already observed for **3**. The binding of **6**, however, entails two different movements of Thr200, one of which can be described as toward the ligand, which can be caused by an attractive interaction. Notably, compounds **8**, **9**, and **11** adopt both orientations of the phenyl ring in the crystal structure. Importantly, the alternate conformations found for compounds **8** and **9** merit the renewed production of crystal structures herein, seeing that no alternate conformations could be resolved in the already deposited models 1IF5 (**8**) and 1IF6 (**9**, Figure 3).

**Figure 4:**
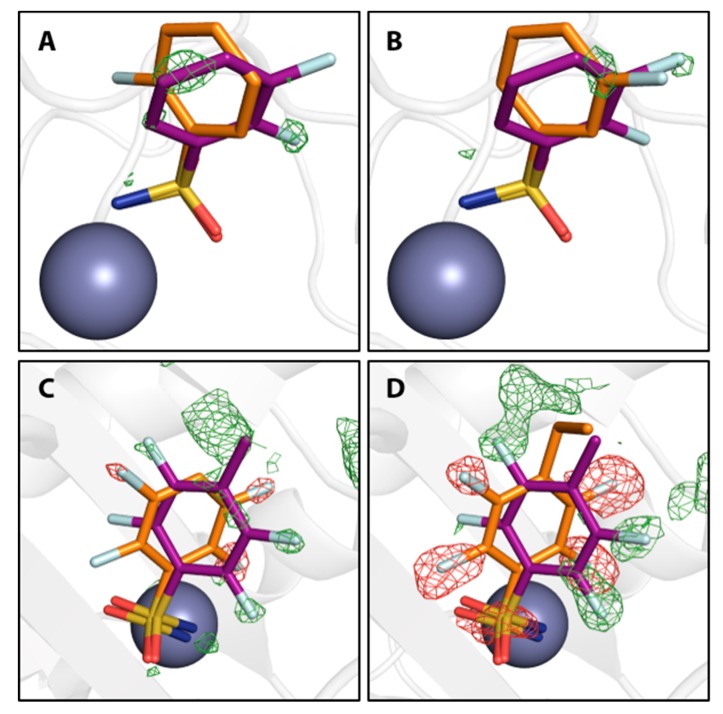
Compounds **3**, **4**, **9**, and **12** in orange with putative additional binding conformations. Positive and negative mFo-DFc maps are shown in green and red at 3 σ and -3 σ, respectively. Compounds **3** (**A**) and **4** (**B**) are shown in comparison with compound **6** in purple to indicate the second conformation. Compounds **9** (**C**) and **12** (**D**) are shown in comparison with compound **11** in purple to indicate the second conformation. The Zn^II^ cofactor is shown as a gray sphere.

Furthermore, difference electron density of the final models indicates that a second binding conformation seems possible also for compounds **3**, **4**, **11**, and **13**, although the difference density is too weak to properly allow modeling of the second, definitely minor, populated arrangement. Reconsidering the interpretation of the density for **3** and **4**, a second putative binding conformation appears visible in the positive difference density, negative difference density indicates overpopulation for the modeled conformations of compounds **11** and **13** (Figure 4).

### 3.2. Movement of Thr200

The notion that hCAII has a highly rigid binding site, which is not influenced structurally by the binding of ligands, needs to be relativized, seeing that almost all of the diversely fluorinated BSAs examined herein cause a movement of Thr200 [44]. The orientation of Thr200 in the complex of hCAII with **1** and in the unliganded protein will also be referred to as the reference state in this respect. It was found that ligands distinctly displace Thr200 with respect to its position in the reference state in two manners (Figure 2), a quantification by RMSD analysis relative to the hCAII–**1** complex (6GDC) can be found in Table 1. The first one can be described as a movement away from the Zn^II^ cofactor, the second one as a movement toward it. Binding of compounds **3**, **4**, **7**, **8**, and **9** entails movement away from the zinc ion. Compounds **10**, **11**, **12**, and **13** cause a movement toward the zinc ion. Given the comparably low degree to which **4** displaces the amino acid, it is still reasonable to assume the same geometry as in the reference structure. The same holds for **5**. In case the *ortho* position is occupied by a fluorine atom, the slightly larger size of F compared to H results in a shift of Thr200 (**3** and **7**) away from the Zn^II^ cofactor. Obviously, an additional o-fluorine attached to the monofluoro-**4** results in a strong rotation of the phenyl ring and the *ortho* fluorine atom is accommodated in a small niche next to Val121. Thr200 is shifted away from the Zn^2+^ ion. The di-*ortho* and di-*meta* derivatives **8** and **9** bind with two conformers simultaneously. In the di-*ortho* case, Thr200 is shifted, whereas the di-*meta* derivative binds with Thr200 in nearly unchanged orientation. The series of **10**–**13** share the 2,3,5,6-tetrafluoro pattern. Apart from **13**, they all share two binding poses. However, they are differently populated. The *p*-Me derivative shows both orientations, interestingly with reversed occupancies compared to **8** and **9**. In the case of **10** and **12**, the second orientation is only found with minor occurrence (Figure 4). Interestingly, **13** shows only one orientation of the phenyl ring. Possibly, this is caused by the circumstance that the attached *n*-propyl group at C4 adopts a gauche conformation and occupies some space required for the placement of the ligand in the alternative conformation. Given the observation, that ligands with only one modeled orientation can have a putative second binding orientation (Figure 4), this can be taken as a hint for a putative second orientation of Thr200 that might be caused by the indicated, but not modeled, second orientation of the ligand.

### 3.3. Accommodation of a Fluorine Atom in a Hydrophobic Pocket

**Figure 5:**
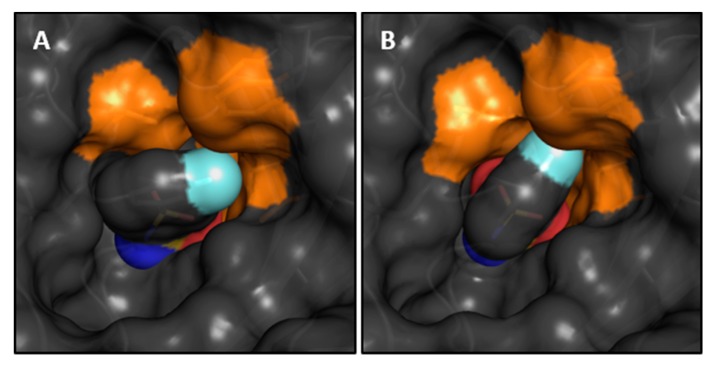
Hydrophobic pocket in orange, that accommodates *meta* and *ortho* fluorine substituents, exemplarily depicted with compounds **4** (left) and **6** (right) which adopt either the reference (*τ* ~0°) or alternative conformation (*τ* ~60°).

As reasoned above, the different orientation of **6** compared to **7** is likely the avoidance of steric repulsion between the *ortho* fluorine atom and the side chain of Val121. The movement of the ligand furthermore entails a deeper burial of the *meta* fluorine substituent in the cavity bounded by the side chains of residues Val121, Phe131, Leu141, Val143, and Leu198 (Figure 5).

This suggestion is supported by the circumstance, that also compound **8**, which bears no *meta* substituent, partially adopts the alternative orientation. Table 1 shows values for the interface area between fluorine atom and protein the fluorine atom accommodated in the hydrophobic pocket defined above. It is noteworthy, that for the alternative orientation (**6**, **9**–**14**) the interface values for the accommodation of either *ortho* or *meta* substituent are larger than for the standard orientation (**4**, **7**) and in a similar range across either the *meta* or *ortho* substituted molecules. Although the elucidation of universal structural rules is impeded by the presence of an alternative binding mode, the binding of compounds **3**, **4**, **6**, and **7** reveals, that the preference of a fluorine atom in *meta* position to bind to the hydrophobic pocket exceeds that of a fluorine atom in *ortho* position to be oriented toward Thr200.

The comparison of compounds **6** and **7** furthermore allows for the conclusion, that the preference for a fluorine atom in *meta* position to bind to the hydrophobic pocket is stronger than that of a fluorine atom in *ortho* position to be oriented toward Thr200.

**Figure 6:**
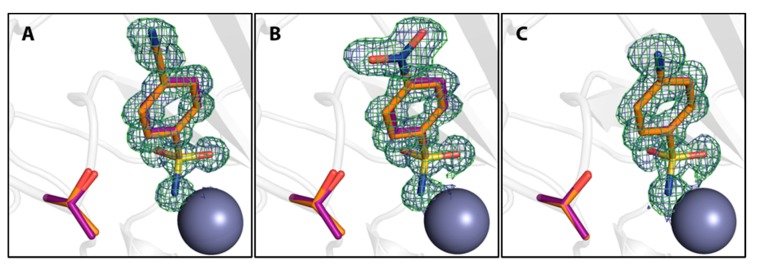
*Para-substituted* benzenesulfonamides (BSAs) **15** (**A,** 4-cyano-), **16** (**B,** 4-nitro-), and **17** (**C,** 4-amino-) in orange with omit electron density maps in blue at 1 σ (2mFo-DFc) and 3 σ (mFo-DFc) and the side chain of Thr200. Compound **1** is shown as referenced in purple. The Zn^II^ cofactor is shown as gray sphere.

Given the complex picture painted by the various, above-described substitution patterns, which resulted in different binding poses and even induced a shift of Thr200, we decided to investigate and furthermore characterize the structures and thermodynamic and kinetic binding signatures of hCAII complexes of merely *para*-substituted BSAs **15**–**17**, which would putatively maintain the same binding pose as unsubstituted BSA **1**, and furthermore not displace Thr200 (Figure 6), but alter the chemical properties of the ligands, e.g. in terms of their polarity or hydrophobicity. As anticipated, the crystal structures underscore conserved binding modes and the attached 4-substituents are all placed toward the entrance of the funnel-shaped binding pocket.

### 3.4. An Unexpected Dimerization Product

**Figure 7:**
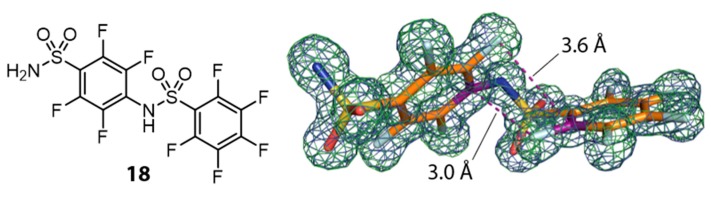
Valence bond formula of dimerization product **18** (left) and crystallographic model from the Human Carbonic Anhydrase II (hCAII) complex with 2mFo-DFc map contoured at 1 σ and mFo-DFc map in green at 3 σ (right). Carbon atoms are shown in orange, fluorine atoms in light blue. Carbon atoms used to display intramolecular π-stacking are shown in purple. π-Stacking interactions are indicated as purple dashed lines with the respective distances between atomic positions of F and C.

Electron density for hCAII in complex with compound 14 revealed evidence that not only the catalytic center next to the zinc ion is accommodated but that a further intriguing molecule, that represents a dimer (in the following named 18) of 14, is found in the crystal structure. It features intramolecular edge-to-face π-stacking interactions. Figure 7 shows dimer 18 and its crystallographic model geometry suggested by omit electron density maps.

**Figure 8:**
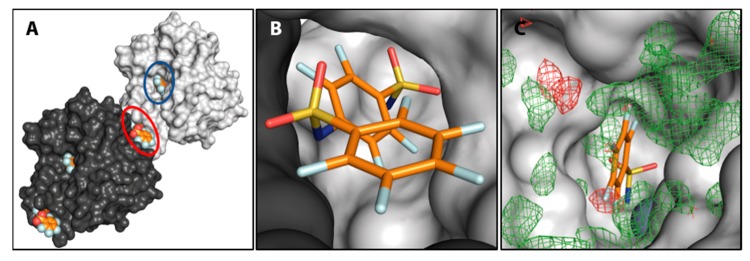
(**A**) Surface representation of two hCAII crystal mates that enclose dimer **18** between them. The binding site of **18** and the active site including a model of **14** are indicated by red and blue ellipses, respectively. (**B**) Close-up of the binding site of **18** between two hCAII crystal mates. (**C**) Residual mFo-DFc density at 3 σ (green) and −3 σ (red) above the active site with the modeled monomer **14** (for difference electron density allowing to model **14**, see Figure 2L).

Considering the van der Waals radii of a C-atom in benzene (r_vdW_(C_ar_) = 1.77 Å) and a phenyl bound F-atom (r_vdW_(F_ar_) = 1.47 Å), the sum of 3.24 Å is larger than the intramolecular distance of 3.0 Å found for the binding mode of 18 [45]. It depicts the electron deficient nature of the aromatic moieties, which leads to a decreased electron density around the carbon atoms. Compound 18 binds in a second, surface-exposed binding site, covered by a second crystal mate. There it engages in both, classical hydrogen bonds and interactions often observed between fluorine atoms and proteins. Figure 8 shows an overview of two hCAII symmetry mates clamping molecule 18 and a close-up view of the binding site. With respect to the catalytic center, a fair amount of difference density indicates binding of a ligand. The dimer 18, however, could not be modeled in the active site, although pronounced residual mFo-DFc density above the modeled monomer 14 suggests that 18 also populates the active site, albeit with a very low occupancy (Figure 8C).

It was not clear whether the dimer **18** formed in the soaking drop or had already been present in the commercially available solid. Consequentially, it was desisted from further usage of **14** for ITC experiments, given the unclear quality and purity of the sample.

### 3.5. Thermodynamic Results

**Figure 9:**
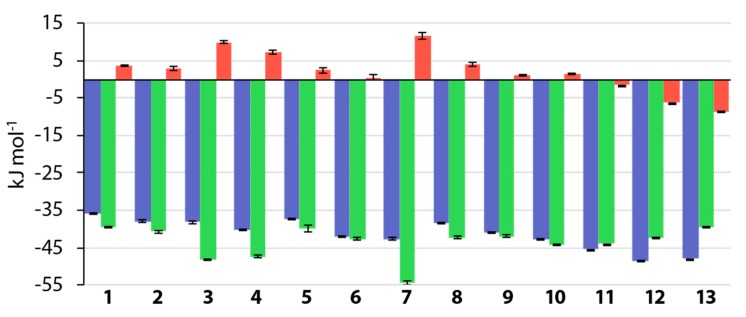
Thermodynamic signatures of compounds **1**–**13** measured in 10 mm HEPES buffer at pH 7.8 with Δ*G*° in blue, Δ*H*° in green, and –*T* Δ*S*° in red. The values for **1** and **2** were taken from reference [20]. For **1**–**10**, globally fitted values are given. Error bars represent the standard error of measurement for these compounds, based on three measurements. Compounds **11**–**13** were characterized by a displacement experiment with subsequent global fitting, as they were not characterizable directly due to their high affinities. For these compounds, the globally fitted values of *K*_a_ and *ΔH°* and the values of *ΔG°* and –*T* Δ*S°* calculated from the former two are given. Error bars represent the error of global fitting for *K_a_* and Δ*H*° and the error calculated from those of the former two for –*T* Δ*S*°. Numerical values are provided in the Supplementary Materials in Table S1.

Titrations of hCAII with a different batch of 1 resulted in a molar amount of transferred protons of *n*_p_ = −0.1 moles L^−1^ (Supplementary Materials, p. 3), meaning that on molar scale 0.1 protons are transferred to the surrounding buffer per formed hCAII–1 complex. As sulfonamides are known to bind hCAII as anions, a proton from the sulfonamide group must be released into the surrounding medium. Under the assumption, that the ligand associates with the protein in the uncharged state as described by Gaspari et al., the proton needs to be transferred to the surrounding medium from within the active site. The reason for the small detected amount of 0.1 moles L^−1^ supports the assumption that, prior to sulfonamide binding, the fourth vertex of the tetrahedral Zn^II^ complex is occupied by a hydroxide ion. The hydroxide ion can react with the proton from the sulfonamide group to form water. This means that it is not necessarily protonated *via* a buffer molecule; overall, however, this does not alter the recorded heat signal in a buffer-dependent manner. In consequence, the thermodynamic signatures, depicted in Figure 9, which describe the entire complex formation process of the investigated compounds, can be expected to be not overlaid by a protonation step.

The addition of a fluorine atom in *para* position of **1** entails a moderate increase in binding free energy (ΔΔ*G*°**_1_**_→**5**_ = −1.3 kJ mol^−1^), whereas the addition of a fluorine atom to ligand **1** in *ortho* position leading to compound **3** entails a distinct change in the thermodynamic binding profile compared to that of **1**, with a distinctly stronger enthalpic advantage and entropic penalty, which lead to a slightly larger gain in Δ*G°* (ΔΔ*G*°**_1_**_→**3**_ = −2.2 kJ mol^−1^). A similar development holds true for *meta* fluoro-**4** to a lesser extent in the enthalpic and entropic parameters, but a further increase in overall affinity (ΔΔ*G*°**_1_**_→**4**_ = −4.2 kJ mol^−1^). Interestingly, the combination of **3** (2-F) and **4** (3-F) to reveal **7** (2,5-F), which adopts the same conformation (*τ* ~0°) and binding pose as the former three (**3**, **4**, **5**) and orients both fluorine atoms accordingly, amounts to more than the sum of its parts in the thermodynamic signatures. Both enthalpic benefit and entropic penalty are larger than for either of the former three compounds and yield an overall gain in binding free energy (ΔΔ*G*°**_1_**_→**7**_ = −6.8 kJ mol^−1^). Mono-*ortho* and mono-*meta* fluorination, as well as the 2,5-difluorination, seem to be the enthalpically favored substitution patterns in the investigated series, whereas the *para* fluorination in **5** has no enthalpic advantage. However, the alternative combination of **3** and **4** to form the *ortho*-*meta* **6** entails a distinct diversion from the enthalpic and entropic signatures of the parent compounds. Hardly any entropic contribution is observable in HEPES buffer, rendering **6** a merely enthalpic binder within the investigated series of molecules, with a similar value of Δ*G*° as **7**. Notably, a second *ortho* fluorine atom as in compound **8** reduces the entropic penalty of compound **3**, but also the enthalpic benefit, which leaves the free energy of binding virtually unaltered compared to **3**. Di-*meta* fluorination in **9** has a similar effect, given the different enthalpic and entropic signatures of monofluorinated **4** and difluorinated **9** and their highly similar values of Δ*G*° (ΔΔ*G*°**_4_**_→**9**_ = −0.7 kJ mol^−1^). Tetrafluorination of **1** to afford **10** does not provide a distinct increase in affinity over related compounds, seeing that the addition of two fluorine atoms to **6** yields an almost negligible increase in Δ*G*° with an increment of ΔΔ*G*°**_6_**_→**10**_ = −0.5 kJ mol^−1^. In this particular case, it is obvious, that the addition of two additional fluorine atoms is thermodynamically futile. The addition of a methyl (**11**) or ethyl group (**12**) in 4-position, however, provides an increase in affinity for the tetrafluorinated scaffold. Given the two binding modes of the ethyl derivative of **12**, however, it is questionable whether an increased ability to form interactions with the hydrophobic wall, as shown in previous studies, is the only reason for the increase in affinity [20,25]. It must certainly play a role, however, given the circumstance, that compound **13**, which does not show a second binding mode that would allow for an interaction between the *n*-propyl chain and the hydrophobic wall, has a slightly decreased affinity compared to **12** (ΔΔ*G*°**_13_**_→**12**_ = −0.5 kJ mol^−1^).

**Figure 10:**
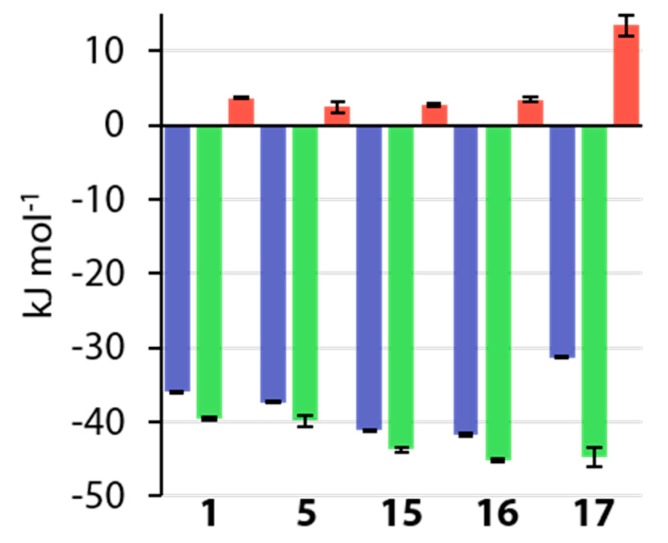
Comparison of thermodynamic signatures of *para*-substituted compounds **1**, **5** and **15**– **17**.

A comparison of *para*-substituted compounds **5** and **15**–**17** and unsubstituted prototype **1** shows, that for electron withdrawing groups, a gain in affinity is mainly enthalpy-driven, while an electron-donating group in **17** causes a loss in affinity due to an entropic penalty (Figure 10).

### 3.6. Kinetic Results

The kinetic *k*_on_ and *k*_off_ rates were determined by our newly developed titration protocol using kinITC [20]. The obtained data are summarized in Table S4 in the Supplementary Materials. Overall, *k*_on_ scatters over a range of four orders of magnitude (Δ*k*_on_ = 15.5 10^4^ m^−1^s^−1^) and *k*_off_ less with Δ*k*_off_ = 1.7 10^−2^ s^−1^. Given the circumstance that the variety of different substituents and substitution patterns investigated herein inevitably entail varying acidities of the BSA ligands, their p*K*_a_ values were measured, which are listed in Table 2.

**Figure 11:**
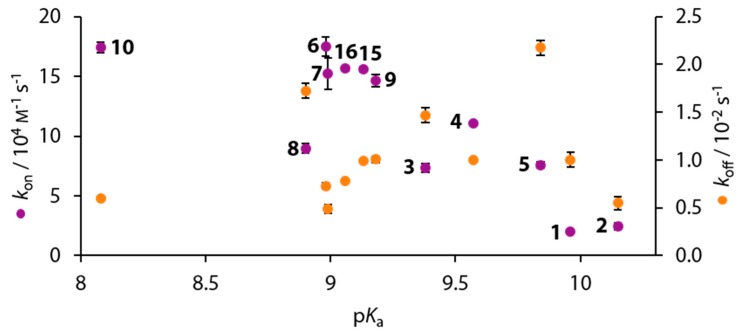
Kinetic signatures *k*_on_ (purple, left axis) and *k*_off_ (orange, right axis) of compounds **1**–**10**, **15**, and **16** plotted against the p*K*_a_ value.

Compared to the non-fluorinated ligands **1** and **2** no clear-cut correlation is obvious, and a large scatter of the fluorine-containing ligands suggests a complex structure-kinetic correlation (Figure 11). 

As compounds **11**–**13** had to be characterized in a displacement setup, kinetic data could not be derived for these compounds. Furthermore, the extraction of kinetic data was not possible for compound **15**.

## 4. Discussion

### 4.1. Comparison of Thermodynamic Data with Earlier Studies

**Figure 12:**
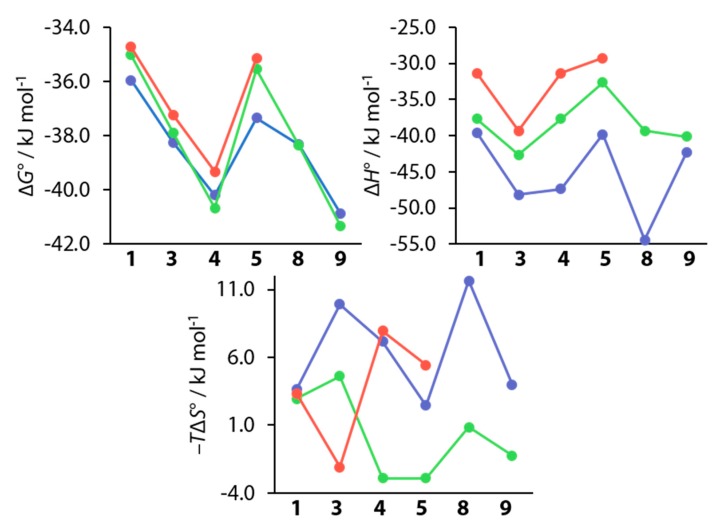
Comparison of thermodynamic values recorded in this work (blue) with data from studies by Krishnamurthy et al. (green) and Scott et al. (red).

As mentioned above, ligands **1**, **3**, **4**, **5**, **8**, and **9** had already been investigated previously by Krishnamurthy et al. and furthermore by Scott et al., who included compounds **1**, **3**, **4**, and **5** in a study of variously substituted CAIs [15,18]. Figure 12 shows a comparison of the thermodynamic data of these studies with the respective data collected in the course of this work.

The values for Δ*G*° closely resemble each other with the largest difference of ΔΔ*G*° = −2.1 kJ mol^−1^ found for compound **5** between the value presented herein and that of Scott et al. [15]. It needs to be noted here that Krishnamurthy et al. used bCAII instead of hCAII, which are, at least structurally, largely similar [18,44]. Whether this similarity translates to the thermodynamic signatures is difficult to estimate. The enthalpic contributions differ by amounts of up to almost 20 kJ mol^-1^. This can be expected, seeing that the studies were conducted in different buffers at different pH values, and that the previously mentioned small amount of protons can be expected to be transferred between a CA–BSA complex and the surrounding buffer [46,47]. Generally, the enthalpic trends are roughly maintained. However, given that the offsets are not constant, other differences between the studies must be expected. The deviations between all three studies become even more pronounced in the entropic term, given that only some trends can be crudely reproduced. However, seeing that the entropic term is calculated from the directly measured values of *K*_a_ and Δ*H*°, errors in these quantities will strongly influence the entropic term. Seeing that the trends for the directly measured quantities *K*_a_ (and thereby Δ*G*°) could be reproduced, this study is a further example for the validity of the measurement protocol, that was improved for kinetic analysis of ITC data. Krishnamurthy et al. investigated the above compounds in order to partition the contribution of electrostatic and hydrophobic interactions on the ligand’s structural elements, arguing that this was possible due to the same orientation of the BSA cores of all ligands [18]. Given the finding that **8** and **9** do, however, adopt two binding modes, and that a second conformation can also be inferred for **3** and **4**, it is questionable whether a partitioning in this manner can be conducted this easily. In addition, the argument does not take into account the special nature of fluorine elaborated above, which greatly differs from that of hydrogen. The various orientations of fluorine atoms in the active site by different ligands enable different interaction patterns, and they cause structural changes of the protein and different binding modes of the ligands. It therefore seems questionable that the overall free energy of binding can be easily partitioned on the individual structural motifs, given the large variety of interaction patterns across the ligand series.

### 4.2. Comparison of Crystallographic with Thermodynamic Data

Overall, two conformers are observed in the studied complexes. The phenyl moiety adopts an eclipsed arrangement with respect to the attached sulfonamide group, giving rise to two conformational families. Either the S-N bond (*τ* close to 0°) or one of the S=O bonds is in coplanar arrangement with the phenyl ring (*τ* approximates 60°). The two additional substituents at sulfur adopt an orientation with approximately 60° above and below the phenyl plane. The two binding-pose orientations correlate either with an occupancy of the fluorine substituent in a small hydrophobic niche next to Val121 and Leu141 or seemingly more deeply buried in a pocket below Phe131 and adjacent to Val143. The two orientations also correlate with an angular deviation of the terminal C_δ_-methyl group at Leu198 from the normal vector perpendicular to the phenyl ring. The 4-substituent orients toward the opening of the funnel-shaped active-site pocket and is partly exposed to the solvent environment. Likely due to steric conflicts in some of the complexes, significant spatial shifts are recorded for the placement of Thr200, taking the *apo* protein as reference.

Assuming that the crystallographically observed binding modes reflect geometries relevant for the thermodynamic signatures of the binding ligands, our data suggest that the occupancy of the hydrophobic niche next to Val121 and Leu141 by a fluorine substituent is enthalpically favored compared to the placement in the alternative pocket below Phe131. Opposing trends are found for the entropic signature, slightly compensating the enthalpic advantage. The seemingly deeper burial of the fluorine substituent may be compensated by a more costly steric placement with an increased contact surface to the protein (Table 1). However, these opposing effects are difficult to translate directly into changes of the thermodynamic profiles. Remarkably, the placement of an *ortho* fluoro substituent next to Thr200 results in a shift of the latter residue, however, without an enthalpically detrimental effect. This might be compensated for by a putative hydrogen bond between the fluorine atom and the hydroxyl side chain function of Thr200, already suggested by Scott et al. [15].

### 4.3. Comparison of Crystallographic with Kinetic Data

**Figure 13:**
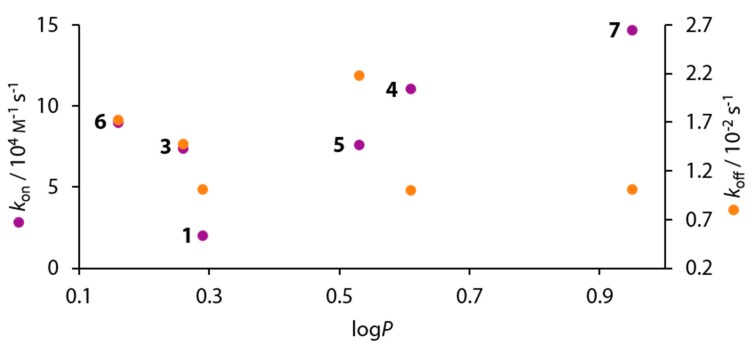
Plot of *k*_on_ (purple, left ordinate) and *k*_off_ (orange, right ordinate) against log*P* shows, that hydrophobicity is not a determining factor for the binding kinetics of fluorinated BSAs.

An inherent problem in the process of establishing structure-kinetic relationships is the contrary nature of structure on the one hand, and the processes of association and dissociation on the other. While our structural knowledge is mostly elucidated in the solid state, the association and dissociation of molecules comprises several subsequent states usually in solution, whose time-dependency cannot be elucidated in the solid state. For the interpretation of kinetic data, structural information about the time-limiting step along the association and dissociation process must be known. While in the case of 4-alkyl substituted BSAs, a model for the association mechanism was derived by in silico methods, some deductions about the dissociation might be inferred from the bound state, given that it necessarily represents the starting point for the dissociation mechanism [25]. For the 4-alkyl substituted BSAs, an intermediate pre-binding state governed by hydrophobic interactions is the initial and rate-limiting step in the association between hCAII and BSAs [25]. Subsequently, on the way to the final binding pose, the ligand forms hydrophobic interactions with the hydrophobic wall, but additionally engages in hydrogen bonding with Thr199, Thr200, and the Zn^II^-bound hydroxide ion [25]. Given the increased hydrophobic character that is often entailed by fluorination, a similar scenario can be envisaged for the compounds investigated herein. However, the rate-limiting step needs not necessarily be the same in this case. To assess whether hydrophobicity is important for binding kinetics, we correlated the association (*k*_on_) and dissociation rates (*k*_off_) with log*P* values from literature of the considered ligands (Figure 13) [18]. However, no clear-cut correlation is obvious.

For the elucidation of the dissociation rates, it is reasonable to assume that the re-protonation of the ligand may play a role in the rate-limiting step, given that BSAs bind as anions. In this scenario, the p*K*_a_ values should correlate with the dissociation rates (*k*_off_). However, Figure 11 definitely indicates that also here a more complex correlation is given for *k*_off_.

**Figure 14:**
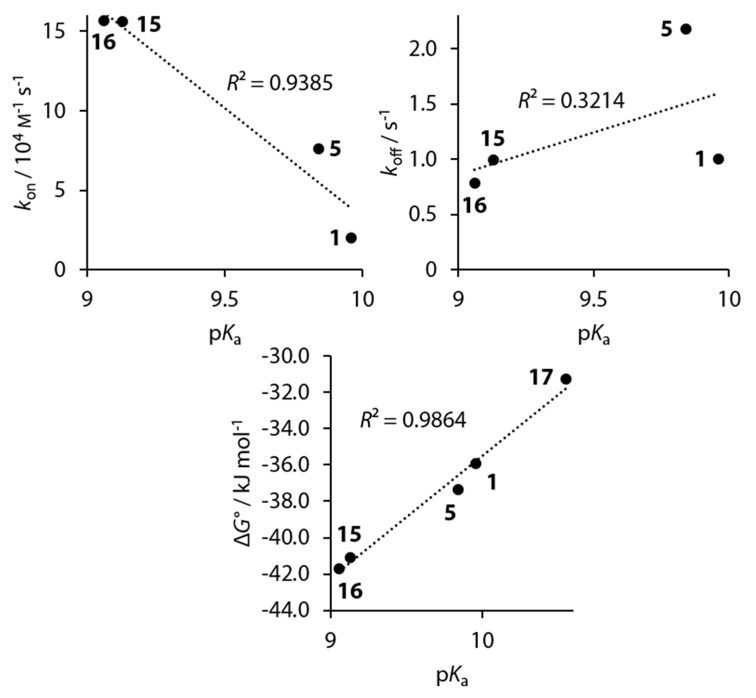
Plots of *k*_on_ and *k*_off_ of compounds **1**, **5**, **15**, and **16** and plot of Δ*G*° of compounds **1**, **5**, **15**, **16**, and **17** against the p*K*_a_ value.

To reduce complexity, we first want to analyze compounds **1**, **5**, **15**–**17**, which do not cause major structural perturbations in the active site. A correlation between the p*K*_a_ value and both Δ*G*° and *k*_on_ can be inferred, but not between p*K*_a_ and *k*_off_ values (Figure 14).

Different to the *para*-substituted derivatives, the other ligands bearing *ortho* or *meta* fluoro-substituents seem to contribute additional features that modulate the correlation of structural with biophysical data. For example, compounds **4**, **6**, **7**, and **9**, which share the common feature of a hydrophobically accommodated fluorine atom in *meta* position along with solely one major binding pose of the phenyl ring, all have comparably high association rates and simultaneously relatively low dissociation rates. With some care, this feature seems to assign a fluorine atom in this position a special role in the binding kinetics of the studied series. Furthermore, the comparison of compounds **6** and **10** reveals that, in addition to the thermodynamic similarity shown above, the kinetic signatures are almost identical. This provides further support for the assumption, that fluorination in *meta* position is, along with the accommodation of a fluorine atom in the hydrophobic pocket, favorable for the binding to hCAII. This observation is further supported by the kinetic data of the fluorinated ligands **3** and **8**, which only feature an *ortho* fluoro substituent but lack a *meta* fluorine atom that could favorably accommodate the hydrophobic niche. Both together with **5**, which lacks any *ortho* or *meta* fluoro substituent, show shorter off-rates and longer on-rates compared with the remaining fluoro derivatives (Figure 11).

## 5. Conclusions

We herein present high-resolution crystallographic and ITC data of a series of variously 4-substituted BSAs with additionally different degrees of fluorination in *ortho* and *meta* position of the central aromatic portion. The applied substitution pattern has a significant influence on the ligand’s acidity (Table 2). While a straightforward structure activity relationship is difficult to establish because of the complex and multi-featured nature of the binding of these compounds, there is evidence that a *meta* fluorine substituent is favorable for both overall affinity and also large association and small dissociation constants. Moreover, it could be shown that the incorporation of either one additional fluorine atom (compare compounds **3** and **8**) or two additional fluorine atoms (compare compounds **6** and **10**) can be futile, at least with respect to the biophysical properties.

A small set of chemically diverse, only *para*-substituted BSAs shows that an increasing acidity provides for a faster association process. As we demonstrated previously, the association between *para n*-alkyl substituted BSAs benefits from a hydrophobic contact between the ligands and hCAII prior to accommodation in the catalytic center [25]. This pre-binding at a hydrophobic patch on the surface of the protein, next to the entrance into the funnel-shaped active site, has proved decisive as rate-determining step for the *n*-alkyl series of ligands. However, it can be expected that the alkyl series features highly similar acidities. In combination with the findings presented herein, it can be reasoned, that, as long as acidity is maintained for structurally similar *para*-substituted BSAs, the formation of this hydrophobic contact represents the rate-limiting step. However, if acidity is strongly modulated while structural similarity is largely preserved, supposedly the transfer of one of the sulfonamide protons to the Zn^II^-bound hydroxide ion prior to active-site binding becomes the rate-limiting step. This finding is clearly indicated by the 4-substituted BSAs of deviating acidity (Figure 14). This correlation still seems to hold for the entire series of studied ligands (Figure 11) considering that ligands such as **3** or **8** solely bear an unfavorably positioned *ortho* fluoro substituent feature slower on-rates and faster off-rates, a phenomenon that is difficult to explain structurally or electronically on the basis of available material. Likely, a series of *ortho* modulated ligands needs to be studied. The correlation of structural features with *k*_off_ is more challenging. In our previous study on narrow series of 4-alkyl and 4-alkoxy BSAs, the snug fit of ligand and protein appeared to be an important criterion for longer off-rates, likely due to impeded re-hydration of the site [20,48]. In the present series, the geometrical variability along with conformational adaptations of Thr200 make the picture more complex, however, an optimal placement of the *meta* fluoro-substituent in the hydrophobic niche of the protein seems to support extended off-rates. Elaborate computer simulations will definitely be required to validate our hypothesis based on experimental evidence. 

Finally, the application of the novel method of kinITC calls for a comparison with an established method such as surface plasmon resonance (SPR). So far, only a comparably small amount of kinetic data from ITC measurement is available. A part of the molecules investigated herein are valuable for the further development of the method, as there are already thermodynamic data for these. We could show that our novel measurement protocol applied previously [20] delivered similar affinities and similar enthalpic trends for these molecules [15,18]. Unfortunately, no kinetic data from SPR measurements are available for these molecules at this point. While it was shown by Zihlmann et al. that the difference between both methods can be as small as a factor of two, we found in our previous study that it is possible that on-rate and off-rate constants differ by a factor of ten while both methods reveal similar affinities [20,49]. It is reasonable to assume that differences can arise simply due to the fact that ITC is an equilibrium-based method while SPR operates under steady-state conditions. Furthermore, in ITC, the molecules are floating in solution without motional restrictions, while in SPR one species is fixed on a matrix. Given that the protein is immobilized, the accessibility of the ligand-binding site depends on the protein residue that is covalently linked to the matrix. If it is close to the binding site’s entrance, the binding site will be less accessible than in the case of a coupled residue that is farther away. Clearly, more experimental evidence is necessary to elucidate the differences between both methods and to receive a clear understanding of how different molecular properties bear on the kinetic results from either method.

## Figures and Tables

**Table 1:** Compounds investigated herein with geometric metrics derived from the respective crystallographic models. Derivations of α, *d* (the absolute value of the deviation vector $\vec{d}$ and the torsion angle are depicted in Figure 1.

#	Fluorinated Positions	R	PDB Entry	Occupancy*^b^*	α / °*^c^*	Torsion Angle *τ* / °*^d^*	*d* / Å	RMSD Thr200*^e^*	Interface area F/protein *meta* / Å^2*f*^	Interface Area F/protein *ortho* / Å^2*g*^
**1**	-	H	6GDC*^a^*	1.0	0.9	49.2	3.5	-		
**2**	-	Me	6GM9*^a^*	1.0	2.4	50.9	3.5	0		
**3**	2	H	6RIT	1.0	5.0	55.7	3.6	0.183		
**4**	3	H	6RQI	1.0	2.9	49.1	3.6	0.072	127.4	
**5**	4	F	6RKN	1.0	1.6	50.2	3.5	0.022		
**6**	2,3	H	6RJJ	1.0	40.1	0.5	4.0	0.086, 0.221	161.1	140.3
**7**	2,5	H	6RNP	1.0	4.7	54.8	3.5	0.205	114.34	
**8**	2,6	H	6ROE	0.66, 0.34	4.7	2.1, 56.9	3.6	0.061, 0.272		138.8
**9**	3,5	H	6RRG	0.69, 0.31	2.0	2.3, 54.7	3.6	0.07	157.3	
**10**	2,3,5,6	H	6RRI	1.0	42.2	2.0	4.0	0.077	142.0	138.3
**11**	2,3,5,6	Me	6RS5	0.35, 0.65	5.3	0.1, 58.2	3.7	0.068, 0.298	157.4	137.0
**12**	2,3,5,6	Et	6RSZ	1.0	42.0	1.2	4.0	0.065	165.6	139.9
**13**	2,3,5,6	Pr	6S9G	1.0	41.2	2.1	4.0	0.097	159.9	138.9
**14**	2,3,4,5,6	F	6SD7	1.0	41.8	4.2	4.1	0.059	155.7	138.5
**15**	-	CN	6ROB	1.0	2.4	48.8	3.6	0.039	-	-
**16**	-	NO_2_	6RH4	1.0	3.2	49.2	3.6	0.038	-	-
**17**	-	NH_2_	6RL9	1.0	2.2	50.1	3.6	0.015	-	-

*^a^* Previously published in [20]. *^b^* If two values are given, the first refers to the reference conformation, the second to the alternative conformation. *^c^* Measured for the reference conformation of Leu198 using PyMOL the NumPy library for vector construction and the vg library for vector operations [21,22,23]. If two ligand conformations exist, the value refers to the alternative conformation, as this conformation is always correlated with the reference conformation of Leu198 in these cases. *^d^* The *ortho* carbon atom closer to Thr200 was used. *^e^* Measured using the function rms from PyMOL including all atoms of Thr200 except riding hydrogen atoms relative to the hCAII–1 complex (6GDC). *^f^* Determined with the program dr_sasa in mode 4, using the fluorine atom accommodated in the hydrophobic pocket formed by the side chains of residues Val121, Phe131, Leu141, and Leu198 as ligand and only side chain atoms of these residues as receptor [24]. Calculated only for the reference orientation of Leu198 and the respective binding mode of the ligand. *^g^* Determined with the program dr_sasa for Linux in mode 4, using the fluorine atom accommodated in the hydrophobic pocket formed by the side chains of residues Val121, Leu141, Val143, and Leu198 as ligand and only side chain atoms of these residues as receptor [24]. Calculated only for the reference orientation of Leu198 and the respective binding mode of the ligand.

**Table 2:** p*K*_a_ values of compounds investigated herein.

Compound	p*K*_a_
**1**	9.96 ± 0.01
**2**	10.15 ± 0.01
**3**	9.38 ± 0.01
**4**	9.57 ± 0.01
**5**	9.84 ± 0.01
**6**	8.98 ± 0.01
**7**	8.99 ± 0.01
**8**	8.90 ± 0.01
**9**	9.18 ± 0.01
**10**	8.08 ± 0.01
**11** *^a^*	8.20 ± 0.01
**15**	9.13 ± 0.01
**16**	9.06 ± 0.01
**17**	2.12 ± 0.01, 10.55 ± 0.01

*^a^* Compounds **12** and **13** were not taken into account due to the chemical similarity with compound **11**.

## Supplementary Materials

The following are available online at https://www.mdpi.com/2218-273X/10/4/509/s1, Figure S1: Plot of the observed standard enthalpy of binding as function of the buffer ionization heat. Plots of thermograms, isotherms and equilibration-time curves. Figure S2: Injection signals for the determination of the response time. Syntheses of compounds **6** and **10**–**13**, Table S1: Crystallographic data for compounds **3**–**17**. Table S2: Thermodynamic data for compounds **3**–**13**. Table S3: Thermodynamic data for the interaction of compound **1** with hCAII. Table S4: Kinetic data for compounds **1**–**10**.
