# Supplementary material for: The Influence of Varying Fluorination Patterns on the Thermodynamics and Kinetics of Benzenesulfonamide Binding to Human Carbonic Anhydrase II
Source: Biomolecules. 2020 Mar 27;10(4):509. doi: 10.3390/biom10040509 (PMC7226267; doi:10.3390/biom10040509)
Supplement: Supplementary file 1 [file biomolecules-10-00509-s001.pdf]

Supplementary Materials

The Influence of Varying Fluorination Patterns on the Thermodynamics and Kinetics of Benzenesulfonamide Binding to human Carbonic Anhydrase II

Steffen Glöckner[1], Khang Ngo[1], Björn Wagner[2], Andreas Heine[1] and Gerhard Klebe[1,*]

[1] Institut für Pharmazeutische Chemie, Philipps-Universität Marburg, Marbacher Weg 6, 35032 Marburg, Germany

[2] F. Hoffmann-La Roche AG, Pharmaceutical Research & Early Development, Roche Innovation Center Basel, 4070 Basel, Switzerland

Table of Contents

Table S1	Crystallographic data	2
Table S2	Thermodynamic data	5
Table S3	Protonation dependence	6
Figure S1	Protonation dependence	6
Figure S2	Instrument response time	7
Table S4	Kinetic data	8
ITC plots		9
Synthesis		46
Determination of purity		49
References		50

Crystallographic data

Table S1: Crystallographic data for compounds **3 – 17**.[a]

	hCAII-**3** (6RIT)	hCAII-**4** (6RQI)	hCAII-**5** (6RKN)	hCAII-**6** (6RJJ)	hCAII-**7** (6RNP)
**Data collection and processing**					
Beamline	14.1	14.1	14.1	14.1	14.1
Wavelength / Å	0.9184	0.9184	0.9184	0.9184	0.9184
Space group	$P2_1$	$P2_1$	$P2_1$	$P2_1$	$P2_1$
a,*b*,*c* / Å	42.5, 41.6, 72.5	42.4, 41.5, 72.3	42.3, 41.6, 72.2	42.4, 41.7, 72.6	42.3, 41.5, 72.3
β / °	104.4	104.6	104.6	104.6	104.5
Matthews coefficient / $Å^3$ Da^{-1} [b]	2.1	2.1	2.1	2.1	2.1
Solvent content / % [b]	41.1	40.5	40.4	41.1	40.4
**Diffraction data**					
Resolution range / Å	41.6 – 1.00 (1.07 – 1.01)	41.5 – 0.95 (1.01 – 0.95)	41.6 – 0.96 (1.02 – 0.96)	41.7 – 1.06 (1.12 – 1.06)	41.5 – 1.07 (1.13 – 1.07)
Unique reflections / Å	126668 (19712)	146227 (22470)	139058 (21525)	109461 (17106)	104839 (16391)
$CC_{1/2}$ / %[1]	99.9 (89.1)	99.8 (83.3)	99.8 (93.4)	99.9 (84.2)	99.8 (83.1)
R_{sym} / %[2]	4.9 (39.4)	5.1 (39.3)	5.3 (23.9)	5.1 (53.0)	6.0 (47.7)
Completeness	97.5 (94.3)	95.5 (91.1)	93.8 (90.2)	97.3 (94.3)	97.9 (95.2)
Wilson B factor / $Å^2$	6.5	8.1	7.5	8.6	8.3
Multiplicity	3.6 (3.4)	3.6 (3.3)	3.7 (3.5)	3.7 (3.6)	3.8 (3.6)
$I / \sigma(I)$	13.7 (2.46)	10.7 (2.2)	13.3 (3.9)	12.5 (2.0)	10.9 (2.1)
**Refinement**					
Resolution range / Å	40.1 – 1.01	41.0 – 0.95	41.0 – 0.96	41.0 – 1.06	41.0 – 1.07
Reflections used in refinement (work/free)[3] [c]	126658 (120324/6334)	146215 (138903/7312)	139048 (132096/9652)	109451 (103978/5473)	104826 (99584/5242)
Final *R* values for all reflections (work/free)[3][c]	0.125/0.140	0.116/0.130	0.1084/0.1242	0.123/0.138	0.119/0.136
Protein residues	257	257	257	257	257
Inhibitor atoms	11/11	11	11/11	12/12	12/12
Water molecules	228	206	248	263	272
RMSD from ideality					
Bond lengths / Å	0.009	0.006	0.007	0.009	0.007
Bond angles / °	0.91	0.99	1.07	1.11	1.02
Ramachandran plot / % [d]					
Residues in most favored regions	90.7	89.8	90.3	89.8	88.9
Residues in additionally allowed regions	8.8	10.2	9.3	9.7	11.1
Regions in generously allowed regions	0.5	0	0.5	0.5	0
Residues in disallowed regions	0	0	0	0	0
Mean *B* factor / $Å^2$ [e]					
Protein non-hydrogen atoms	9.3	10.5	9.4	11.6	11.6
Inhibitor	7.5	8.5	8.1	9.9	8.6
Water molecules	20.2	22.3	21.7	23.7	23.7

[a] Data in parentheses refer to the highest resolution shell unless stated otherwise. Calculated using the program *Phaser Cell Content Analysis* from the *CCP4* suite [4]. [c] 5 % of all reflections were used for R_{free} calculation. [d] Calculated using the program *PROCHECK* [5]. [e] Calculated using the program MOLEMAN [6].

Table S1 continued.[a]

	hCAII-**8** (6ROE)	hCAII-**9** (6RRG)	hCAII-**10** (6RRI)	hCAII-**11** (6RS5)	hCAII-**12** (6RSZ)
**Data collection and processing**					
Beamline	14.1	14.1	14.2	14.2	14.2
Wavelength / Å	0.9184	0.9184	0.9184	0.9184	0.9184
Space group	$P2_1$	$P2_1$	$P2_1$	$P2_1$	$P2_1$
a,*b*,*c* / Å	42.4, 41.4, 72.3	42.6, 41.7, 72.7	42.3, 41.4, 72.3	42.4, 41.5, 72.3	42.2, 41.4, 72.1
β / °	104.5	104.5	104.5	104.6	104.5
Matthews coefficient / $Å^3\ Da^{-1}$ [b]	2.1	2.1	2.1	2.1	2.1
Solvent content / % [b]	40.3	41.4	40.2	40.5	40.0
**Diffraction data**					
Resolution range / Å	41.4 – 0.94 (1.00 – 0.94)	41.7 – 1.13 (1.20 – 1.13)	41.4 – 1.10 (1.16 – 1.10)	41.5 – 1.07 (1.13 – 1.07)	41.5 – 1.09 (1.16 – 1.09)
Unique reflections / Å	152196 (23134)	91331 (14083)	97931 (15202)	103648 (16636)	96745 (14752)
$CC_{1/2}$ / %[1]	99.9 (95.4)	99.8 (83.9)	98.8 (95.4)	99.8 (91.7)	99.9 /99.0)
R_{sym} / %[2]	4.3 (18.7)	5.8 (49.2)	5.0 (43.5)	4.1 (23.4)	3.5 (9.8)
Completeness	96.5 (91.5)	97.9 (94.1)	99.9 (82.1)	96.5 (96.5)	95.9 (91.0)
Wilson B factor / $Å^2$	7.3	9.6	9.9	9.6	8.9
Multiplicity	3.5 (3.0)	3.7 (3.5)	3.6 (3.6)	2.5 (2.4)	3.7 (3.8)
I / $\sigma(I)$	14.8 (3.6)	11.3 (2.0)	12.3 (2.5)	11.8 (2.9)	20.9 (9.3)
**Refinement**					
Resolution range / Å	35.6 – 0.94	41.2 – 1.13	22.9 – 1.10	23.8 – 1.07	23.3 – 1.09
Reflections used in refinement (work/free)[3] [c]	152179 (144569/7610)	91307 (86740/4567)	97922 (93026/4896)	103624 (98443/5181)	96732 (91895/4837)
Final *R* values for all reflections (work/free)[3][c]	0.110/0.119	0.127/0.140	0.119/0.139	0.118/0.132	0.113/0.129
Protein residues	257	257	257	257	257
Inhibitor atoms	12/12/12	12/12	14/14	15/15/15	16
Water molecules	209	251	247	230	261
RMSD from ideality					
Bond lengths / Å	0.006	0007	0.006	0.007	0.007
Bond angles / °	0.97	0.97	0.97	0.998	0.98
Ramachandran plot / % [d]					
Residues in most favored regions	88.9	88.0	89.4	89.8	89.8
Residues in additionally allowed regions	10.6	11.6	10.2	10.2	9.7
Regions in generously allowed regions	0.5	0.5	0.5	0	0.5
Residues in disallowed regions	0	0	0	0	0
Mean *B* factor / $Å^2$ [e]					
Protein non-hydrogen atoms	9.35	12.8	12.5	11.6	12.2
Inhibitor	7.6/8.7	9.3/9.8	11.9/18.1	9.3/10/19.9	11.6
Water molecules	20.0	24.5	25.4	22.3	24.3

[a] Data in parentheses refer to the highest resolution shell unless stated otherwise. Calculated using the program *Phaser Cell Content Analysis* from the *CCP4* suite [4]. [c] 5 % of all reflections were used for R_{free} calculation. [d] Calculated using the program *PROCHECK* [5]. [e] Calculated using the program MOLEMAN [6].

Table S1 continued.[a]

	hCAII-**13** (6S9G)	hCAII-**14** (6SD7)	hCAII-**15** (6ROB)	hCAII-**16** (6RH4)	hCAII-**17** (6RL9)
**Data collection and processing**					
Beamline	14.2	14.2	14.1	14.1	14.1
Wavelength / Å	0.9184	0.9184	0.9184	0.9184	0.9184
Space group	$P2_1$	$P2_1$	$P2_1$	$P2_1$	$P2_1$
a,*b*,*c* / Å	42.3, 41.5, 72.1	42.3, 41.4, 72.3	42.3, 41.5, 72.2	42.4, 41.5, 72.3	42.5, 41.5, 72.0
β / °	104.5	104.7	104.5	104.7	104.7
Matthews coefficient / $Å^3\ Da^{-1}$ [b]	2.1	2.1	2.1	2.1	2.1
Solvent content / % [b]	40.3	40.1	40.3	40.6	40.4
**Diffraction data**					
Resolution range / Å	41.5 – 1.14 (1.21 – 1.14)	41.4 – 1.05 (1.11 – 1.05)	41.5 – 0.93 (0.99 – 0.93)	41.5 – 0.95 (0.95 – 0.95)	41.0 – 1.00 (1.06 – 1.00)
Unique reflections / Å	87774 (13942)	111421 (17610)	155258 (22819)	148154 (22694)	130686 (20986)
$CC_{1/2}$ / %[1]	99.9 (80.2)	99.9 (91.3)	99.9 (97.2)	99.8 (90.9)	99.7 (82.5)
R_{sym} / %[2]	5.3 (47.1)	4.5 (25.5)	4.0 (13.6)	5.3 (28.0)	6.6 (43.3)
Completeness	98.8 (97.5)	98.6 (96.6)	95.4 (86.9)	96.3 (91.5)	99.6 (99.2)
Wilson B factor / $Å^2$	11.2	9.8	7.4	6.8	8.3
Multiplicity	3.3 (3.6)	3.5 (3.1)	3.5 (2.9)	3.5 (3.2)	3.6 (3.3)
I / σ(I)	11.2 (2.1)	13.6 (3.1)	17.0 (5.06)	12.7 (3.3)	9.5 (2.1)
**Refinement**					
Resolution range / Å	23.8 – 1.14	23.8 – 1.05	40.9 – 0.93	41.0 – 0.95	32.0 – 1.00
Reflections used in refinement (work/free)[3] [c]	87761 (83373/4388)	111410 (105840/5570)	155254 (147491/7763)	148150 (140742/7408)	130676 (124142/6534)
Final *R* values for all reflections (work/free)[3][c]	0.123/0.147	0.124/0.139	0.112/0.120	0.103/0.117	0.119/0.130
Protein residues	257	257	257	257	258
Inhibitor atoms	17/15	15/29	12/12	13/13	11/11/11
Water molecules	269	171	208	253	220
RMSD from ideality					
Bond lengths / Å	0.006	0.006	0.006	0.008	0.008
Bond angles / °	0.984	0.998	0.968	1.07	1.08
Ramachandran plot / % [d]					
Residues in most favored regions	89.4	89.4	89.8	89.4	88.9
Residues in additionally allowed regions	9.7	10.6	9.7	10.2	10.6
Regions in generously allowed regions	0.9	0	0.5	0.5	0.5
Residues in disallowed regions	0	0	0	0	0
Mean *B* factor / $Å^2$ [e]					
Protein non-hydrogen atoms	14.2	12.2	8.9	9.3	10.8
Inhibitor	12.2/27.2	13.9/14.8	8.5/13.5	9.5/16.5	8.4
Water molecules	28.8	22.0	20.0	21.7	22.9

[a] Data in parentheses refer to the highest resolution shell unless stated otherwise. Calculated using the program *Phaser Cell Content Analysis* from the *CCP4* suite [4]. [c] 5 % of all reflections were used for R_{free} calculation. [d] Calculated using the program *PROCHECK* [5]. [e] Calculated using the program MOLEMAN [6].

Thermodynamic data

Table S2: Thermodynamic data for compounds **3** – **13** and **15** – **17**.

					Error values			
**Compound**	**Δ*G*° / kJ mol^{-1}**	**Δ*H*° / kJ mol^{-1}**	**-*T*·Δ*S*° / kJ mol^{-1}**	***K*$_d$ / M**	**Δ*G*° / kJ mol^{-1}**	**Δ*H*° / kJ mol^{-1}**	**-*T*·Δ*S*° / kJ mol^{-1}**	***K*$_D$ / M**
**1**	-36.0	-39.6	3.6	5.02E-07	1.10E-01	1.91E-01	1.29E-01	2.25E-08
**2**	-38.0	-40.8	2.8	2.24E-07	3.40E-1	2.40E-1	0.51	2.85E-08
**3**	-38.2	-48.2	9.9	2.00E-07	2.87E-01	3.69E-02	3.20E-01	2.48E-08
**4**	-40.2	-47.4	7.2	9.07E-08	1.63E-01	3.95E-01	5.32E-01	5.72E-09
**5**	-37.3	-39.8	2.5	2.87E-07	1.17E-01	8.97E-01	8.42E-01	1.49E-08
**6**	-42.1	-42.7	0.6	4.17E-08	2.10E-01	4.77E-01	6.92E-01	3.49E-09
**7**	-42.8	-54.4	11.7	3.21E-08	3.28E-01	4.23E-01	7.36E-01	4.77E-09
**8**	-38.3	-42.3	4.0	1.92E-07	1.32E-01	3.88E-01	4.36E-01	1.02E-08
**9**	-40.9	-41.9	1.0	6.86E-08	1.26E-01	2.35E-01	1.31E-01	3.45E-09
**10**	-42.6	-44.2	1.6	3.42E-08	6.5E-2	1.67E-03	6.51E-02	8.99E-10
**11**	-45.5	-44.0	-1.5	1.06E-08	9.3E-2	1.20E-03	9.33E-02	3.98E-10
**12**	-48.6	-42.3	-6.3	3.10E-09	7.9E-2	1.43E-03	7.94E-02	9.93E-11
**13**	-48.1	-39.5	-8.5	3.81E-09	4.6E-2	1.79E-03	4.61E-02	7.08E-11
**15**	-41.1	-43.8	2.7	6.35E-08	4.95E-2	2.33E-01	2.30E-01	1.29E-09
**16**	-41.8	-45.2	3.39	4.83E-08	1.87E-01	1.31E-01	2.48E-01	3.67E-09
**17**	-31.3	-44.8	13.5	3.29E-06	1.56E-01	1.23	1.35	2.15E-07

Thermodynamic signatures of compounds **1** – **13** measured in 10 mM HEPES buffer at pH 7.8 with *Δ*G° in blue, *Δ*H° in green and –T*Δ*S° *in* red. The values for **1** and **2** were taken from a previous publication.[7] For **1** to **10**, globally fitted values are given. Error bars represent the standard error of measurement for these compounds. Compounds **10** to **13** were characterized by a displacement experiment with subsequent global fitting, as they were not characterizable directly due to their high affinities. For these compounds, the globally fitted values of K_d and *Δ*H° and the values of *Δ*G° and –T *Δ*S° *calculated from the former two* are given. Error bars represent the error of global fitting for K_a and *Δ*H° and the error calculated from those of the former two for –T *Δ*S°.

Elucidation of putative protonation effects

Table S3: Thermodynamic data for the interaction of compound **1** with hCAII.

					Standard error		
	**ΔH_{ion} / kJ mol^{-1}** [8]	**$\Delta G°$ / kJ mol^{-1}**	**$\Delta H°_{obs}$ / kJ mol^{-1}**	**$-T\Delta S°$ / kJ mol^{-1}**	**$\Delta G°$ / kJ mol^{-1}**	**$\Delta H°_{obs}$ / kJ mol^{-1}**	**$-T\Delta S°$ / kJ mol^{-1}**
HEPES	21.07	-35.8638533	-41.9264693	-6.06819467	0.0483328	0.25221497	0.27973759
TRICINE	31.97	-35.676968	-43.5275467	-7.853368	0.37203109	0.83691621	0.96021661
TRIS	48.07	-35.7257813	-45.06168	-9.338688	0.19898331	0.2147062	0.35877648

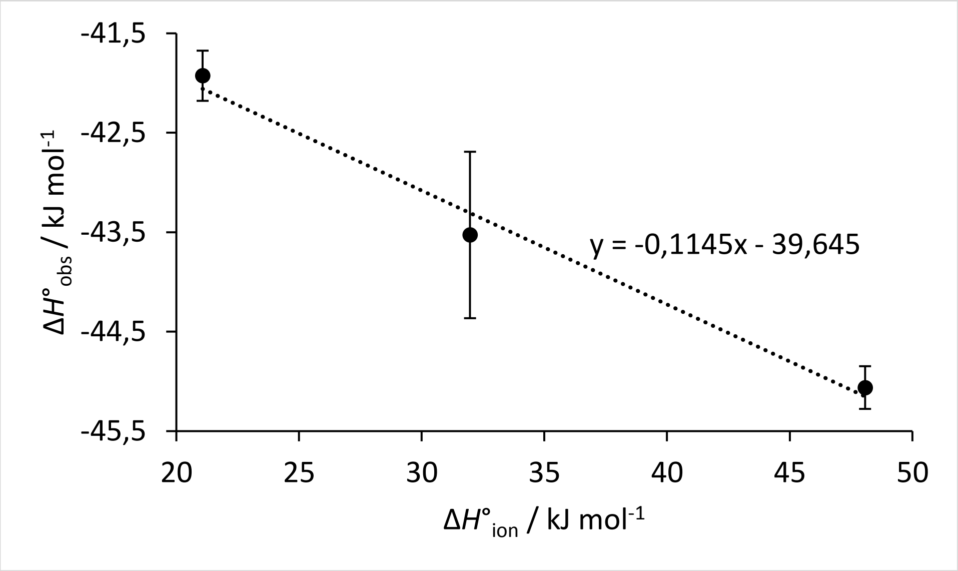

Figure S1: Plot of the observed standard enthalpy of binding as function of the buffer ionization heat.

Determination of the instrument response time

For the determination of our instrument's response time, a procedure from reference [9] was followed. A dilution titration of an aqueous solution of ethanol (p.a., 1 % v/v neglecting volume contraction) into water was performed in triplicate. An injection of 0.3 µL preceded a second titration of 2.0 µL. The resulting second injections were plotted in QtiPlot [10] and function (1) was fitted.

$$\boldsymbol{P}_{\mathrm{m}}(\boldsymbol{t}) = \boldsymbol{P}_{\boldsymbol{max}} \cdot e^{\frac{-t}{\tau_{ITC}}} + \boldsymbol{C} \qquad (1)$$

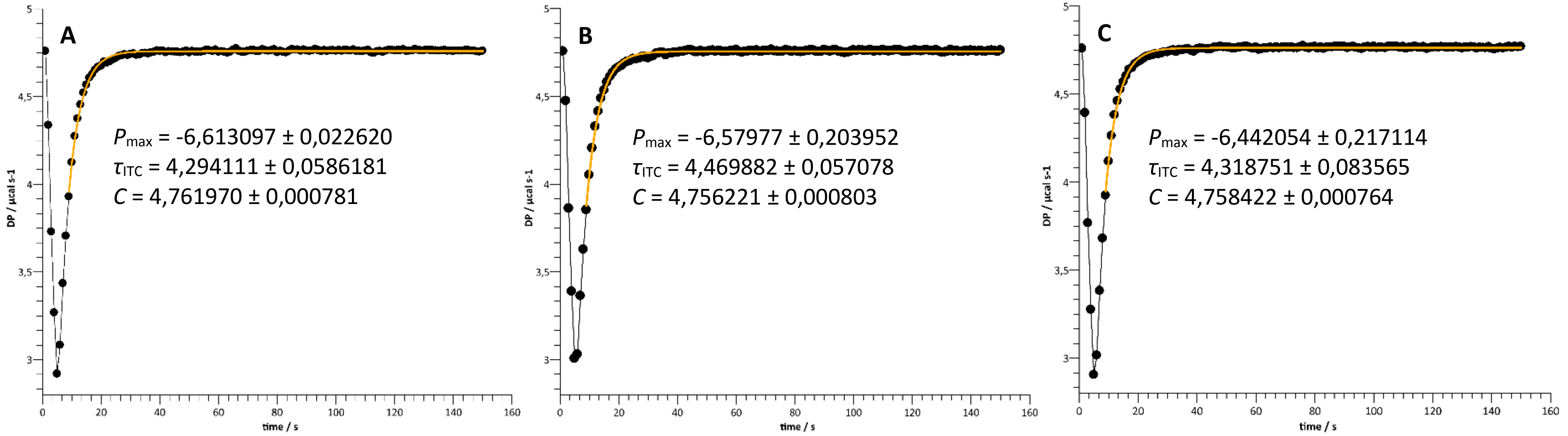

Figure S2: Plot of injection signal resulting from the titration of an aqueous solution of ethanol in water with fit parameters from equation (1) and fit error values from QtiPlot. Black dots represent the actually measured power values. Dots are connected with a black line for clarity. The orange line represents the fit function according to the given fit parameters.

Kinetic data

Table S4: Kinetic data for compounds **1** – **10**, **15** and **16**.

				Standard error for three measurements		
**Compound**	k_{on} / M^{-1} s^{-1}	k_{off} / s^{-1}	**Response time / s**	k_{on} / M^{-1} s^{-1}	k_{off} / s^{-1}	**Response time / s**
**1**[a]	2.00E+04	1.00E-02	3.5	1.56E+03	7.84E-04	1.49E-01
**2**[a]	24453.33	5.48E-03	3.3	2.87E+03	6.44E-04	3.13E-01
**3**	7.36E+04	1.47E-02	4.1	3.70E+03	7.37E-04	3.14E-02
**4**	1.11E+05	1.00E-02	4.5	1.70E+03	1.54E-04	6.11E-02
**5**	7.59E+04	2.18E-02	4.5	2.74E+03	7.83E-04	6.90E-02
**6**	1.75E+05	7.30E-03	4.4	8.06E+03	3.35E-04	2.30E-01
**7**	1.52E+05	4.89E-03	4.1	1.31E+04	4.21E-04	1.82E-01
**8**	8.97E+04	1.72E-02	4.4	3.94E+03	7.57E-04	8.27E-02
**9**	1.46E+05	1.00E-02	4.1	4.86E+03	3.34E-04	2.01E-01
**10**	1.74E+05	5.96E-03	4.6	4.21E+03	1.44E-04	5.44E-02
**15**	1.56E+05	9.91E-03	4.7	2.13E+03	1.37E-04	4.10E-02
**16**	1.57E+05	7.78E-03	4.8	2.37E+03	1.17E-04	9.42E-03

[a] Taken from reference [7].

ITC Plots: Raw and Processed Thermograms, Isotherms, Equilibration-Time Curves and Global Fitting Plots

For directly titrated compounds, raw and processed thermograms as well as isotherms and equilibration time curves before global fitting are given, as well as the globally fitted isotherms. Additionally, isotherms and equilibration time curves after adjustment with globally fitted parameters are given. For compound characterized with a displacement setup (**11** – **13**) raw and processed thermograms as well as the globally fitted isotherms are provided. Raw and processed thermograms of reference compound 4CBS are given and the results of the global fitting of the reference data are given with data for the respective compound to be characterized.

Compound 3

<u>Before global fitting</u>

Experiment 1

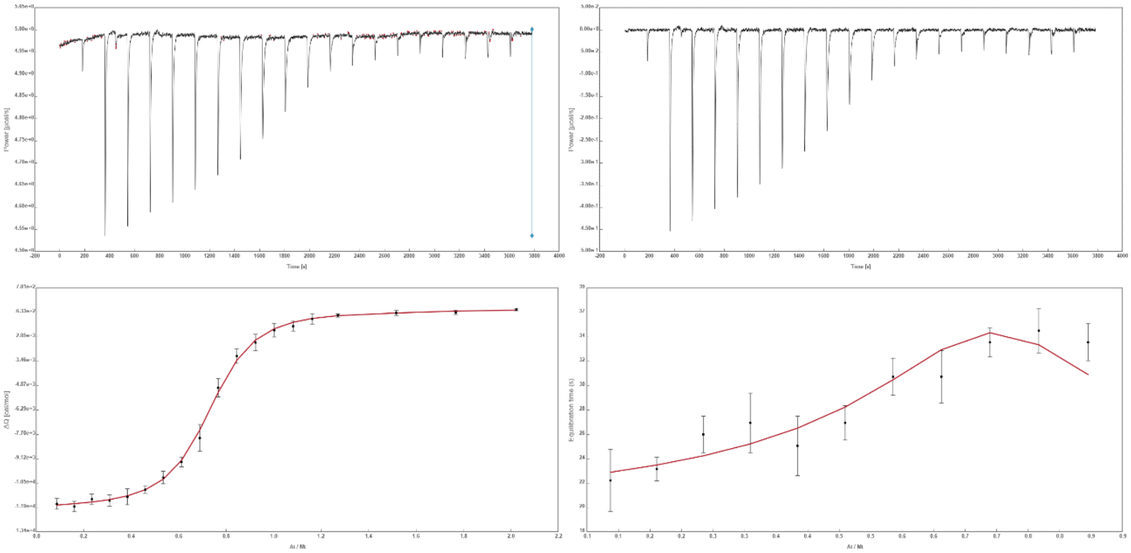

Experiment 2

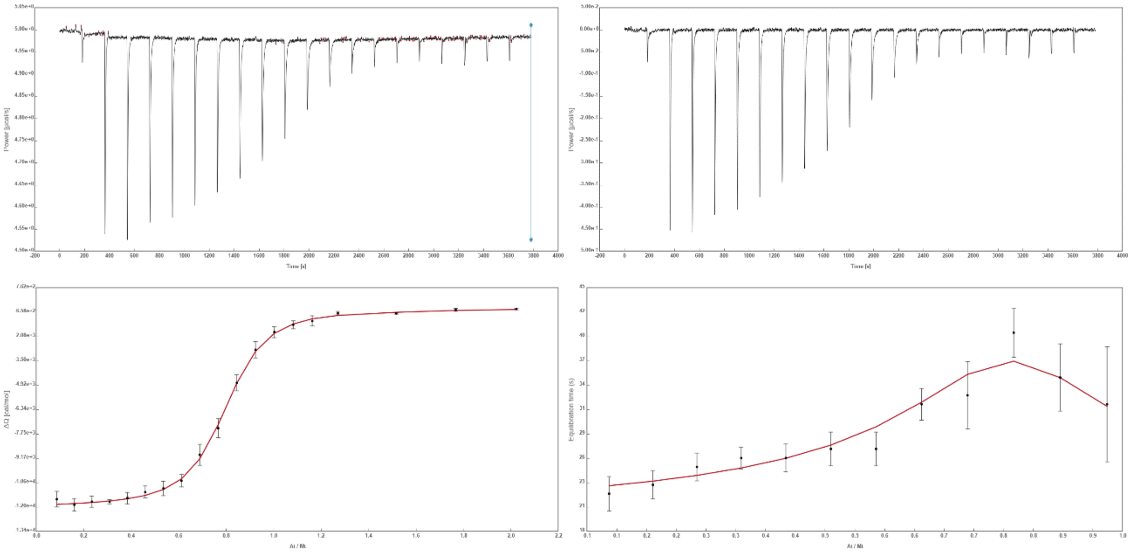

Experiment 3

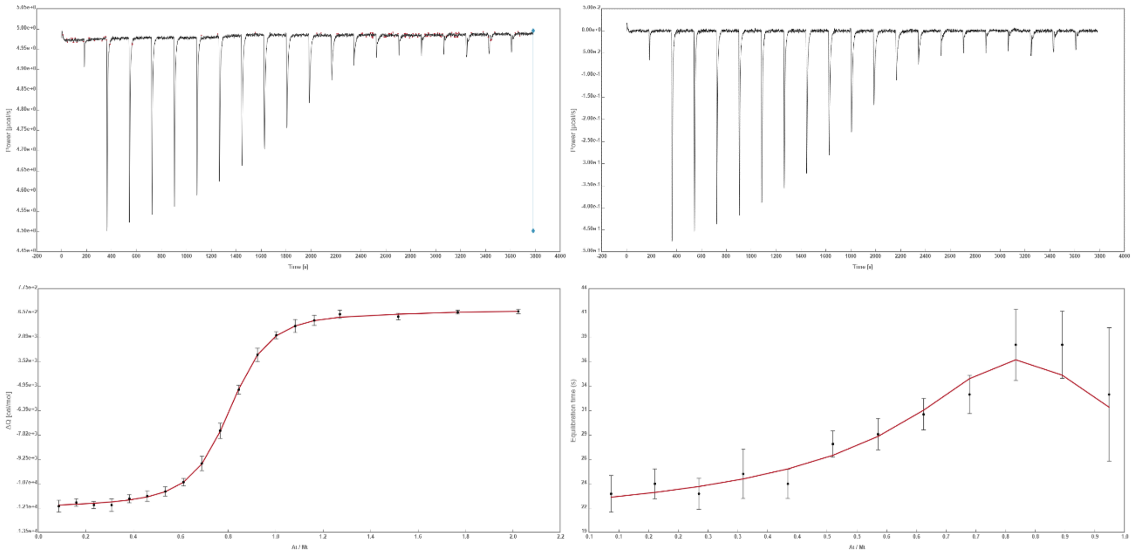

Global fitting

Experiment 1

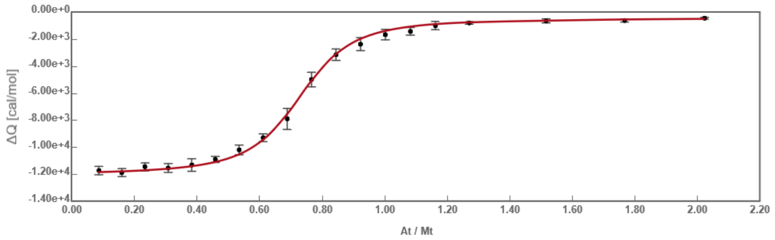

Experiment 2

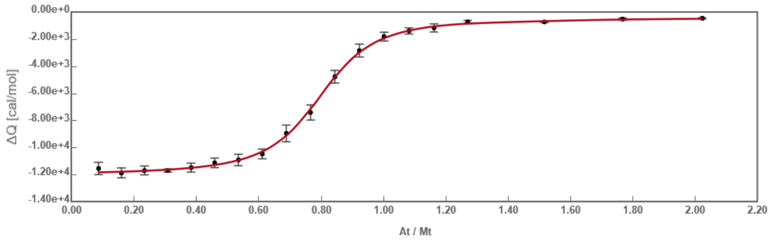

Experiment 3

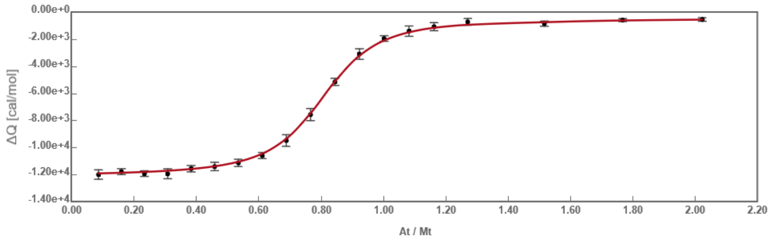

After global fitting

Experiment 1

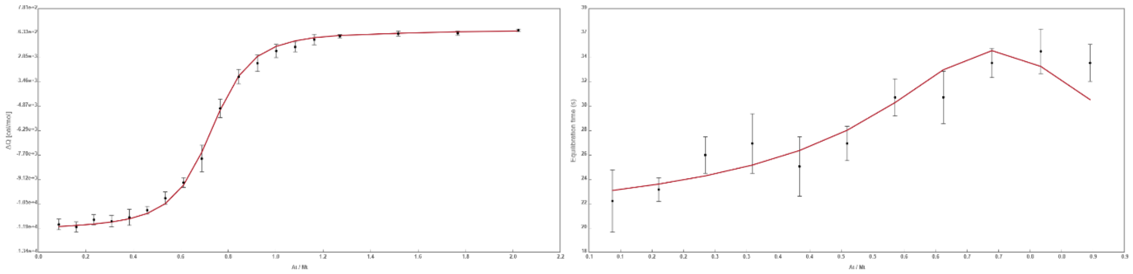

Experiment 2

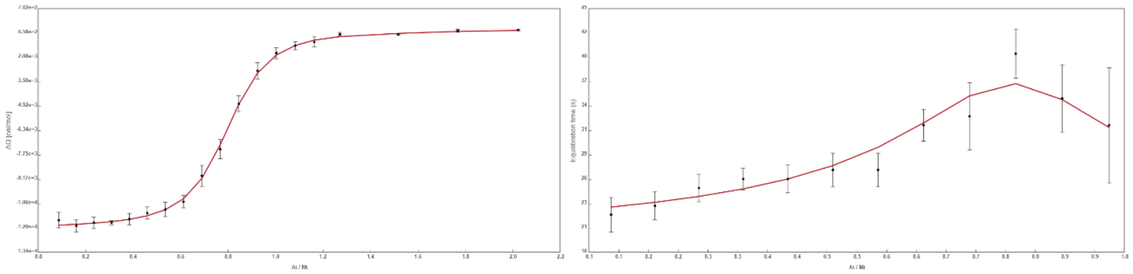

Experiment 3

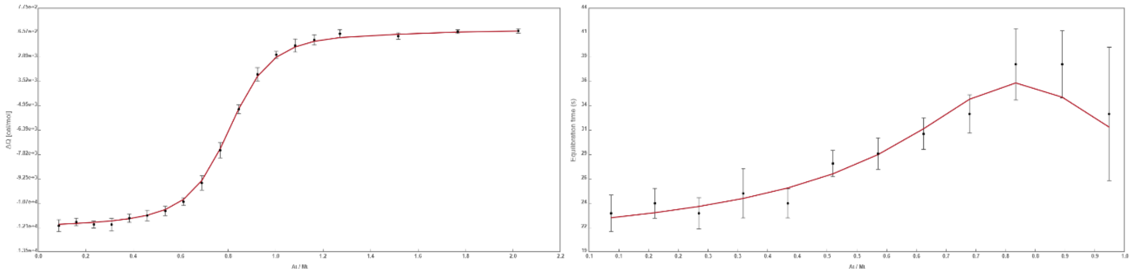

Compound 4

Before global fitting

Experiment 1

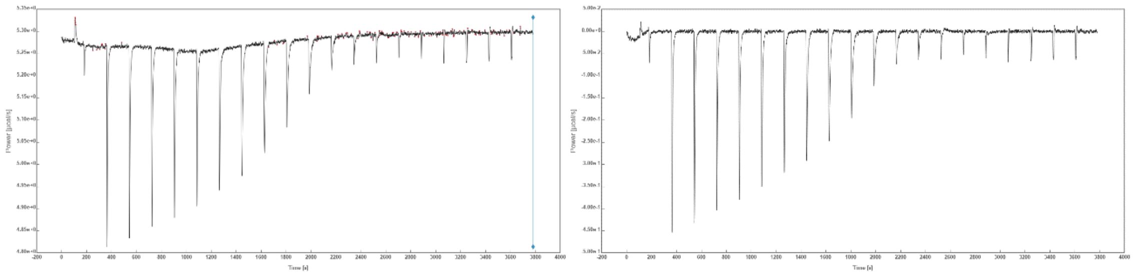

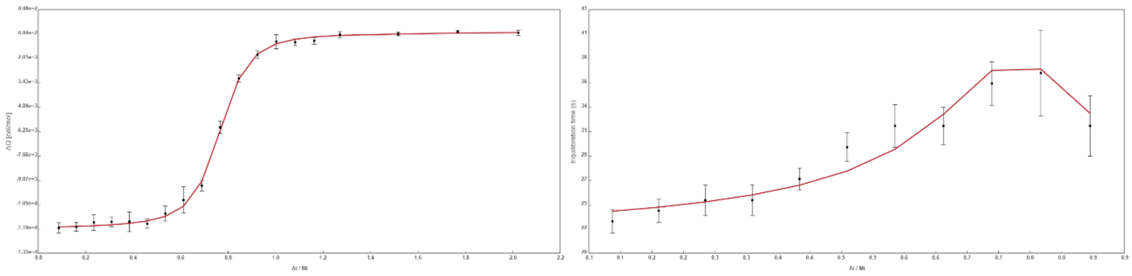

Experiment 2

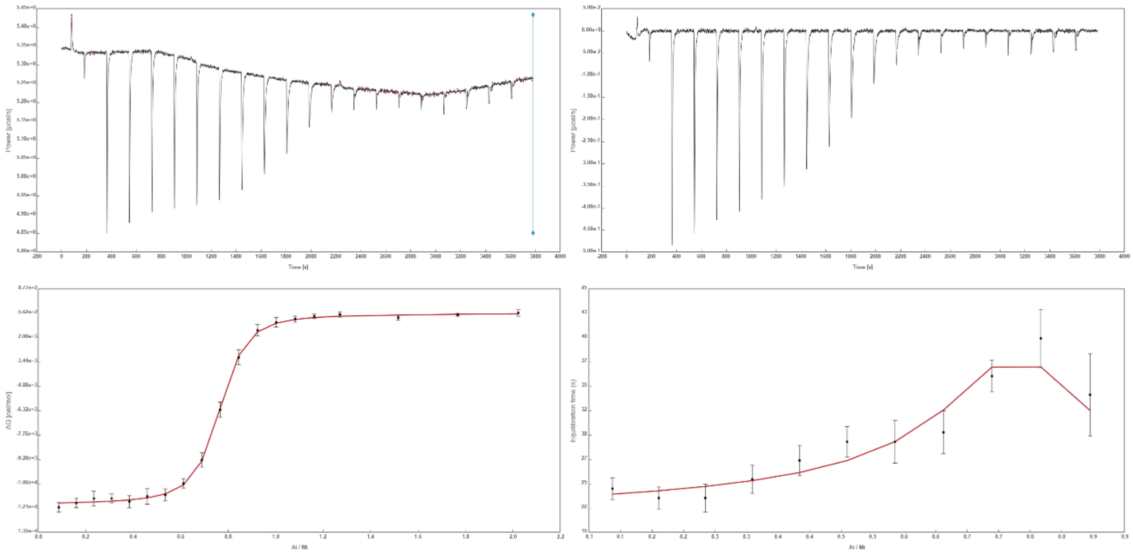

Experiment 3

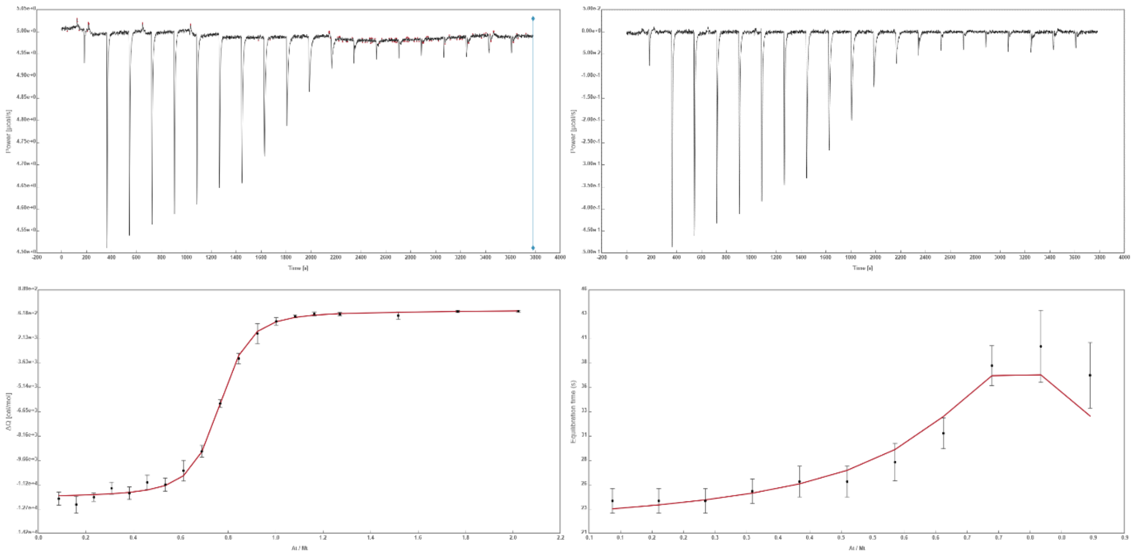

<u>Global fitting</u>

Experiment 1

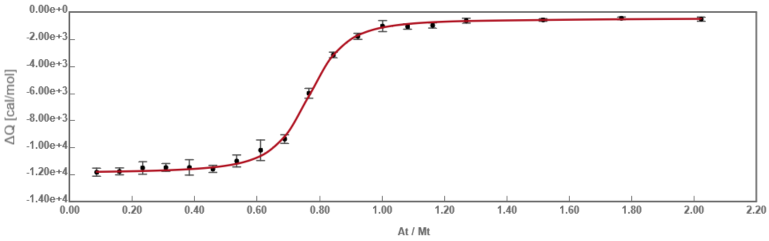

Experiment 2

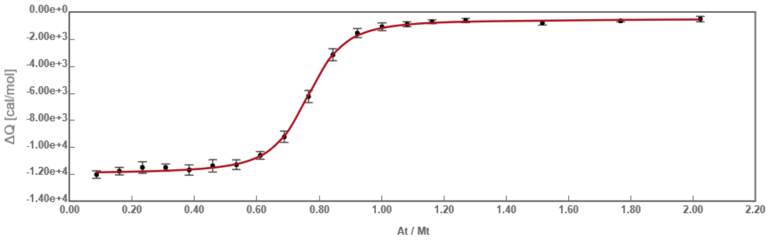

Experiment 3

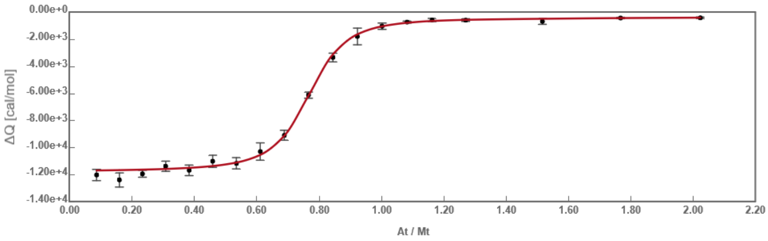

<u>After global fitting</u>

Experiment 1

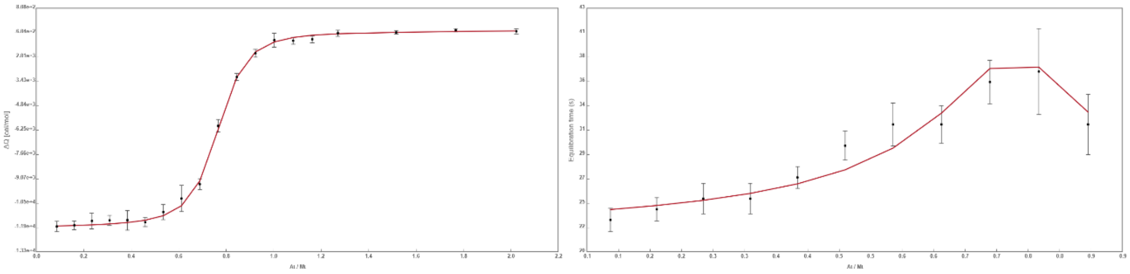

Experiment 2

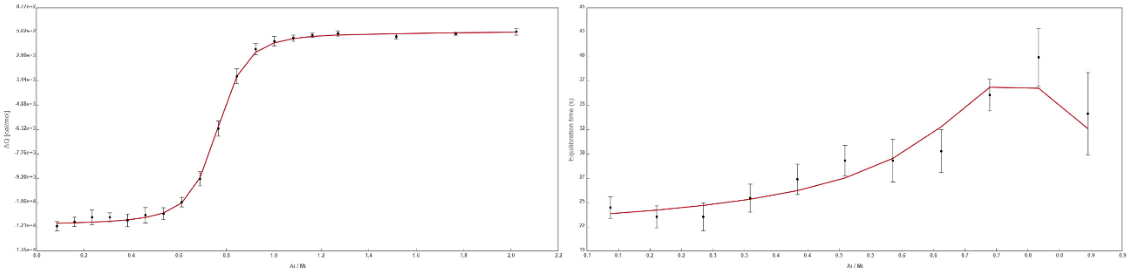

Experiment 3

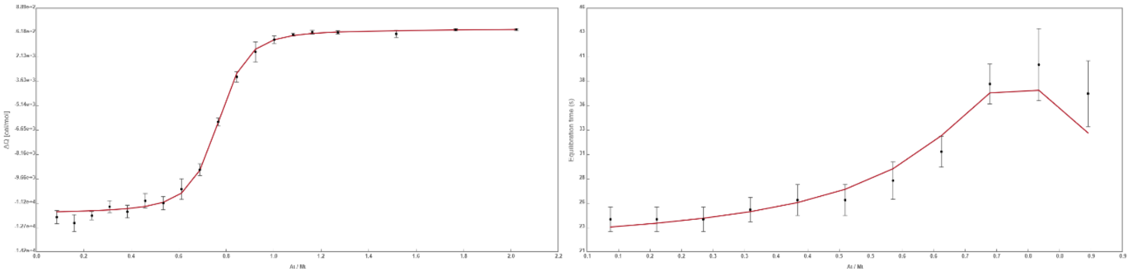

Compound 5

Before global fitting

Experiment 1

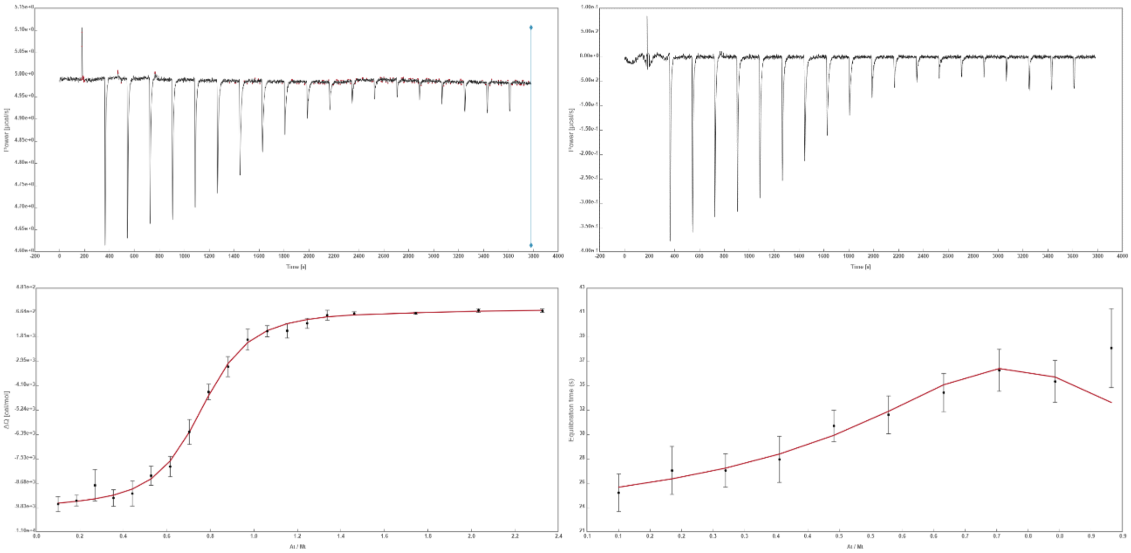

Experiment 2

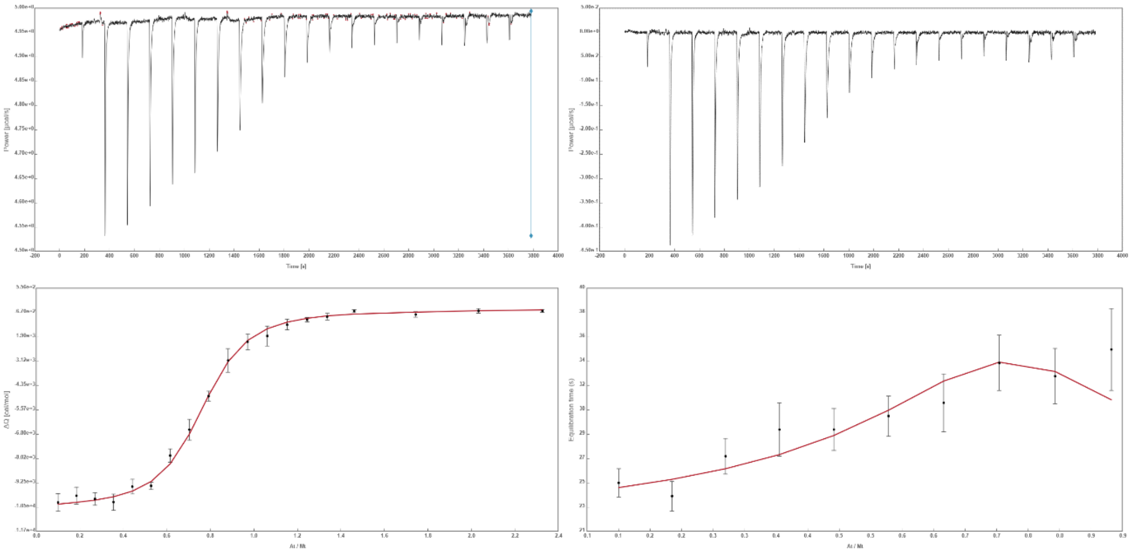

Experiment 3

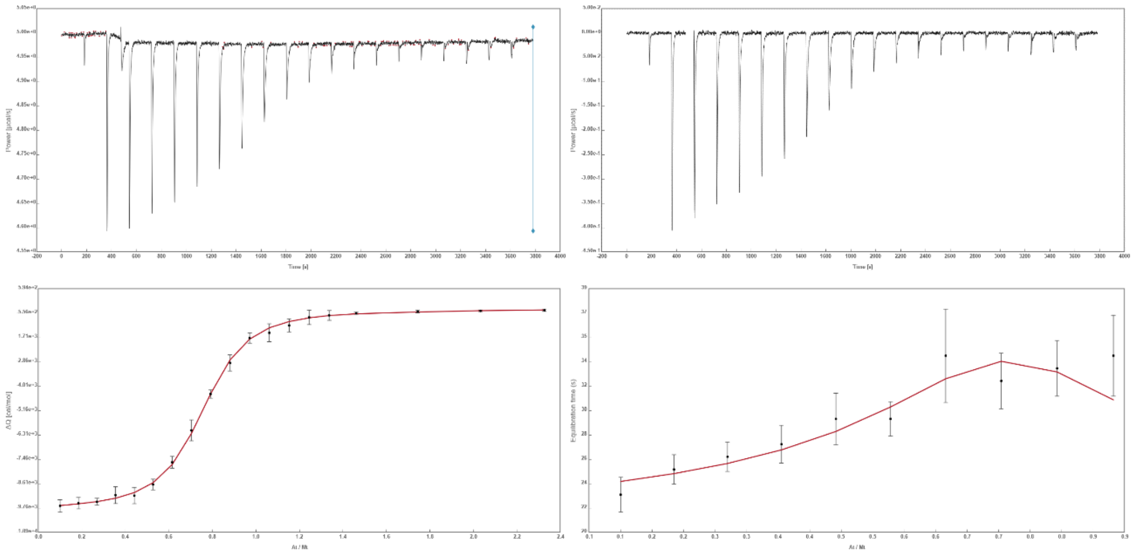

<u>Global fitting</u>

Experiment 1

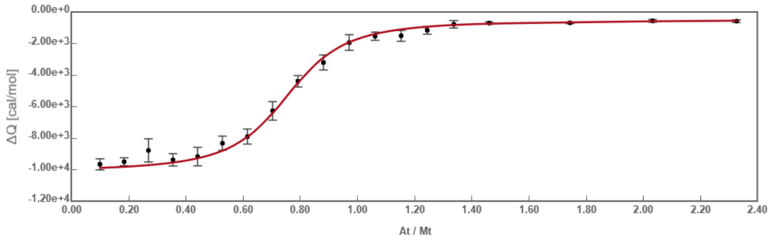

Experiment 2

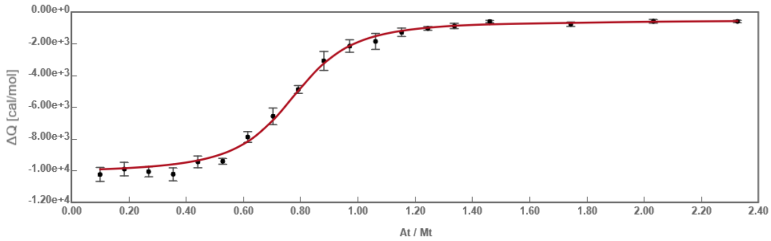

Experiment 3

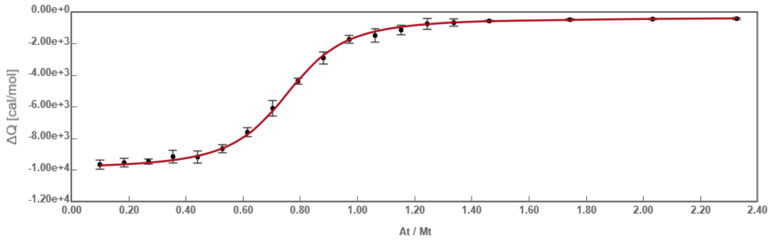

After global fitting

Experiment 1

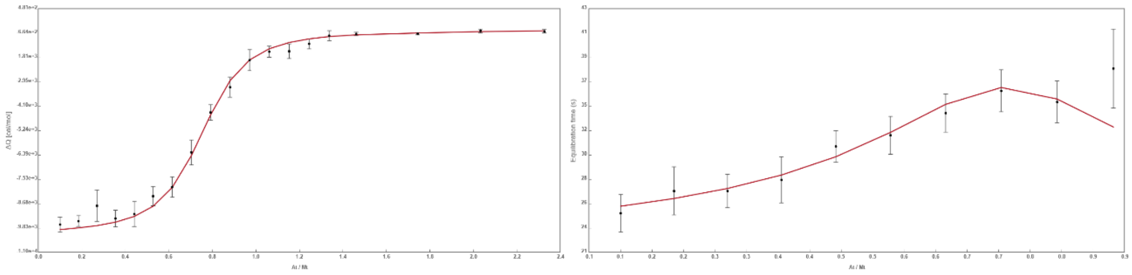

Experiment 2

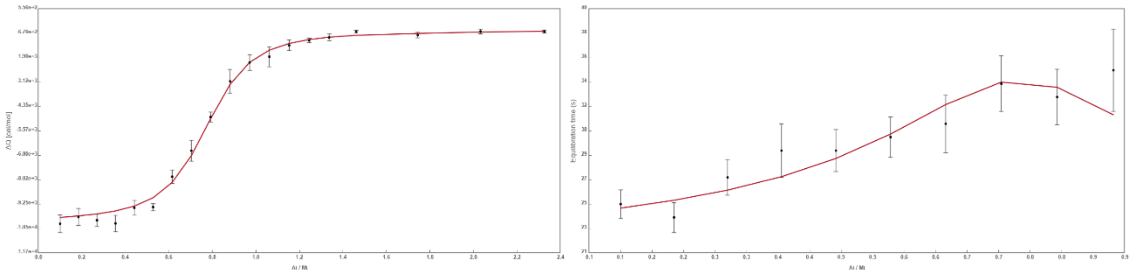

Experiment 3

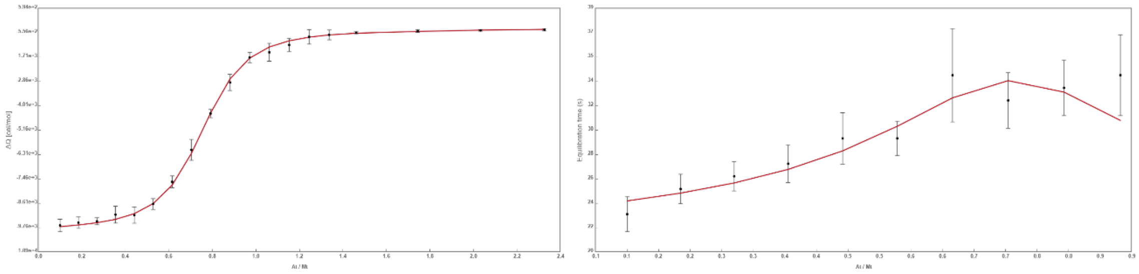

Compound 6

Before global fitting

Experiment 1

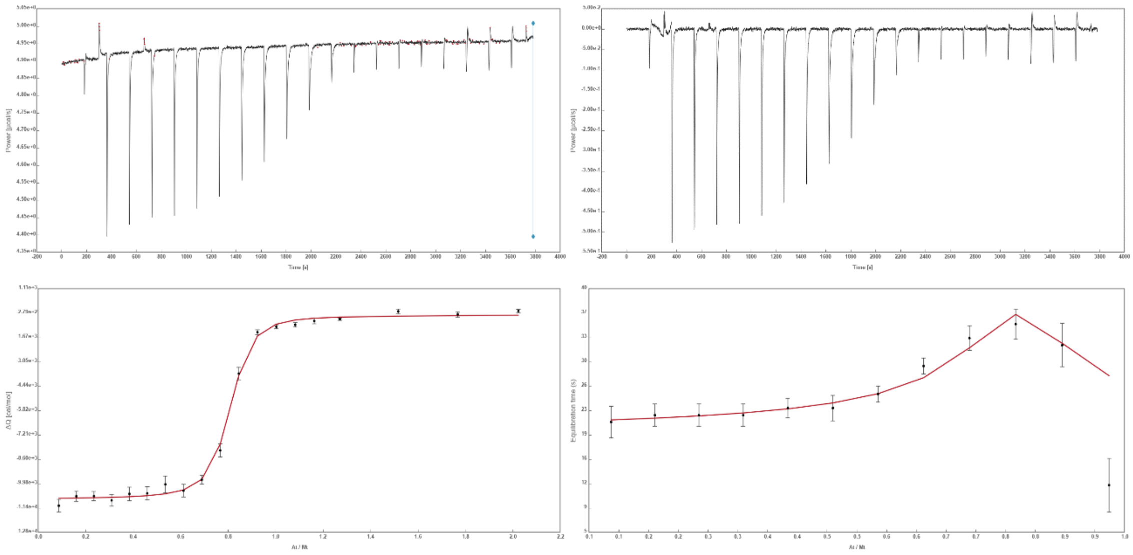

Experiment 2

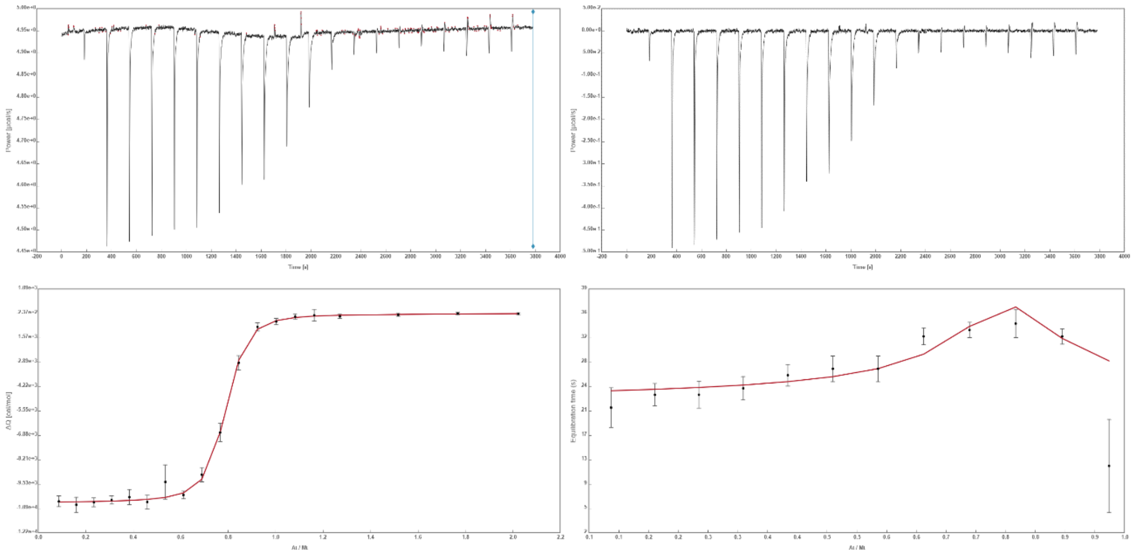

Experiment 3

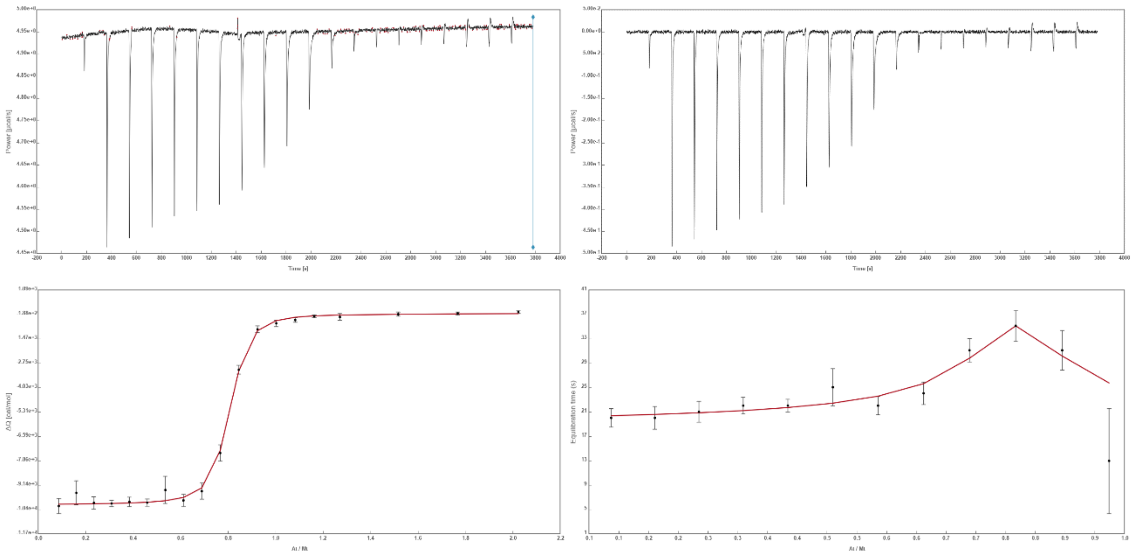

<u>Global fitting</u>

Experiment 1

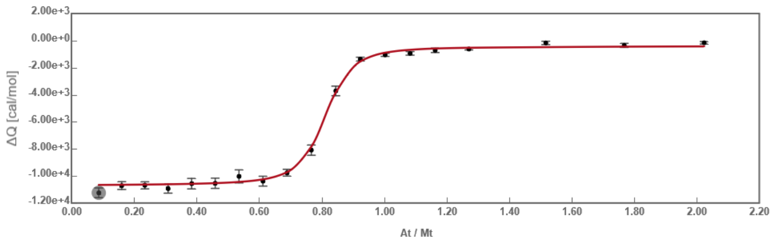

Experiment 2

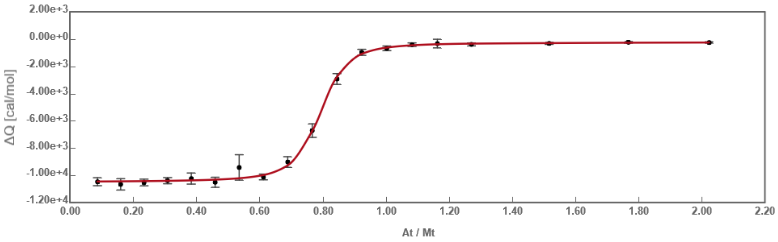

Experiment 3

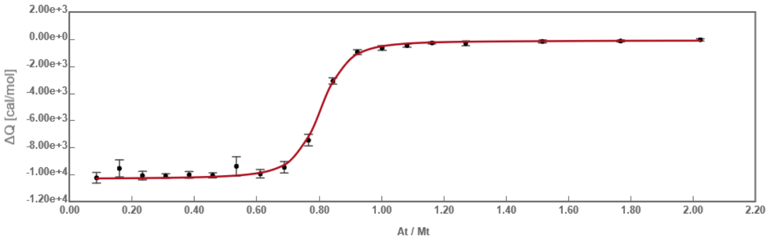

<u>After global fitting</u>

Experiment 1

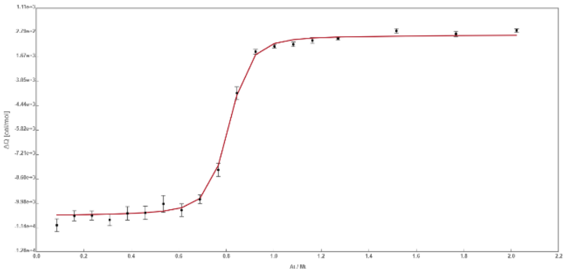

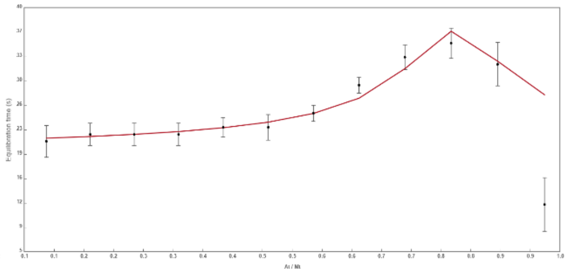

Experiment 2

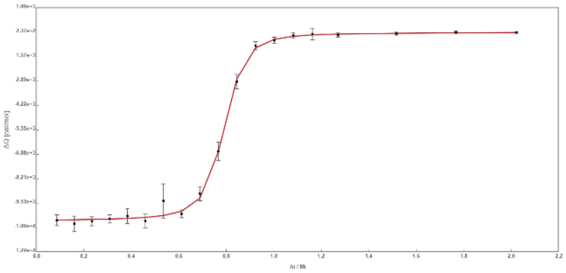

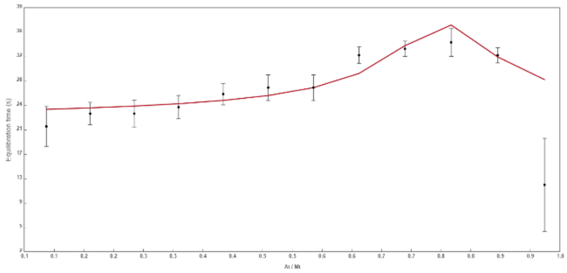

Experiment 3

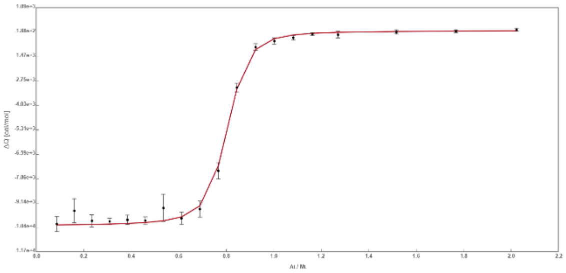

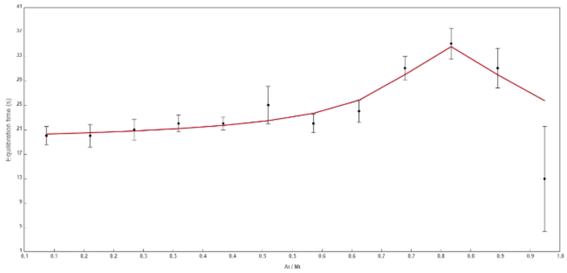

Compound 7

<u>Before global fitting</u>

Experiment 1

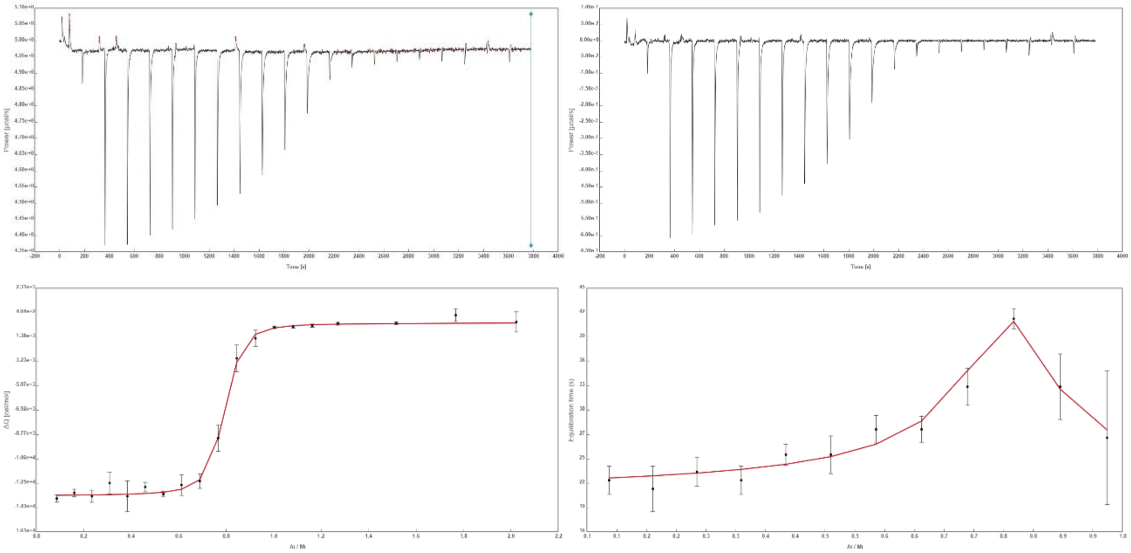

Experiment 2

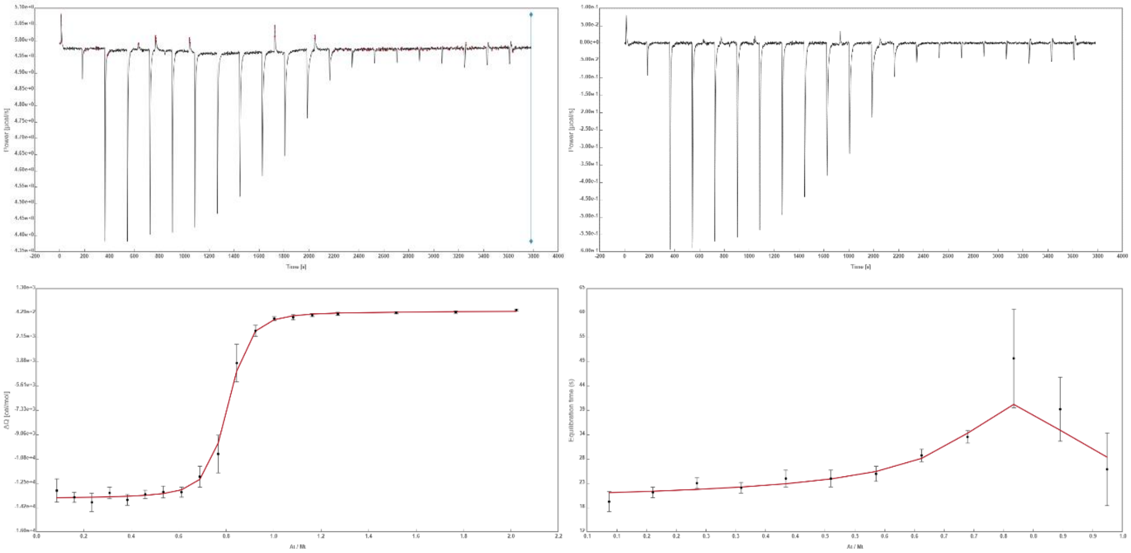

Experiment 3

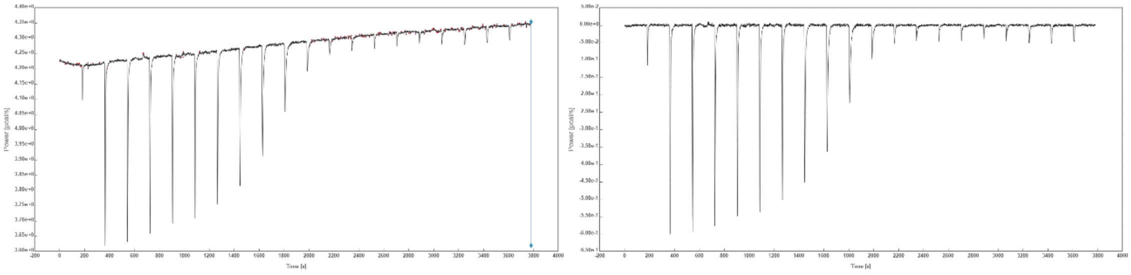

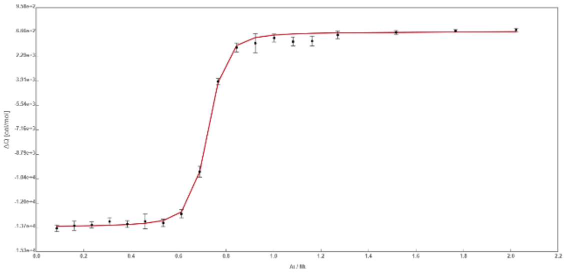 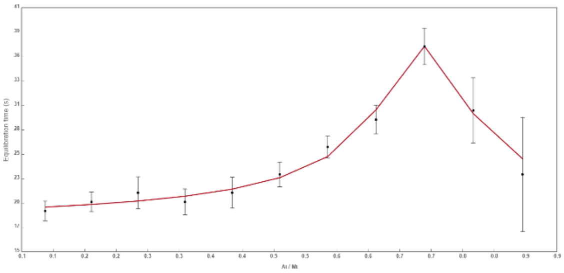

Global fitting

Experiment 1

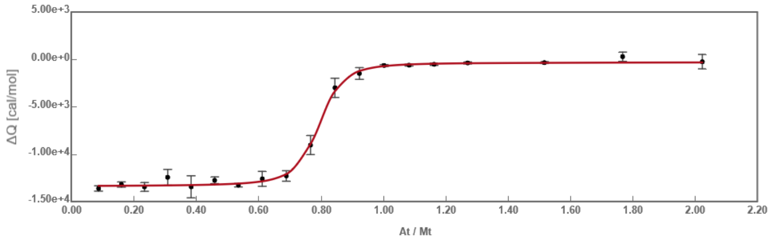

Experiment 2

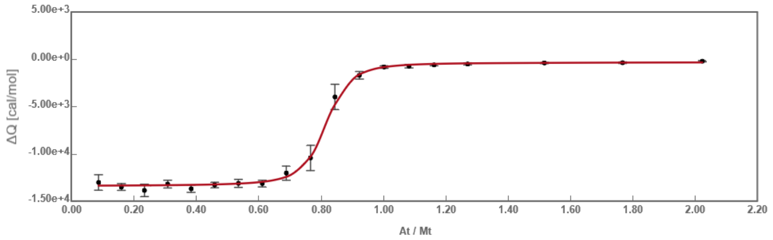

Experiment 3

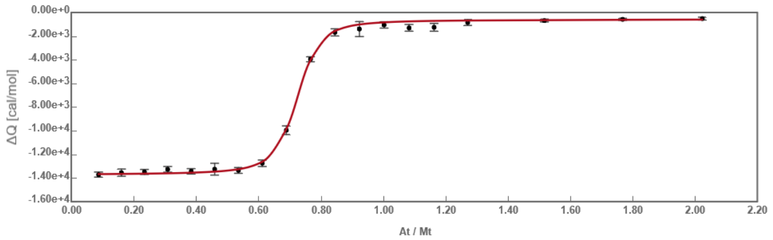

After global fitting

Experiment 1

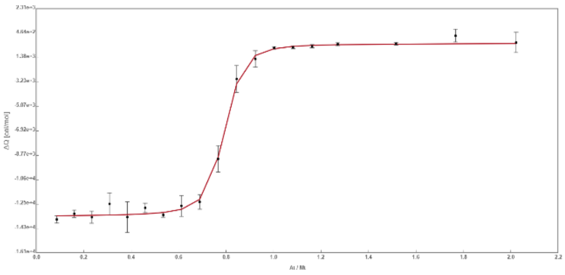 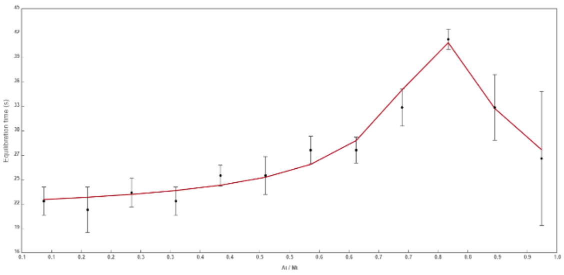

Experiment 2

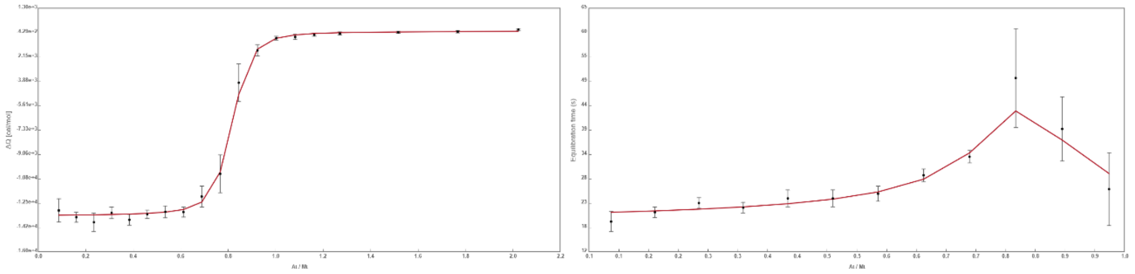

Experiment 3

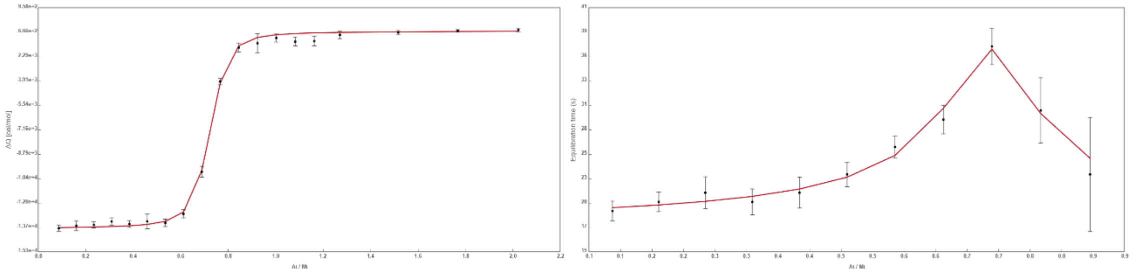

Compound 8

<u>Before global fitting</u>

Experiment 1

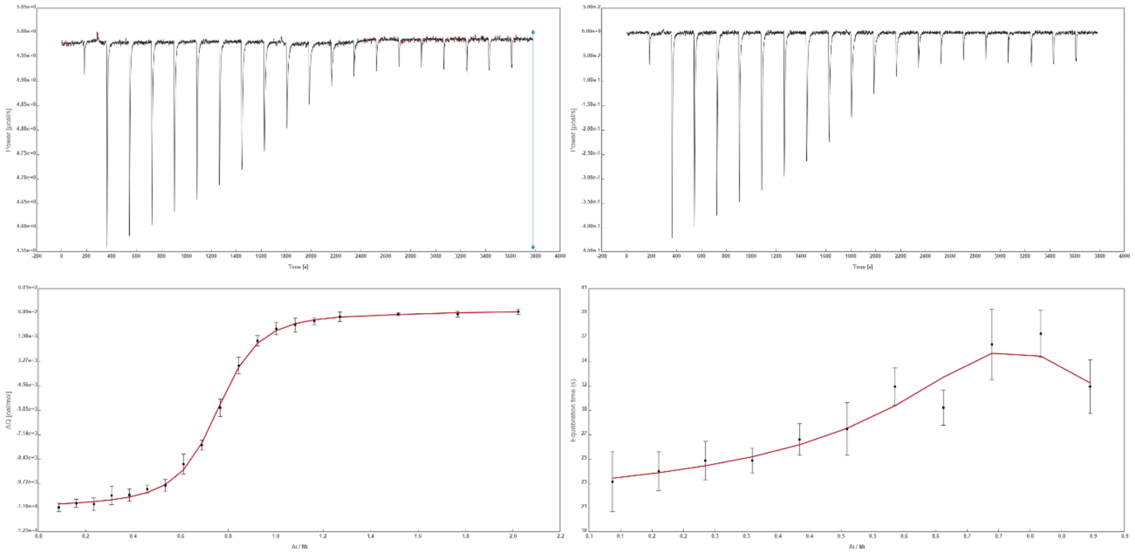

Experiment 2

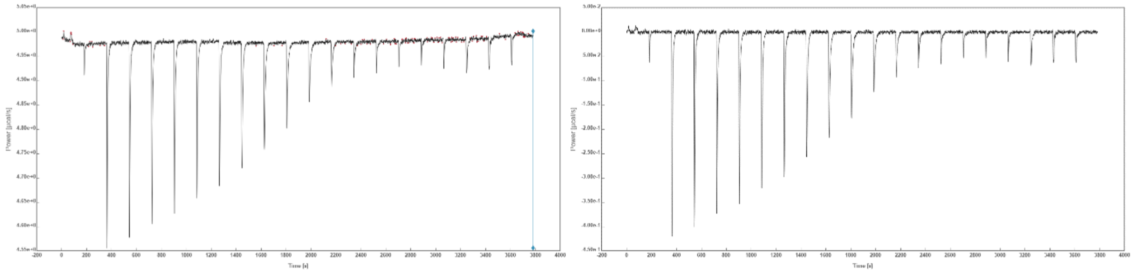

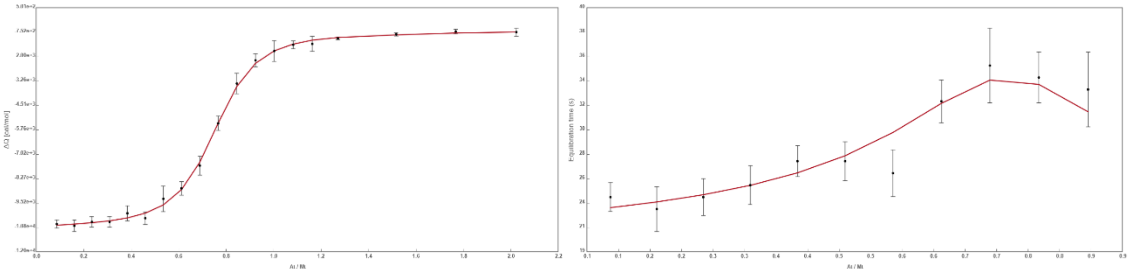

Experiment 3

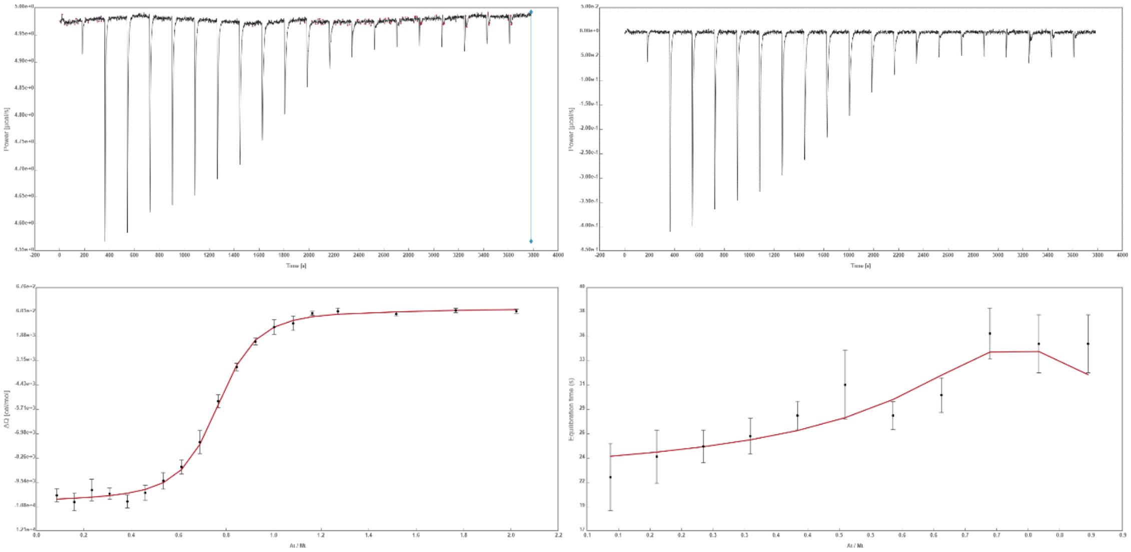

<u>Global fitting</u>

Experiment 1

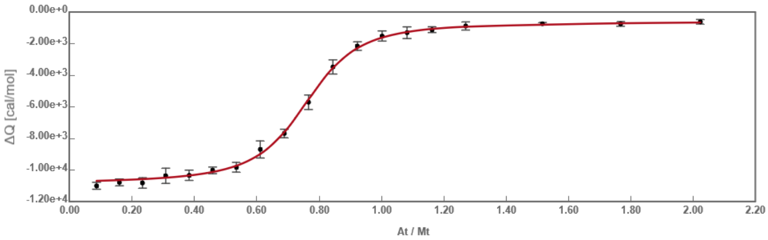

Experiment 2

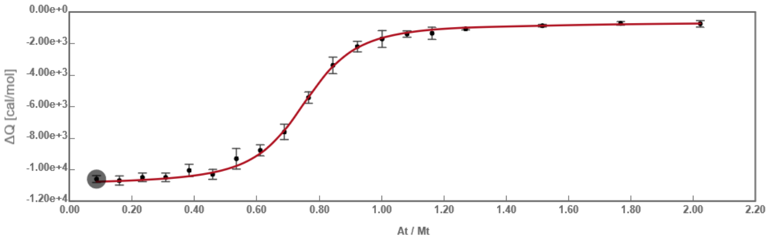

Experiment 3

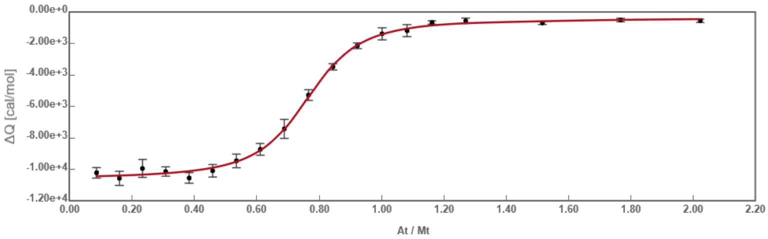

<u>After global fitting</u>

Experiment 1

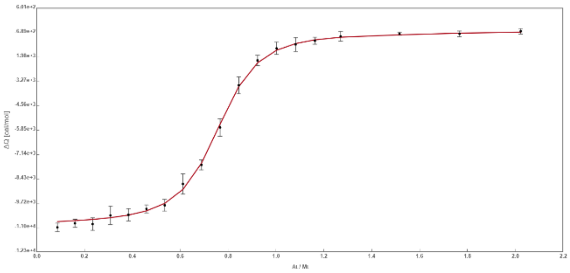

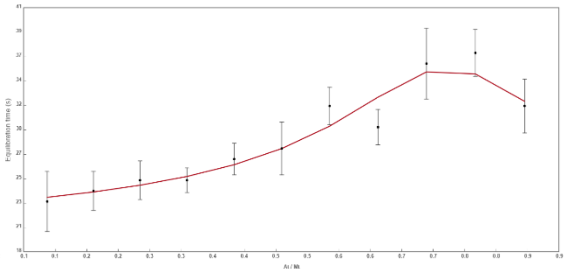

Experiment 2

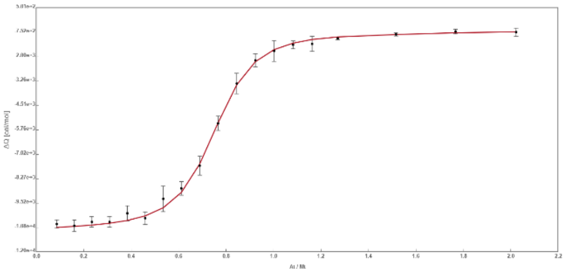

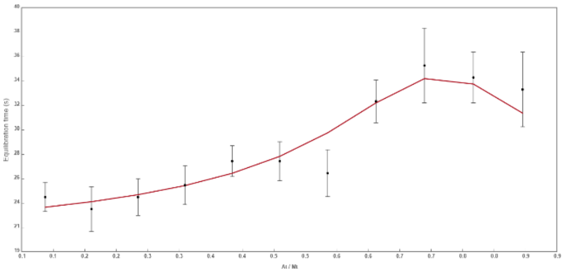

Experiment 3

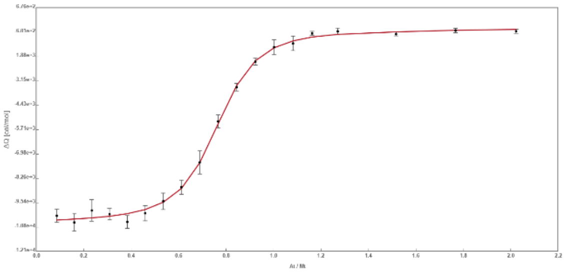

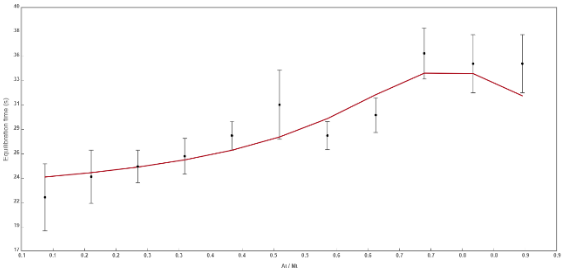

Compound 9

<u>Before global fitting</u>

Experiment 1

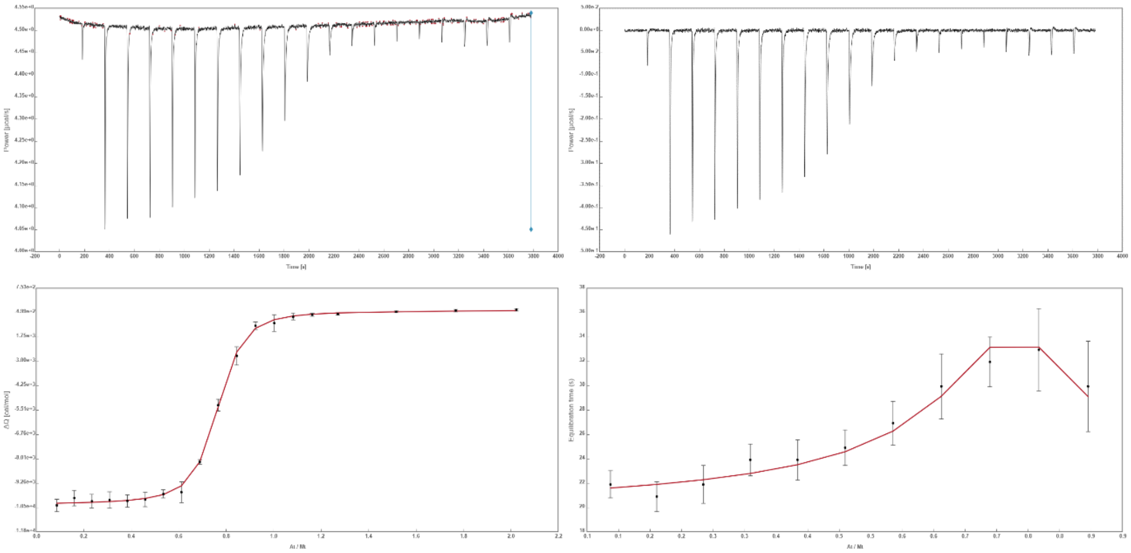

Experiment 2

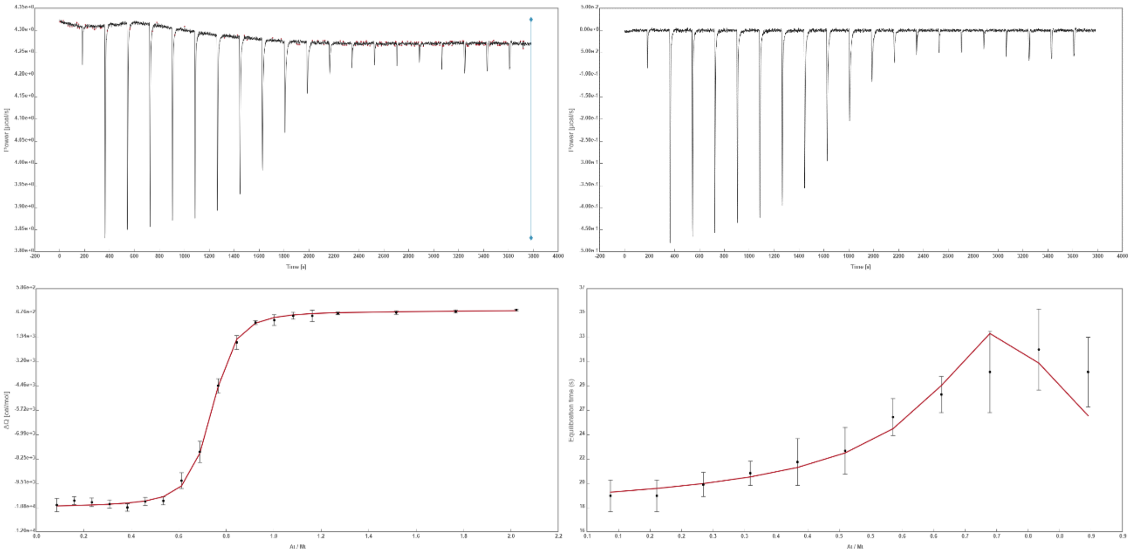

Experiment 3

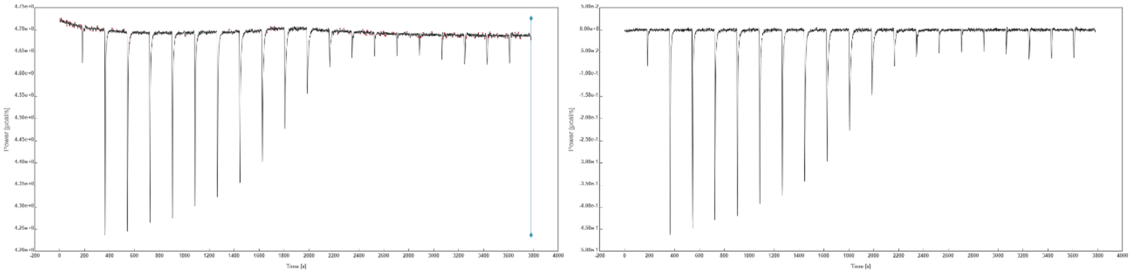

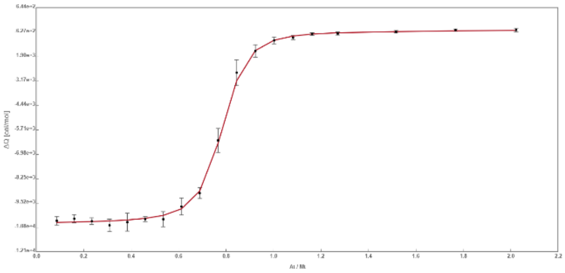

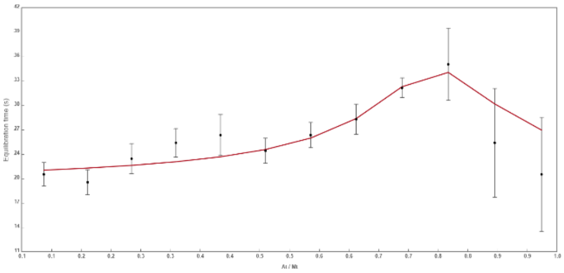

Global fitting

Experiment 1

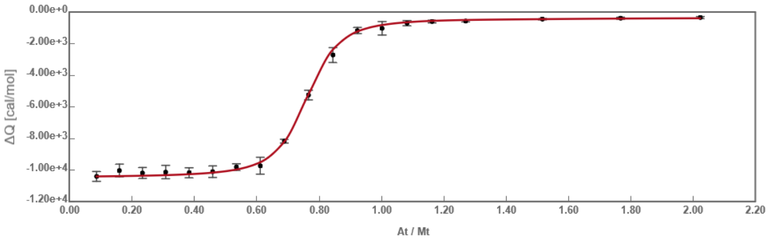

Experiment 2

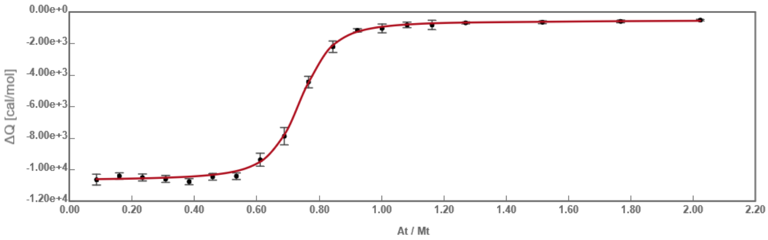

Experiment 3

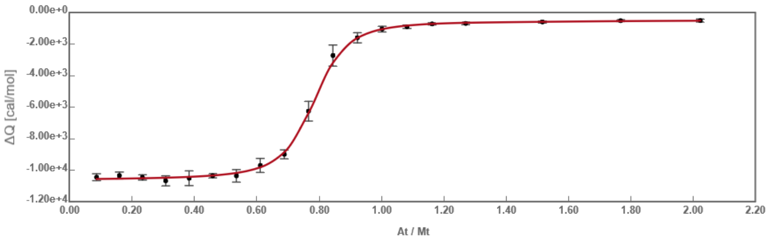

After global fitting

Experiment 1

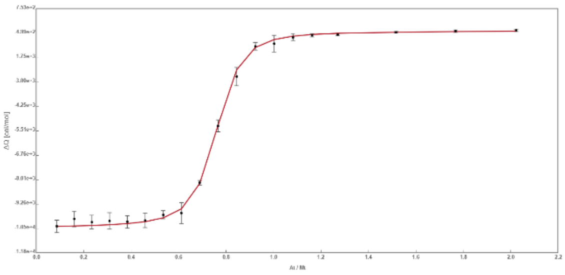

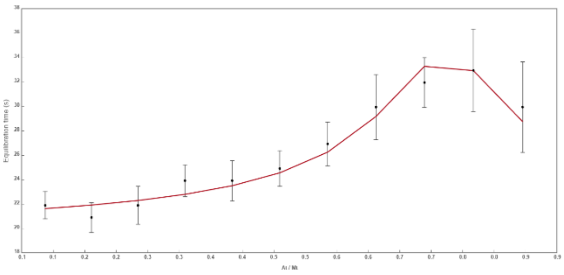

Experiment 2

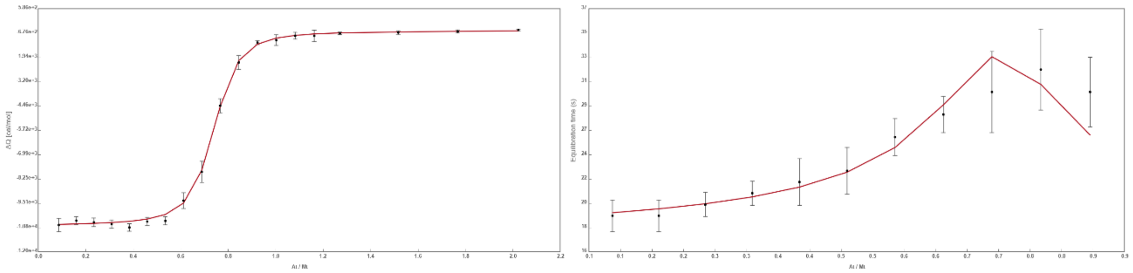

Experiment 3

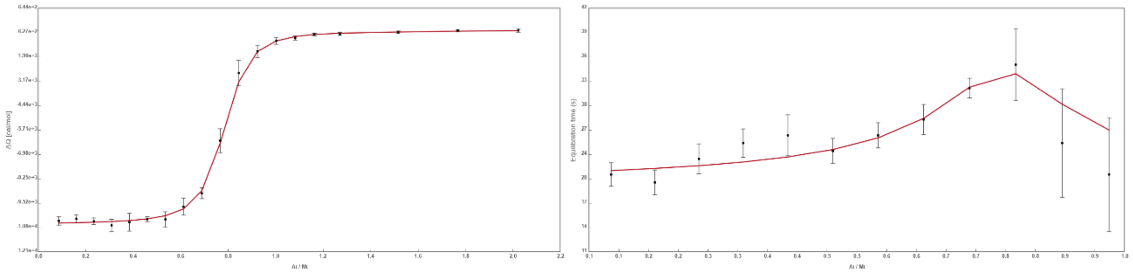

Compound 10

<u>Before global fitting</u>

Experiment 1

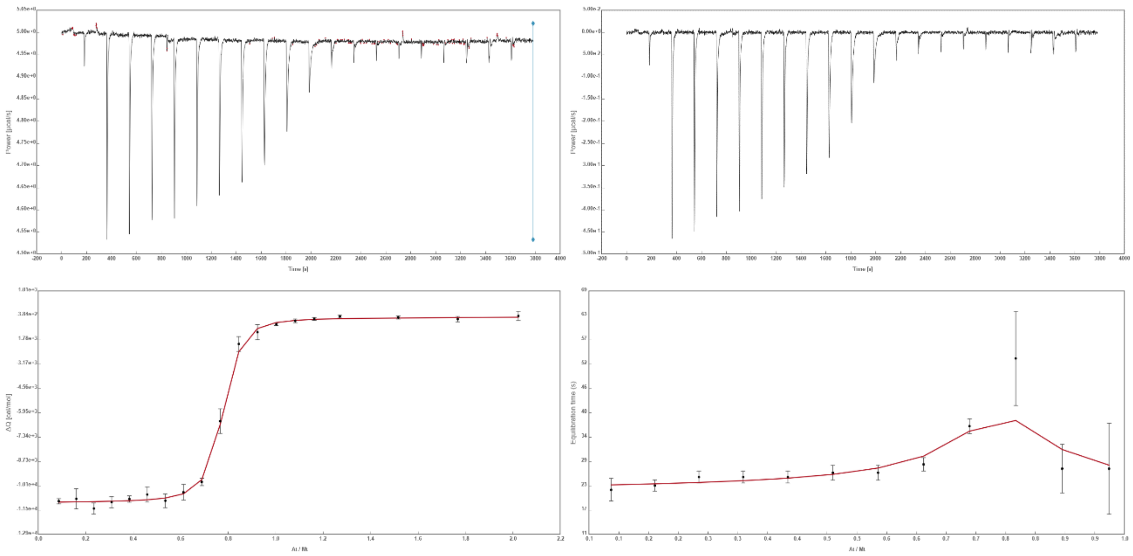

Experiment 2

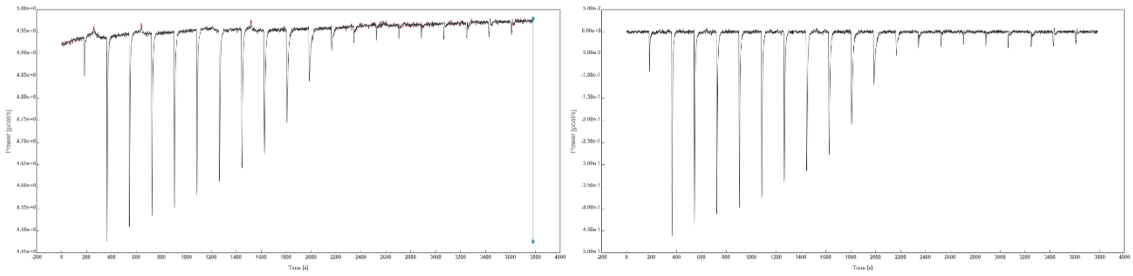

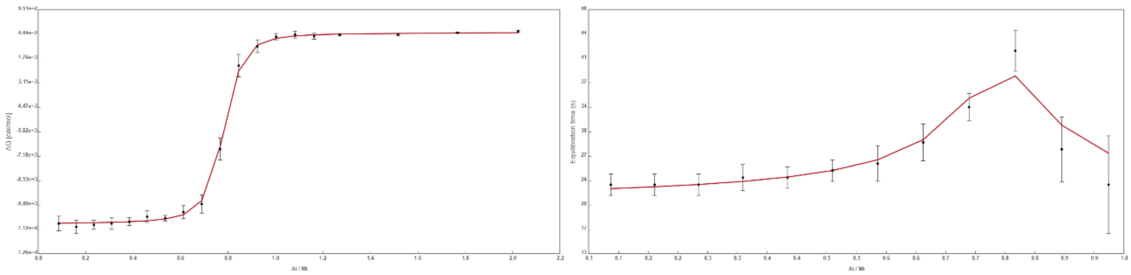

Experiment 3

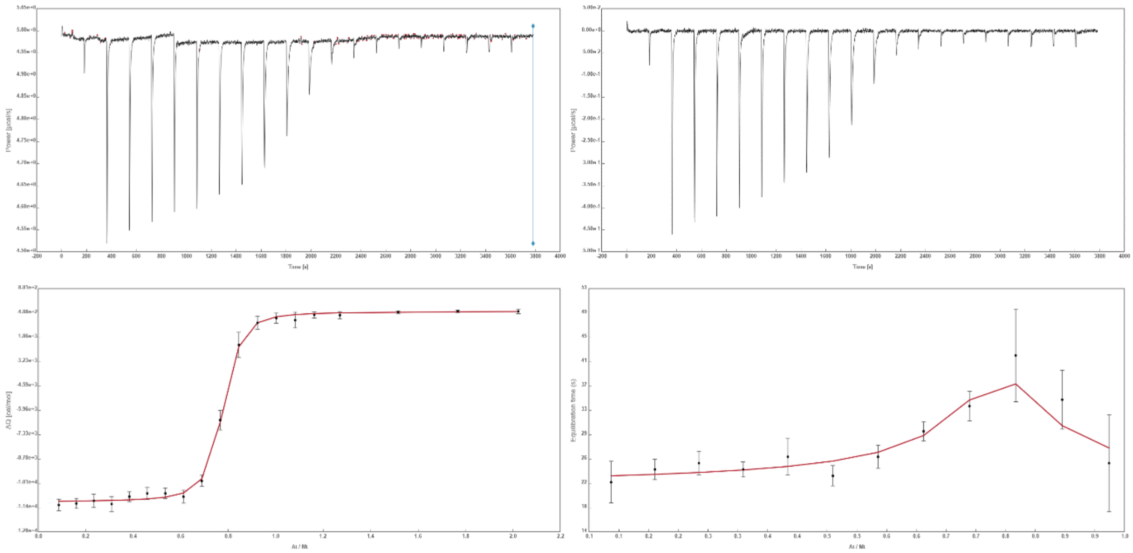

Global fitting

Experiment 1

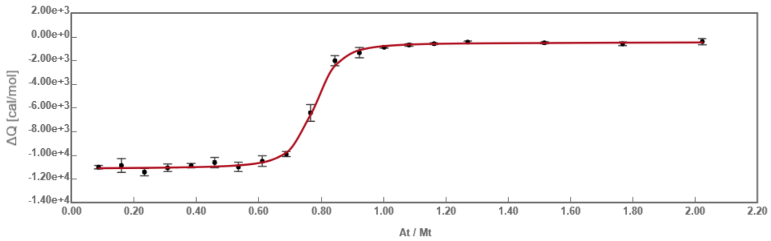

Experiment 2

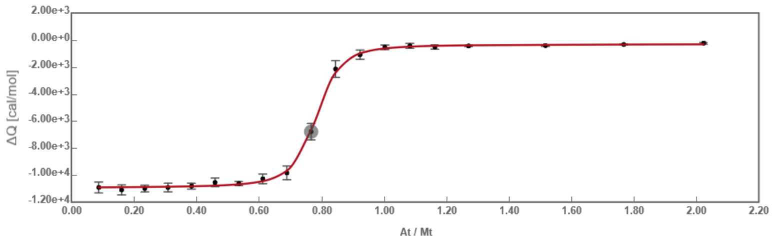

Experiment 3

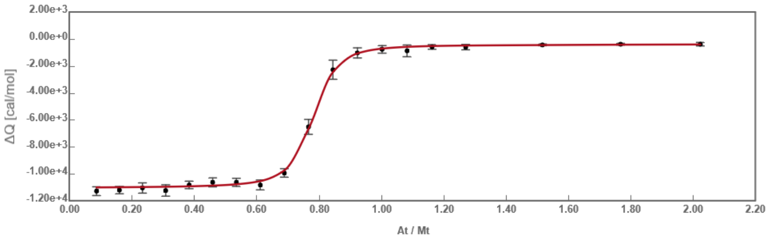

<u>After global fitting</u>

Experiment 1

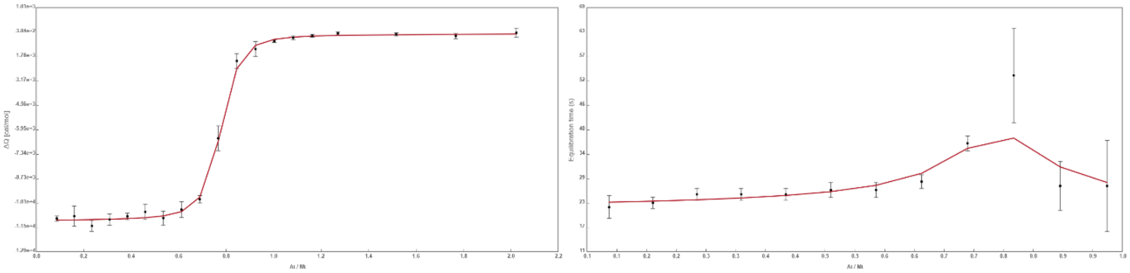

Experiment 2

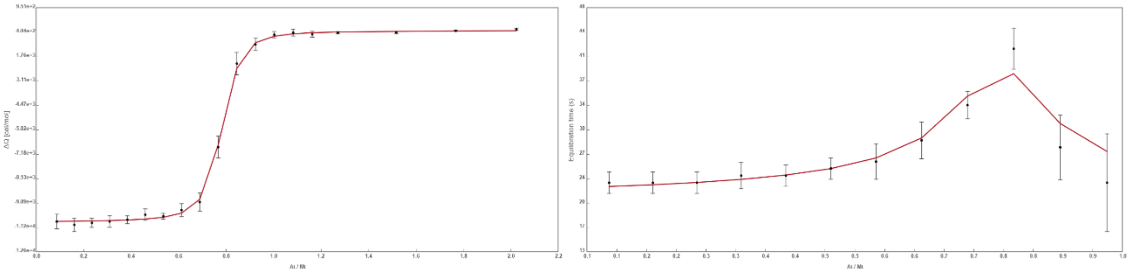

Experiment 3

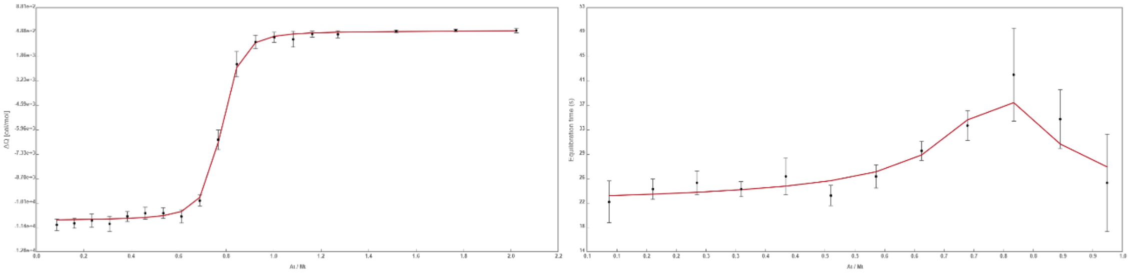

Reference compound 4CBS

Experiment 1

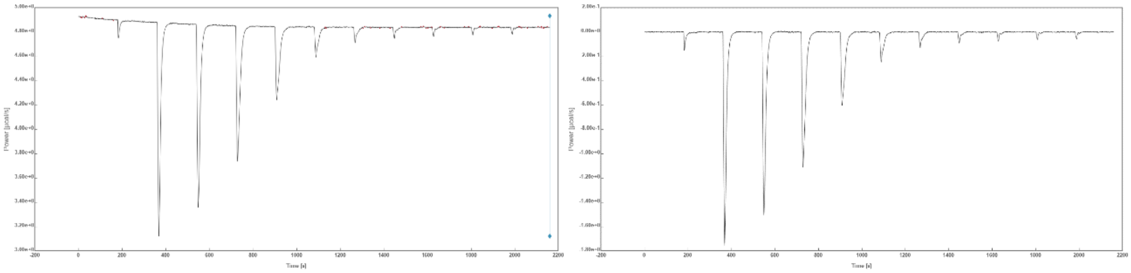

Experiment 2

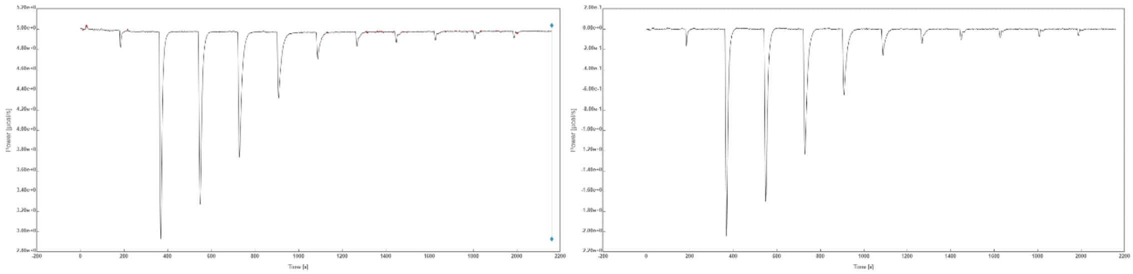

Experiment 3

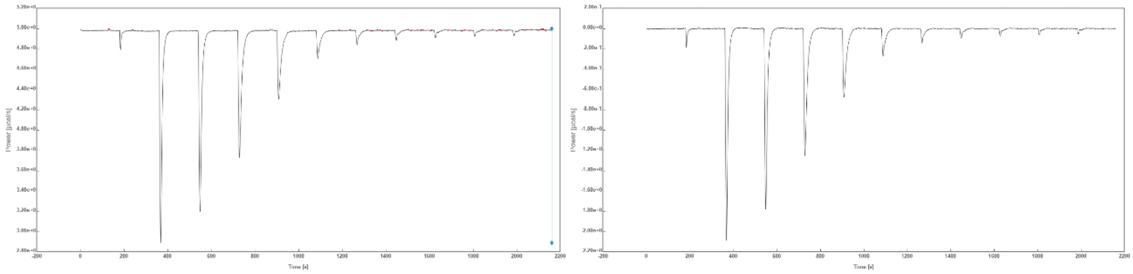

Compound 11

<u>Direct titrations</u>

Experiment 1

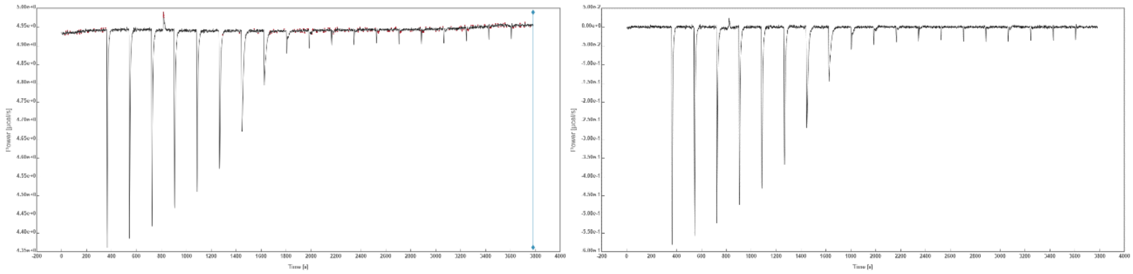

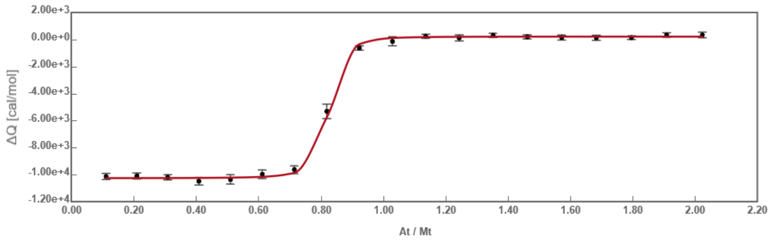

Experiment 2

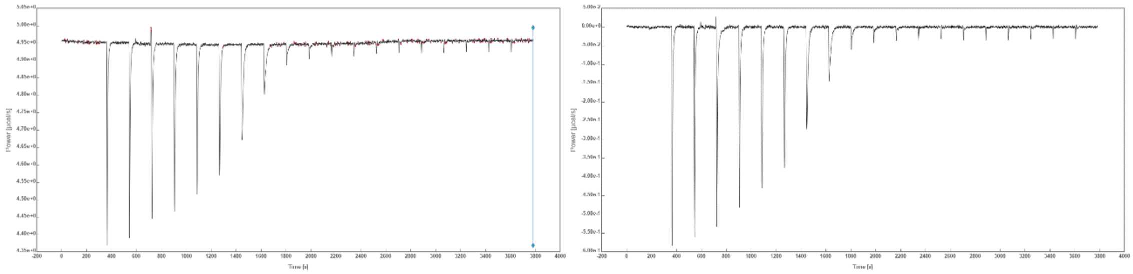

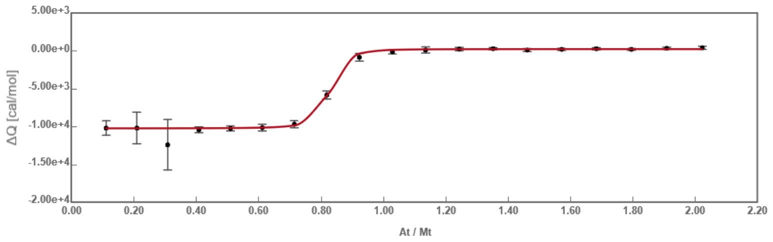

Experiment 3

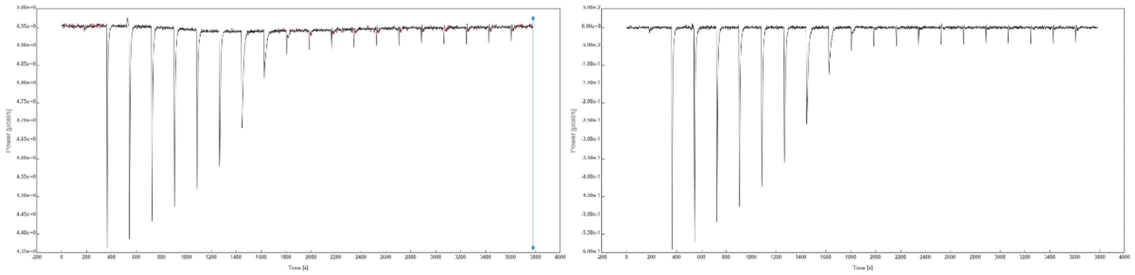

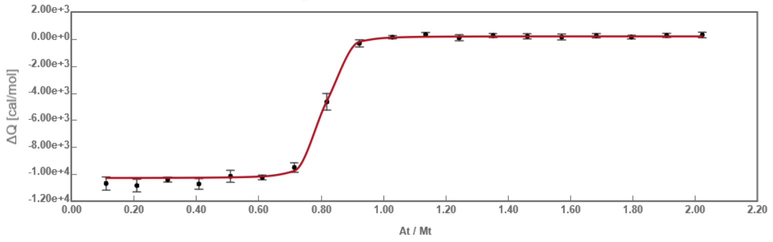

<u>Reference 4CBS</u>

Experiment 1

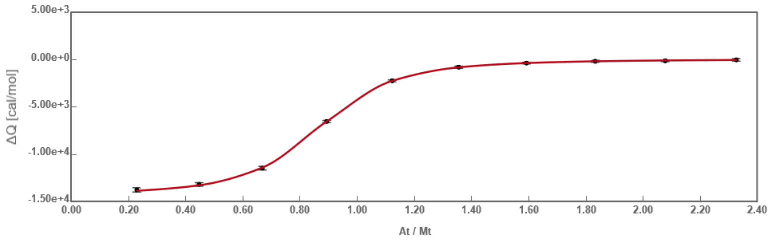

Experiment 2

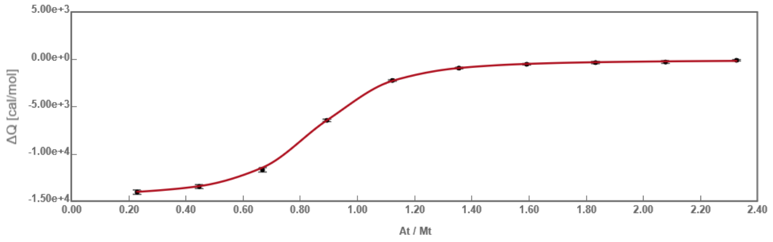

Experiment 3

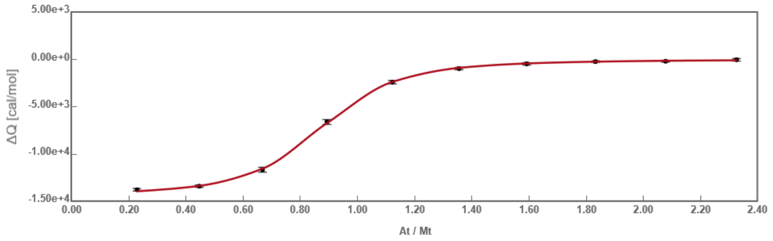

Displacement of 4CBS

Experiment 1

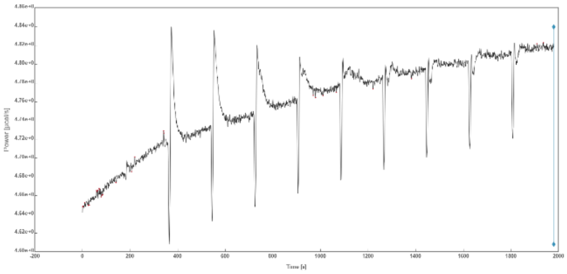
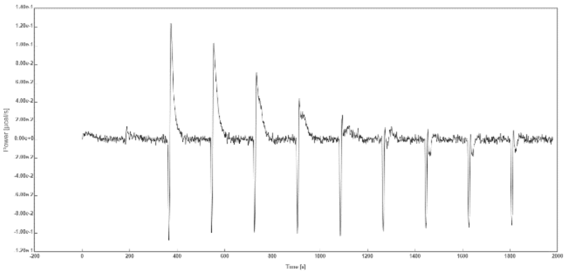

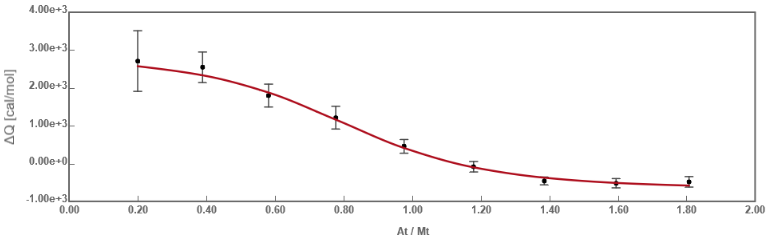

Experiment 2

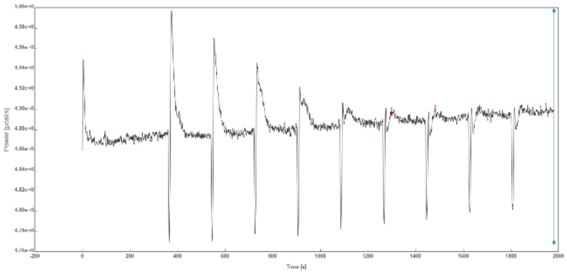
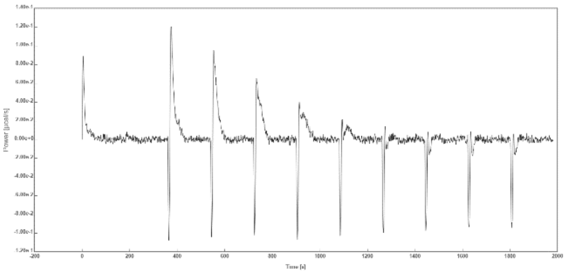

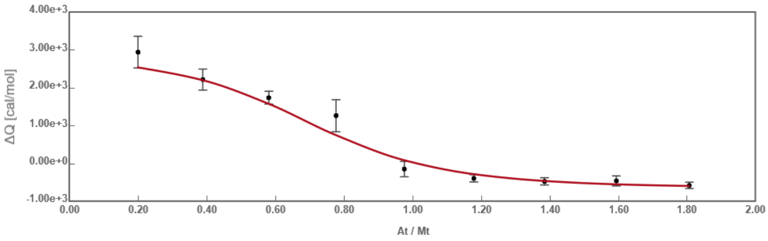

Compound 12

Direct titrations

Experiment 1

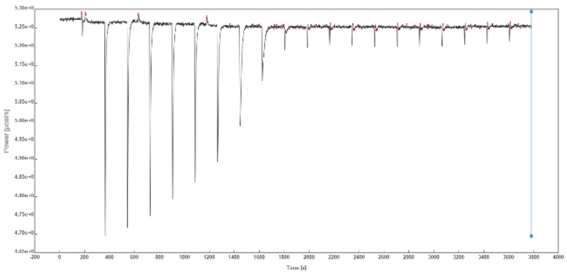
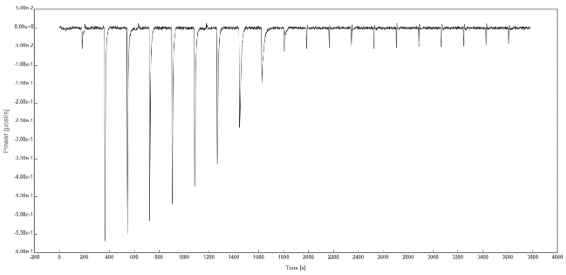

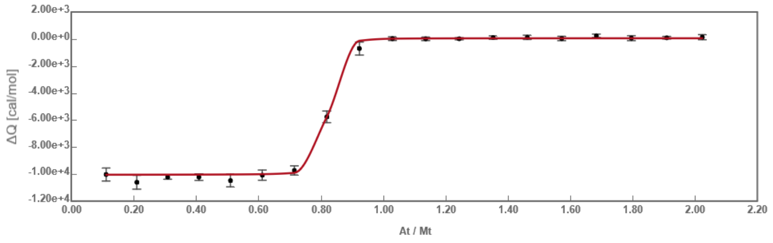

Experiment 2

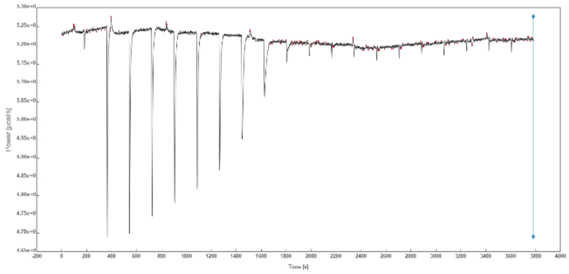

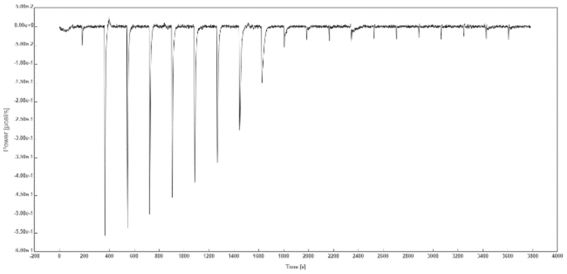

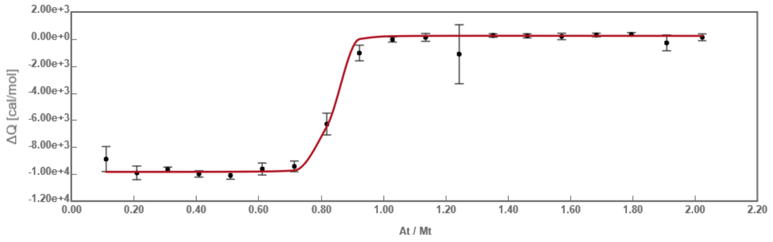

Experiment 3

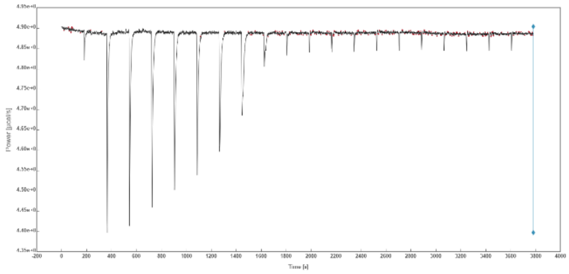

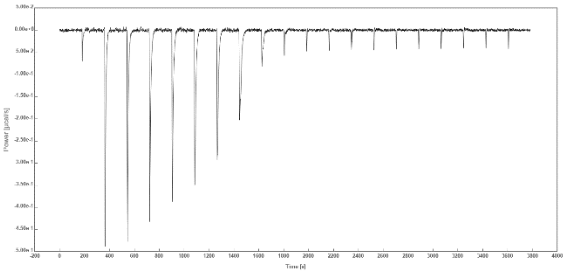

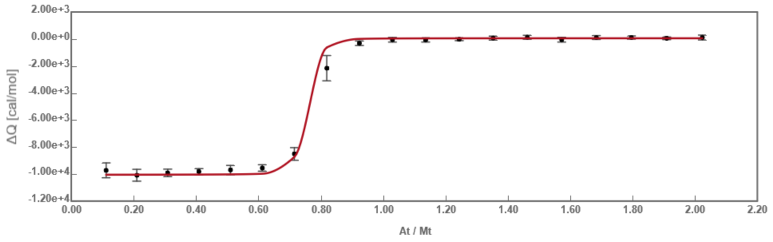

<u>Reference 4CBS</u>

Experiment 1

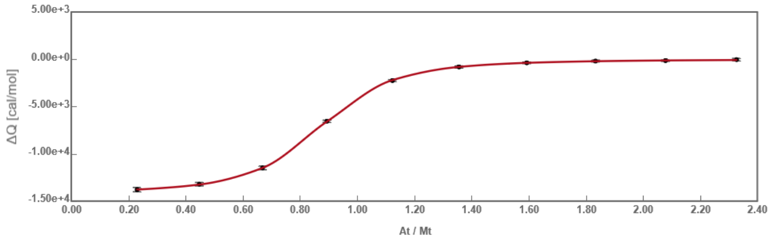

Experiment 2

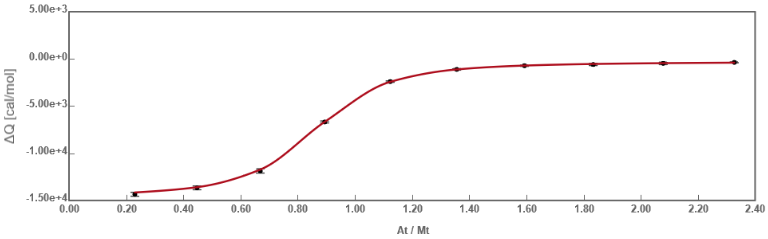

Experiment 3

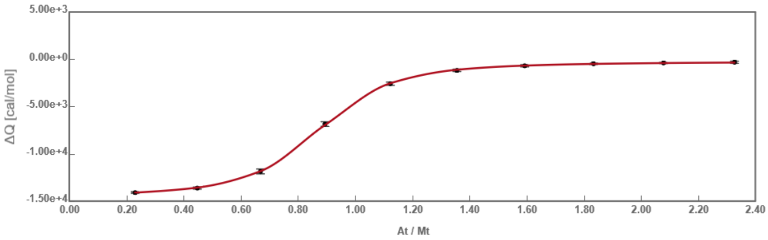

<u>Displacement of 4CBS</u>

Experiment 1

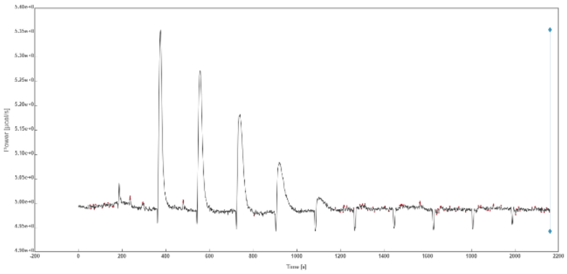

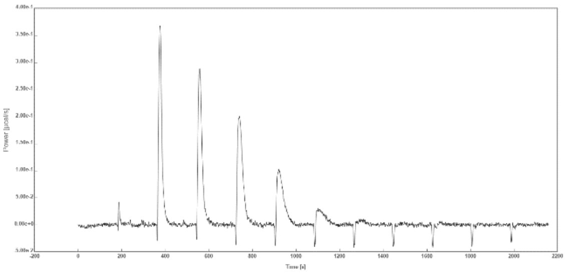

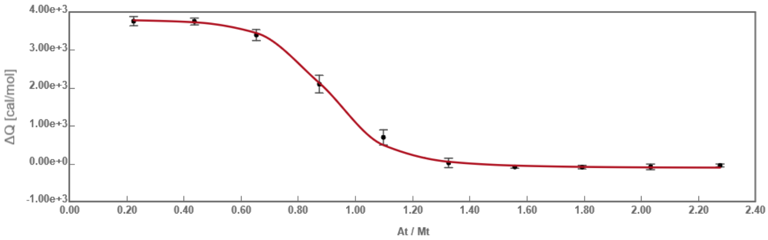

Experiment 2

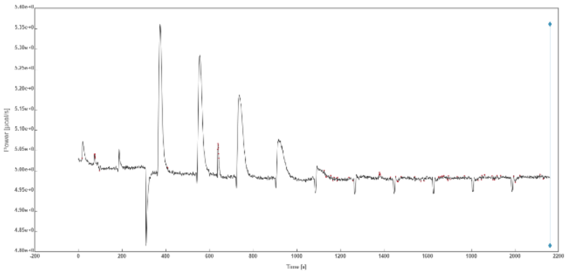

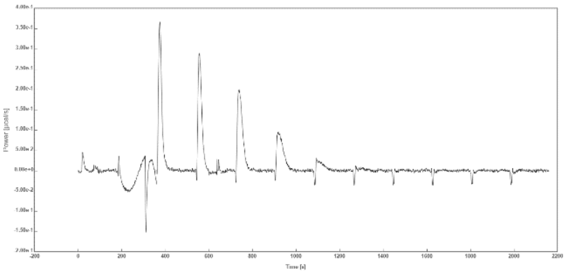

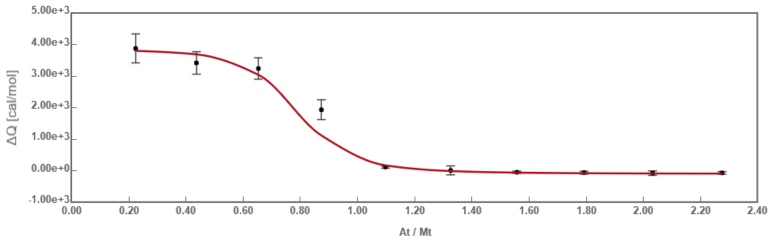

Experiment 3

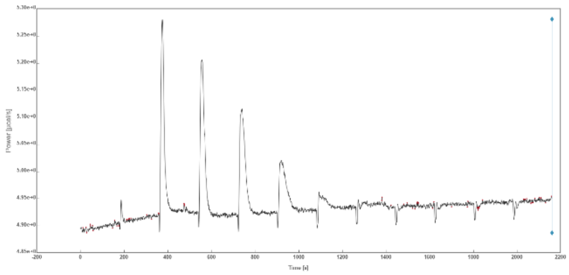

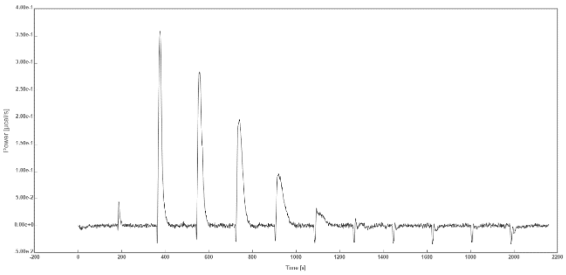

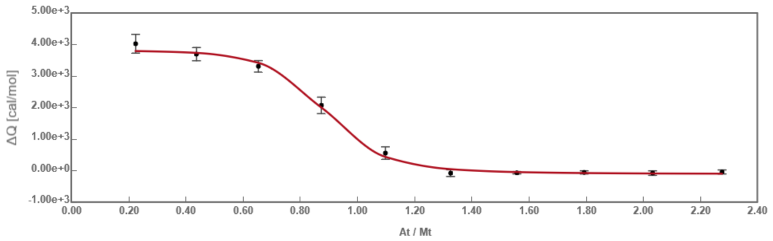

Experiment 4

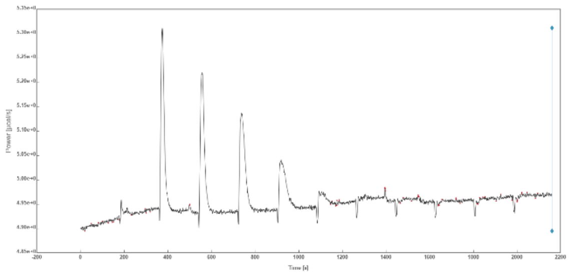

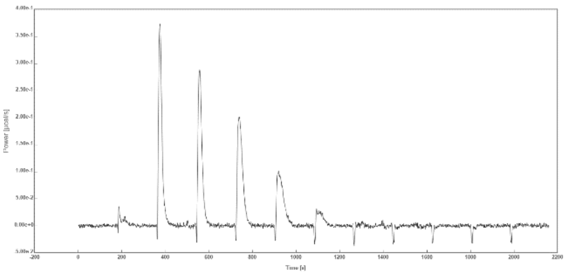

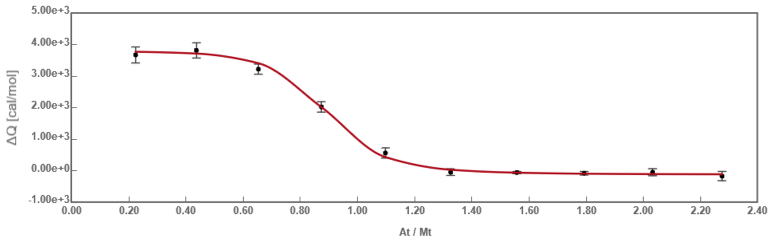

Compound 13

<u>Direct titrations</u>

Experiment 1

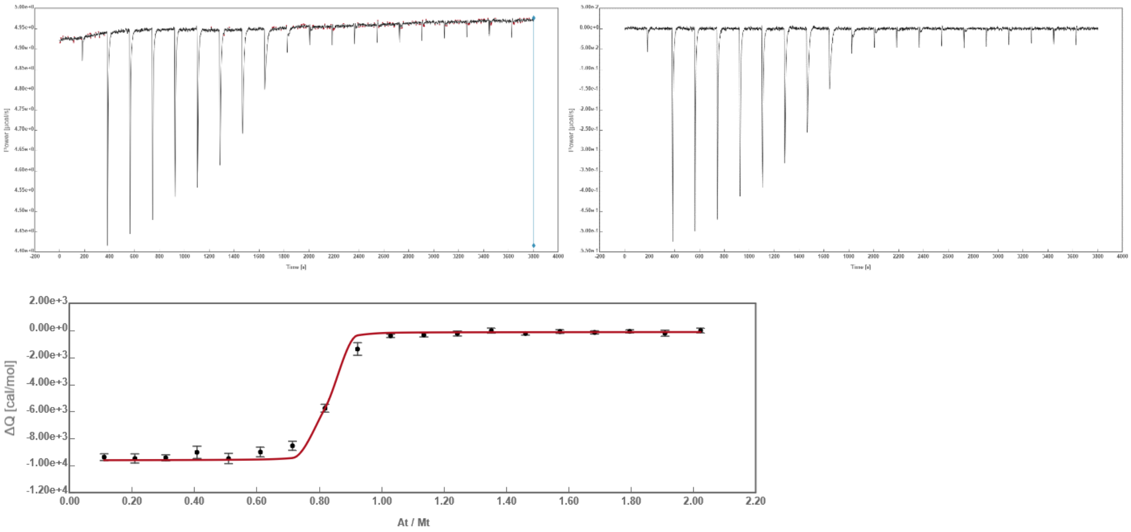

Experiment 2

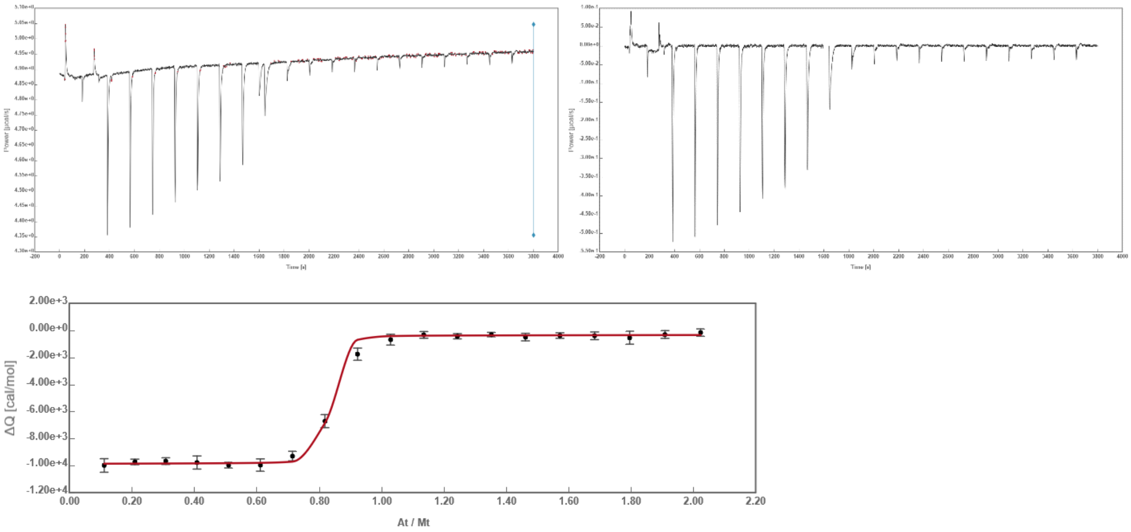

Experiment 3

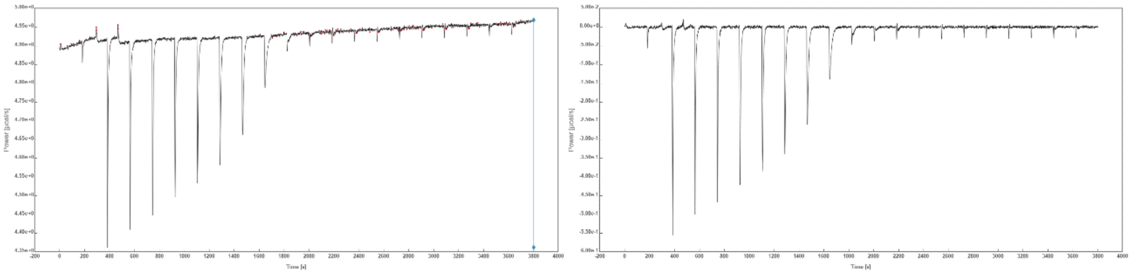

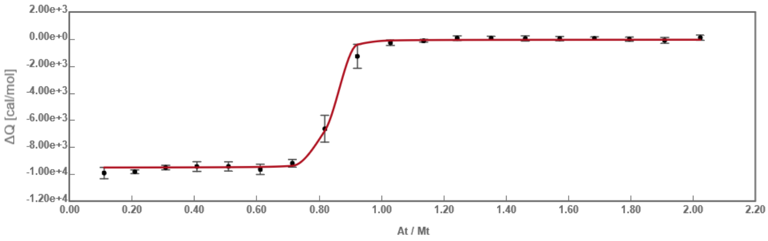

<u>Reference 4CBS</u>

Experiment 1

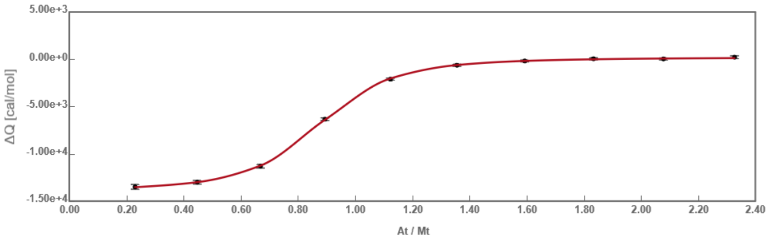

Experiment 2

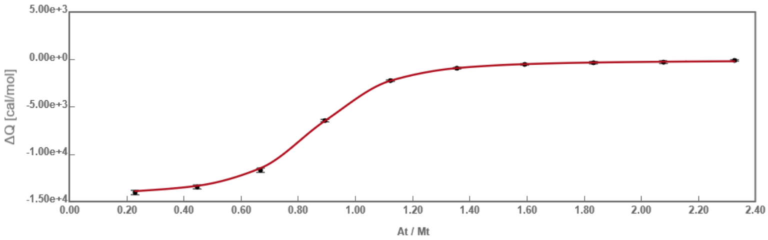

Experiment 3

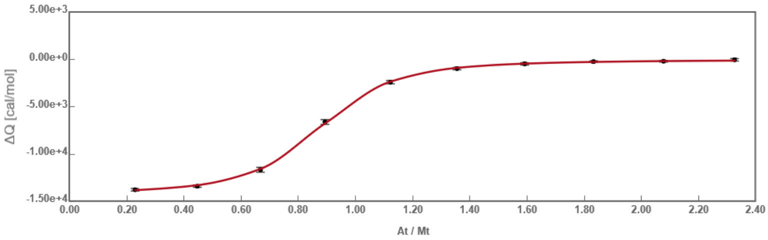

<u>Displacement of 4CBS</u>

Experiment 1

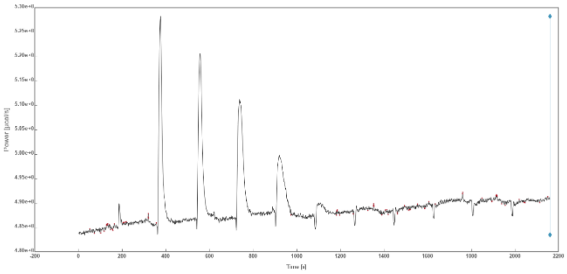

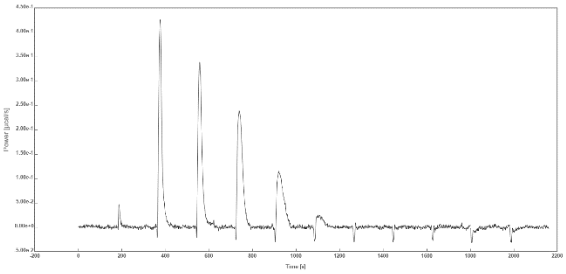

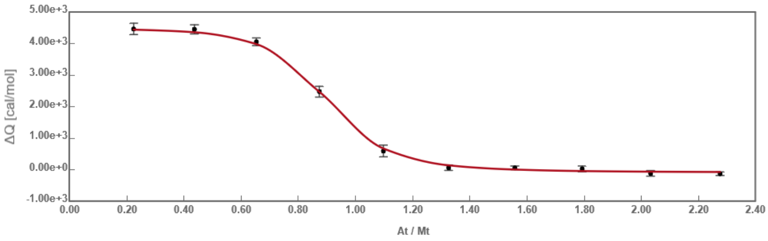

Experiment 2

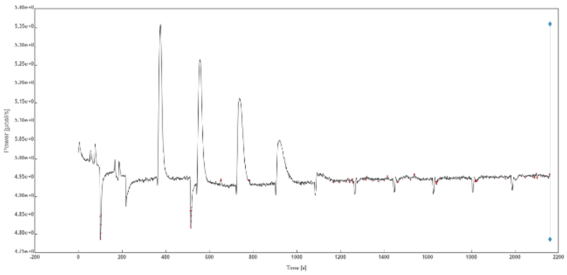

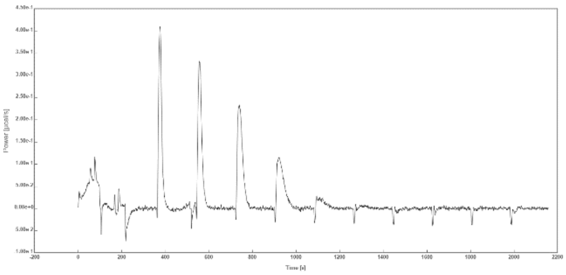

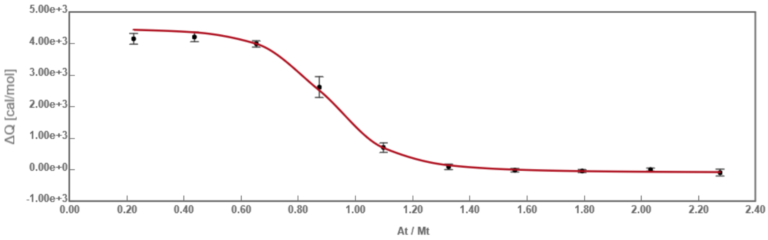

Experiment 3

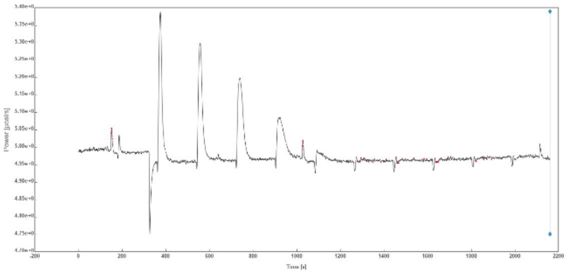

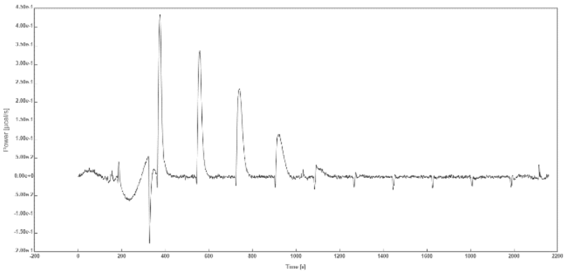

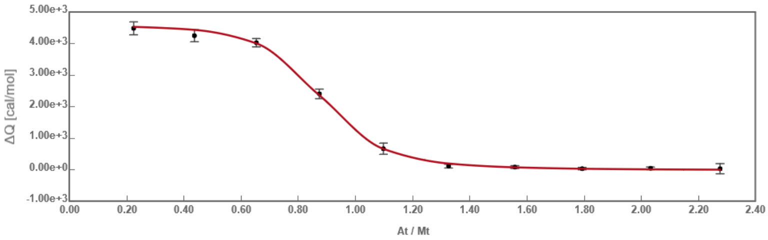

Compound 15

Before global fitting

Experiment 1

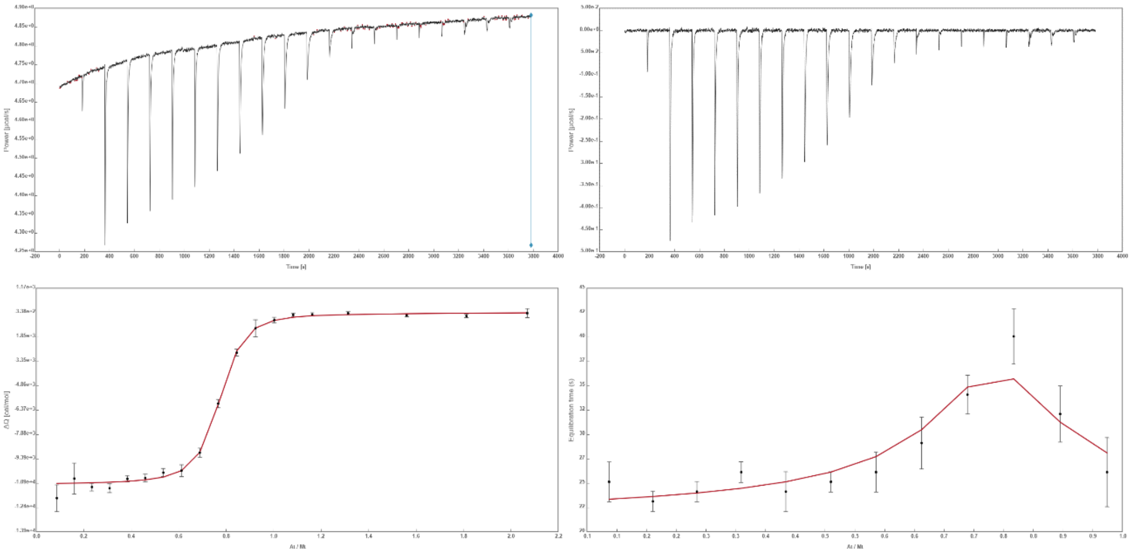

Experiment 2

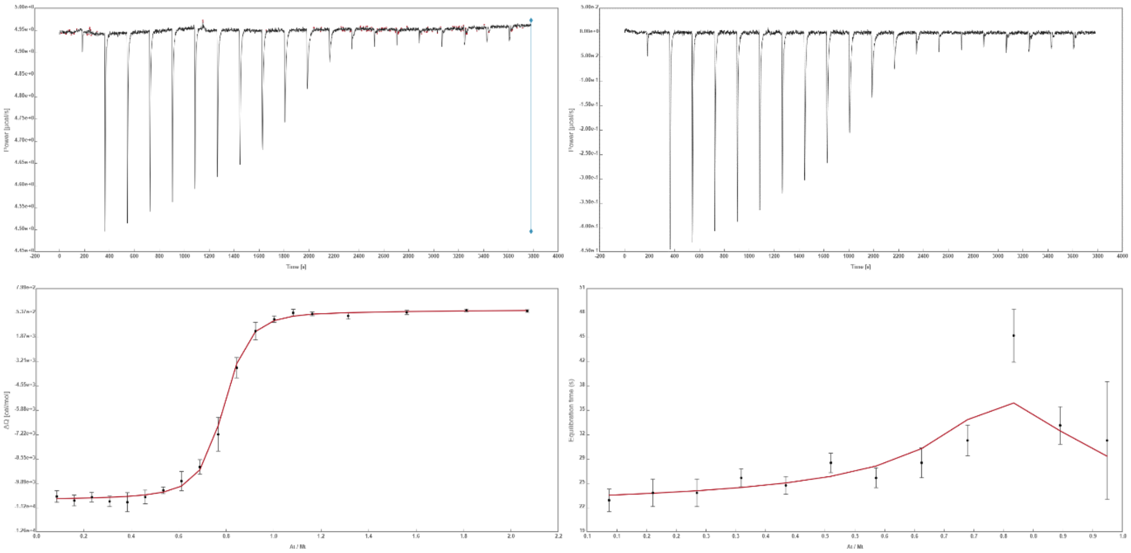

Experiment 3

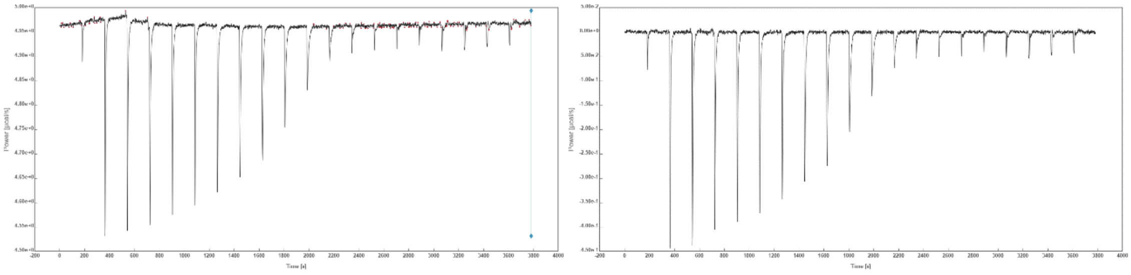

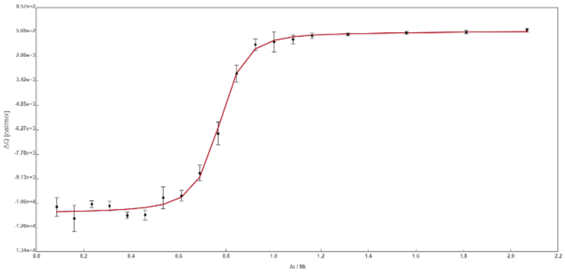

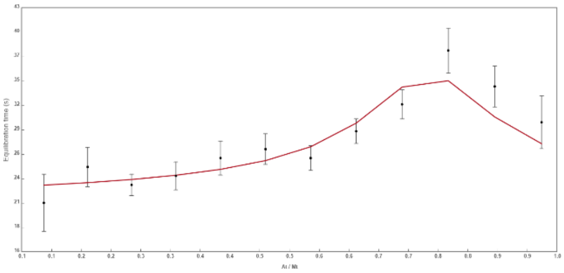

Global fitting

Experiment 1

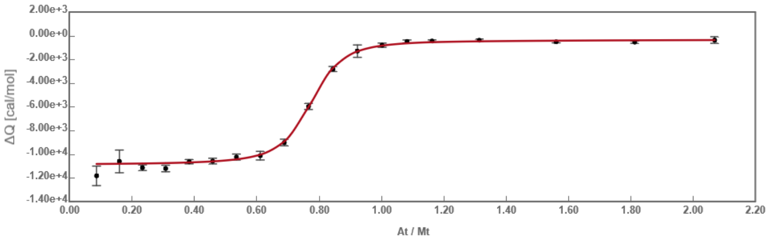

Experiment 2

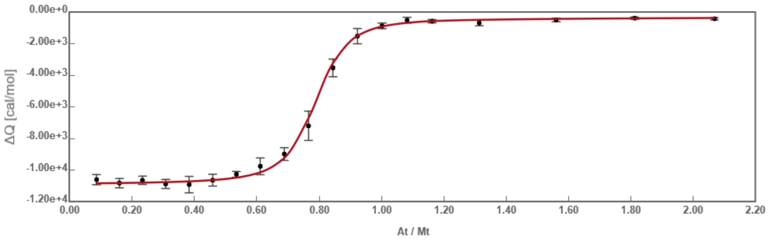

Experiment 3

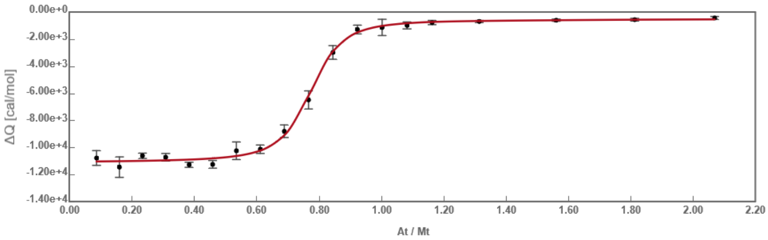

After global fitting

Experiment 1

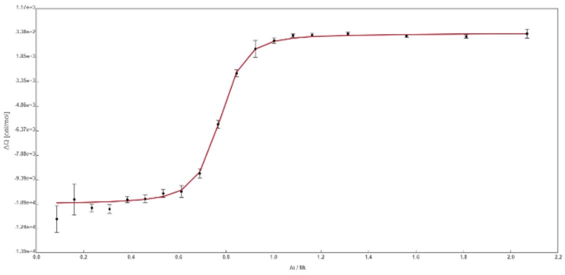

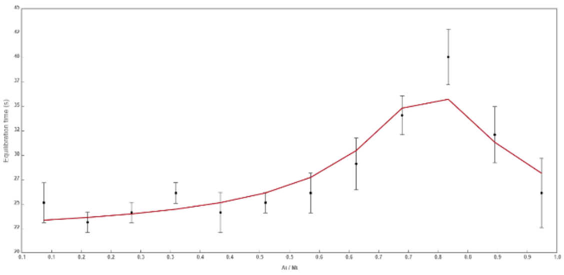

Experiment 2

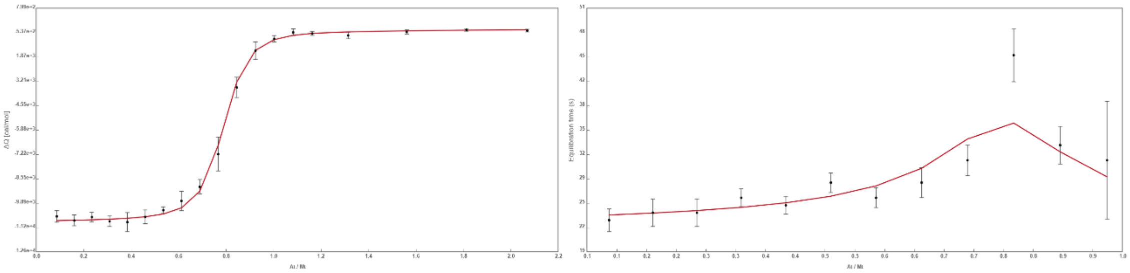

Experiment 3

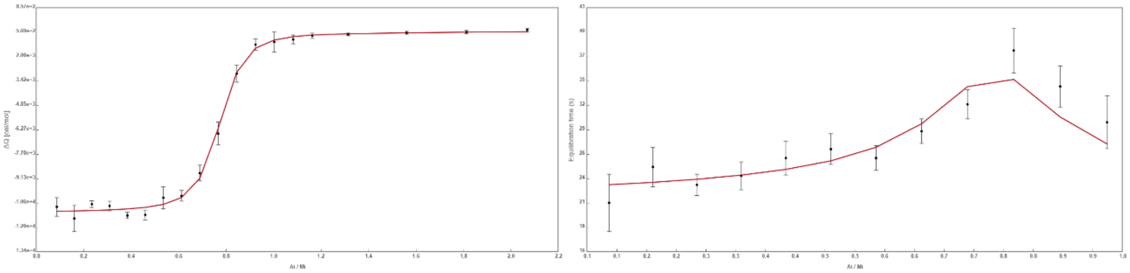

Compound 16

<u>Before global fitting</u>

Experiment 1

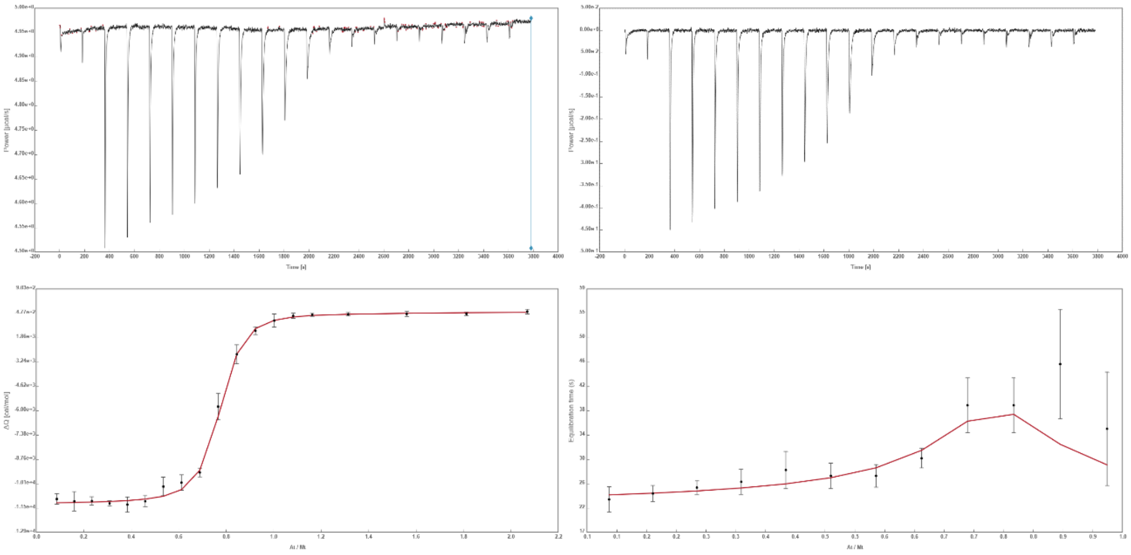

Experiment 2

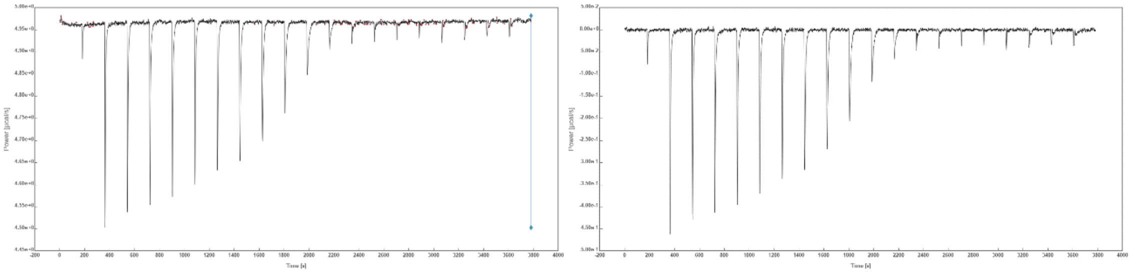

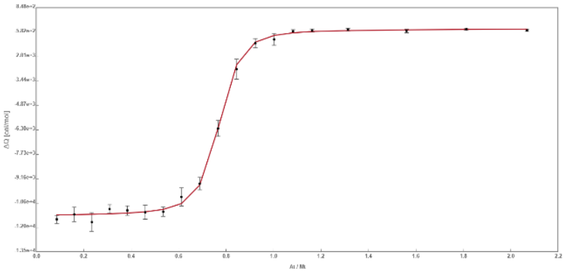

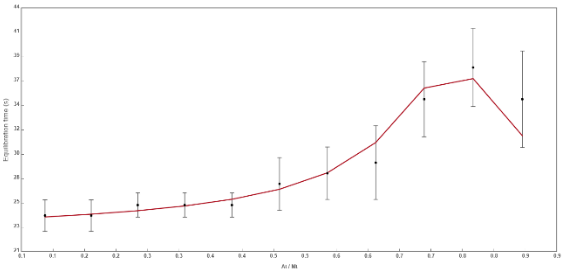

Experiment 3

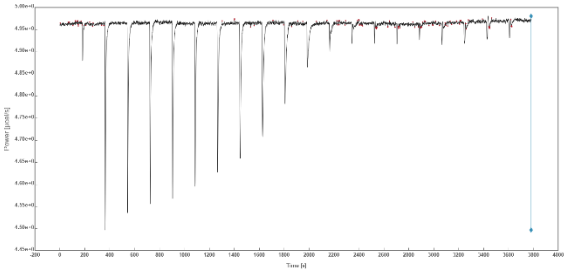

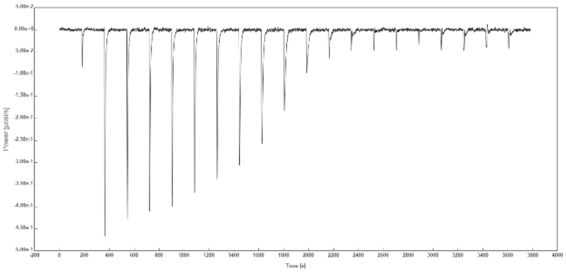

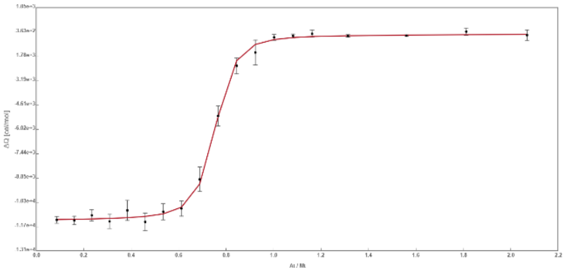

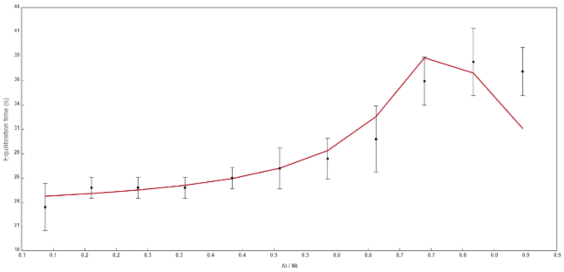

<u>Global fitting</u>

Experiment 1

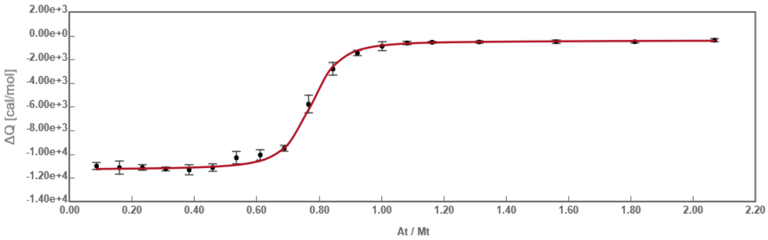

Experiment 2

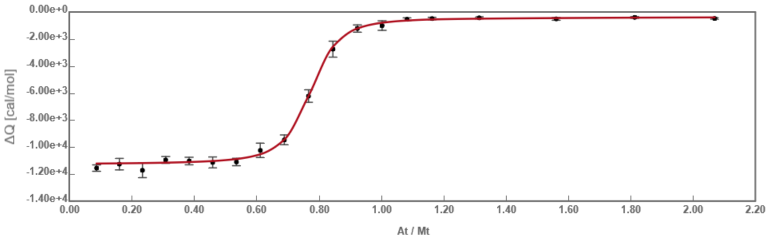

Experiment 3

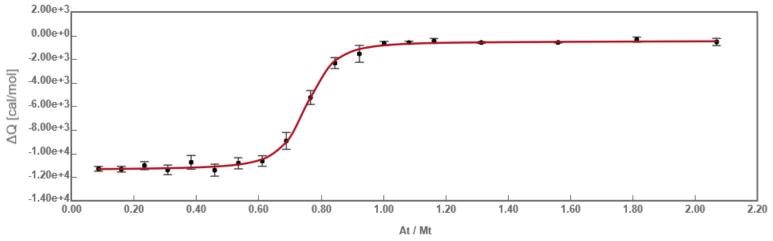

After global fitting

Experiment 1

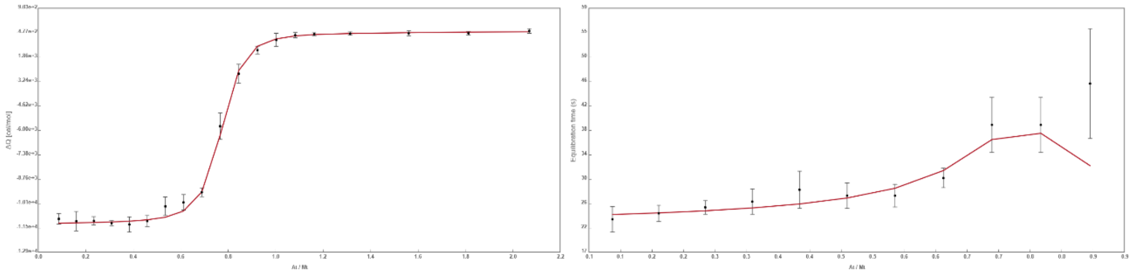

Experiment 2

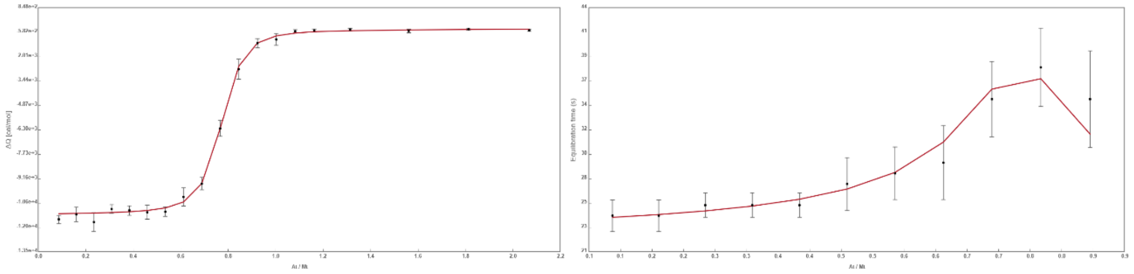

Experiment 3

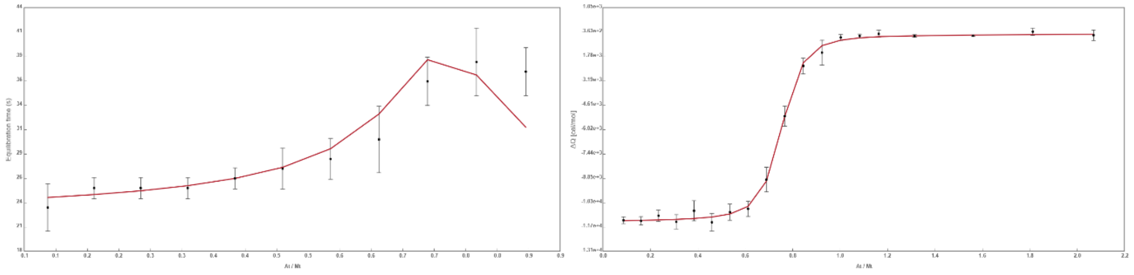

Compound 17

Before global fitting

Experiment 1

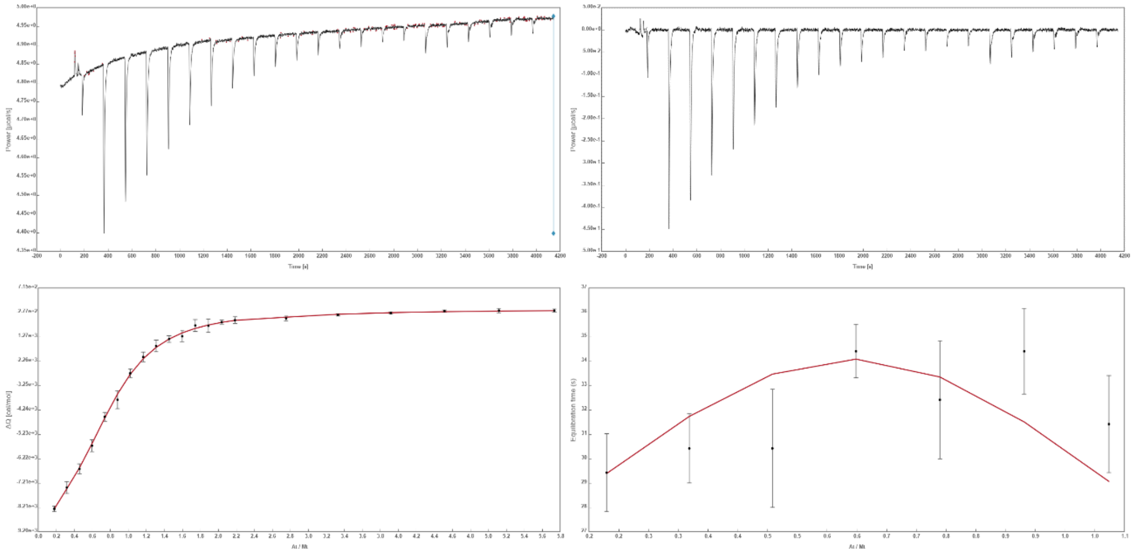

Experiment 2

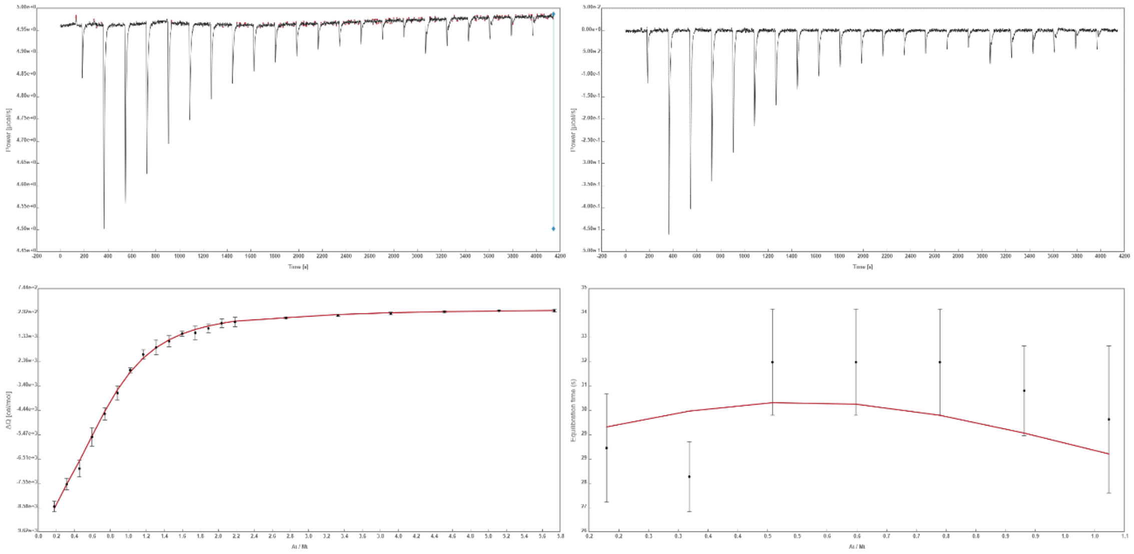

Experiment 3

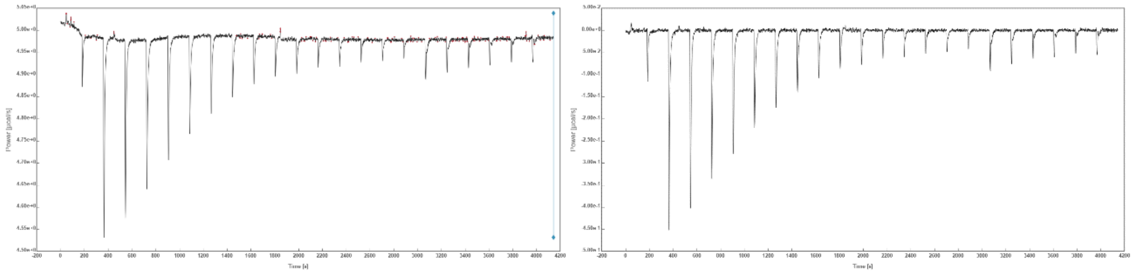

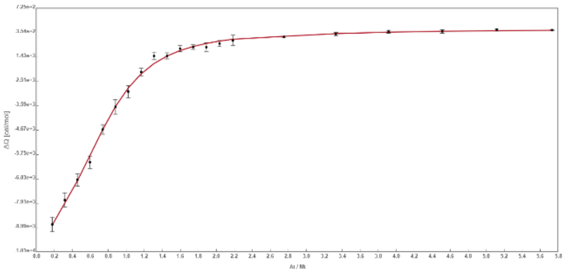

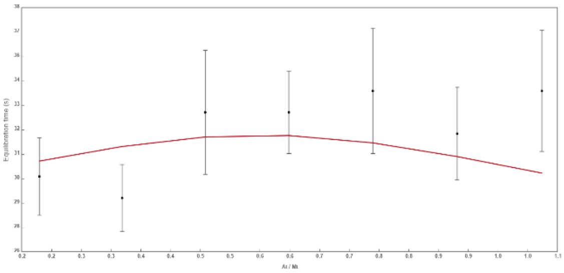

Global fitting

Experiment 1

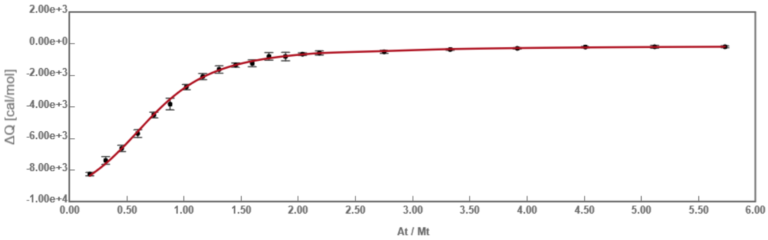

Experiment 2

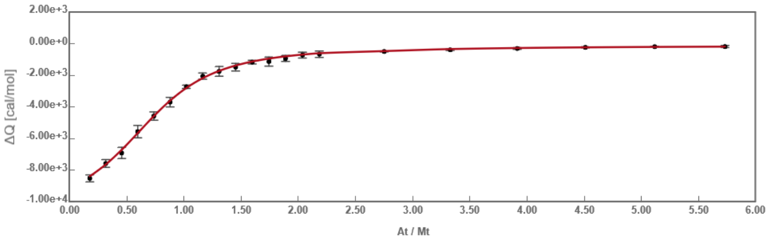

Experiment 3

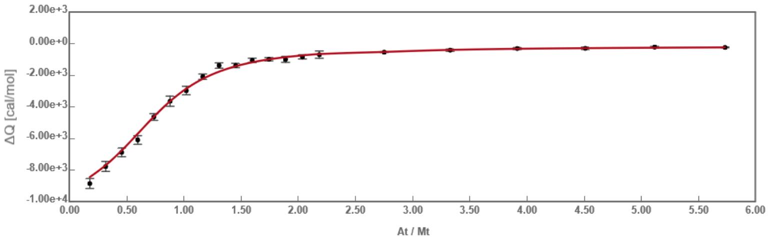

After global fitting

Experiment 1

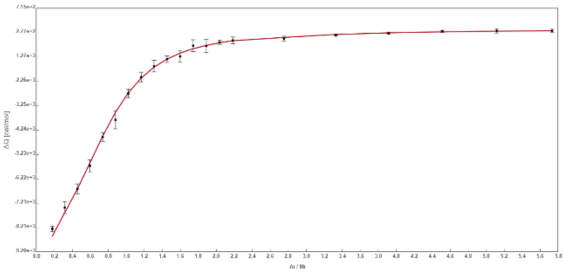

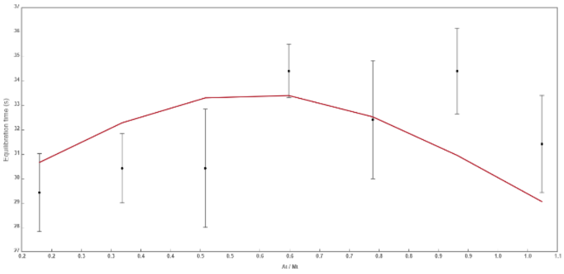

Experiment 2

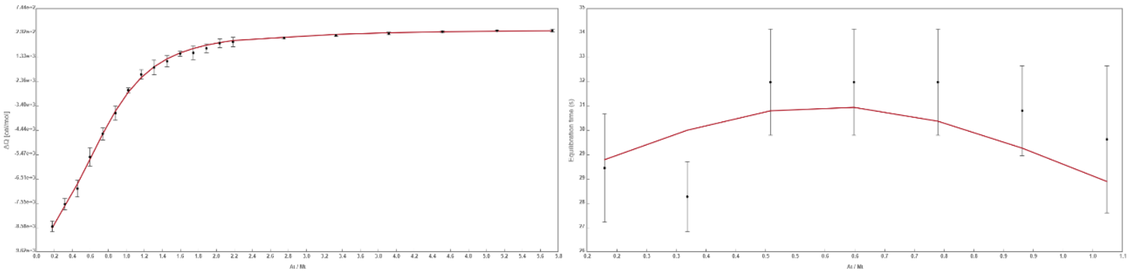

Experiment 3

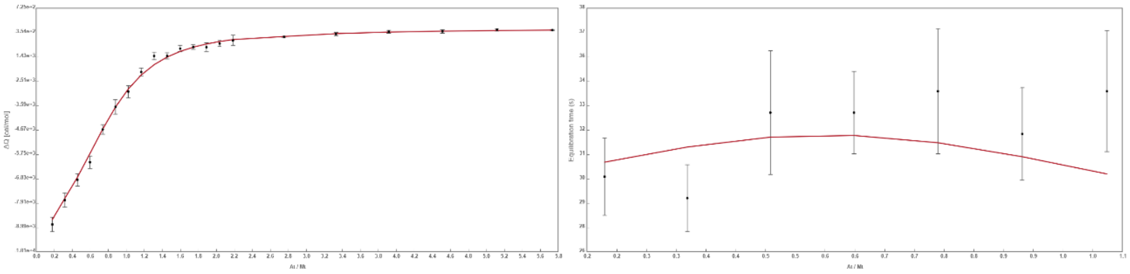

Synthesis

General Procedure (**GP1**). Chlorosulfonic acid (5.5 eq) was added to the corresponding tetrafluorobenzene (1 eq) and the mixture was heated at 150 °C. After 2 h, the solution was cooled to rt and thionyl chloride (2.2 eq) was added. The mixture was reheated for additional 3 h at 150 °C and was afterwards cooled to rt. The resulting brown solution was poured dropwise into ice water (20 mL). The obtained suspension was extracted with EtOAc (3 x 10 mL). The combined organic layer was washed with brine (20 mL), dried over $MgSO_4$, filtered and concentrated in vacuo. The crude product was directly used for the subsequent sulfonamide formation according to **GP2**.

General Procedure (**GP2**). Sulfonyl chloride (1 eq) was dissolved in THF and the mixture was cooled to -15 °C. A solution of 25% (w/v) aqueous NH_3-solution was added until the solution was adjusted to pH = 7. The mixture was stirred for 1 h at rt and water (150 mL) was added. The aqueous layer was extracted with EtOAc (3 x 100 mL), the combined organic layer was dried over MgSO4, filtered and concentrated in vacuo. The residue was purified by flash column chromatography.

*2,3-Difluorobenzenesulfonamide (**6**)*: **6** was synthesized according to **GP2** using 2,3-difluorobenzenesulfonyl chloride (1.50 g, 7.05 mmol, *Sigma Aldrich*), 25% (w/v) aqueous NH_3-solution (3.2 mL, 42.3 mmol) and THF (25 mL). The crude product was purified by flash column chromatography (cyclohexane/EtOAc, 2:1). **6** (0.962 g, 4.98 mmol, 71%) was obtained as a white-yellow solid. ^{1}H NMR (400 MHz, DMSO-d_6) δ = 7.85 (s, 2H), 7.75-7.68 (m, 1H), 7.64-7.60 (m, 1H), 7.42-7.36 (m, 1H). ^{13}C NMR (100 MHz, DMSO-d_6) δ = 149.9 (dd, *J* = 21.1, 257.5 Hz), 146.2 (dd, *J* = 11.0, 251.3 Hz), 133.6 (t, *J* = 11.0 Hz), 125.1, 123.6 (d, *J* = 2.8 Hz), 121.5 (d, *J* = 17.1 Hz). MS (ESI+) *m/z* calculated for $C_6H_9F_2N_2O_2S$ $[M+NH_4]^+$: 211.03; found: 211.10.

*2,3,5,6-Tetrafluorobenzenesulfonamide (**10**)*: According to **GP1**, the corresponding sulfonyl chloride was prepared using 2,3,5,6-tetrafluorobenzene (1.50 g, 9.99 mmol, *Alfa Aesar*), chlorosulfonic acid (3.7 mL, 55.5 mmol) and thionyl chloride (1.6 mL, 22.2 mmol).

Sulfonamide **10** was synthesized according to **GP2** using 25% (w/v) aqueous NH_3-solution (3.7 mL, 49.7 mmol) and THF (25 mL). The crude product was purified by flash column chromatography (cyclohexane/EtOAc, 4:1). **10** (1.49 g, 6.51 mmol, 65% over 2 steps) was obtained as a white-yellow solid. ^{1}H NMR (400 MHz, DMSO-d_6) δ = 8.35 (s, 2H), 8.20-8.12 (m, 1H). ^{13}C NMR (100 MHz, DMSO-d_6) δ = 146.7 (ddd, *J* = 4.3, 10.4, 14.5 Hz), 144.7 (ddd, *J* = 4.3, 10.2, 14.8 Hz), 143.6-143.4 (m), 141.5 (dt, *J* = 3.2, 15.5 Hz), 123.8 (t, *J* = 15.1 Hz), 110.2 (t, *J* = 23.6 Hz). MS (ESI+) *m/z* calculated for $C_6H_7F_4N_2O_2S$ $[M+NH_4]^+$: 228.98; found: 229.07.

*2,3,5,6-Tetrafluoro-4-methylbenzenesulfonamide (**11**)*: According to **GP1**, the corresponding sulfonyl chloride was prepared using 2,3,5,6-tetrafluoro-4-methylbenzene (0.500 g, 3.04 mmol, *abcr GmbH*), chlorosulfonic acid (1.1 mL, 16.7 mmol) and thionyl chloride (0.5 mL, 6.70 mmol). Sulfonamide **11** was synthesized according to the general procedure **GP2** using 25% (w/v) aqueous NH_3-solution (1.2 mL, 15.3 mmol) and THF (25 mL). The crude product was purified by flash column chromatography (cyclohexane/EtOAc, 4:1). **11** (0.454 g, 1.86 mmol, 61% over 2 steps) was obtained as a white-yellow solid. ^{1}H NMR (400 MHz, DMSO-d_6) δ = 8.29 (s, 2H), 2.29 (s, 3H). ^{13}C NMR (100 MHz, DMSO-d_6) δ = 146.0-145.7 (m), 143.6-143.3 (m), 141.0 (dt, *J* = 3.8, 15.5 Hz), 120.9-120.1 (m), 7.75. MS (ESI+) *m/z* calculated for $C_7H_9F_4N_2O_2S$ $[M+NH_4]^+$: 261.03; found: 261.11.

*2,3,5,6-Tetrafluoro-4-ethylbenzenesulfonamide (**12**)*: *n*-Butyllithium (1.6 M, 6.9 mL, 10.9 mmol) was added dropwise to a stirred solution of 2,3,5,6-tetrafluorobenzene (1.50 g, 9.99 mmol, *Alfa Aesar*) in THF (50 mL) at -60 °C. The mixture was stirred for 2 h at -45 °C. 1-Iodoethane (0.8 mL, 10.9 mmol) was added dropwise to the mixture. After 0.5 h the mixture was warmed to rt and poured into water (100 mL). The mixture was extracted with Et_2O (3 x 50 mL) and the combined organic layer was dried over anhydrous $MgSO_4$ and Et_2O was removed under atmospheric pressure. The crude product was purified by distillation (up to 140 °C). 2,3,5,6-tetrafluoro-4-ethylbenzene (1.15 g, 6.48 mmol, 65%) was obtained as a yellow oil. According to **GP1**, the corresponding sulfonyl chloride was prepared using 2,3,5,6-tetrafluoro-4-ethylbenzene (1.14 g, 6.42 mmol), chlorosulfonic acid (2.1 mL, 32.1 mmol) and thionyl chloride (1.0 mL, 14.1 mmol). Sulfonamide **12** was synthesized according to the general procedure **GP2** using 25% (w/v) aqueous NH_3-solution (2.0 mL, 26.6 mmol) and

THF (40 mL). The crude product was purified by flash column chromatography (cyclohexane/EtOAc, 4:1). **12** (0.824 g, 3.38 mmol, 35% over 3 steps) was obtained as a white-yellow solid. ^{1}H NMR (400 MHz, DMSO-d_6) δ = 8.34 (s, 2H), 2.75 (s, 2H), 1.18 (t, *J* = 7.6 Hz). ^{13}C NMR (100 MHz, DMSO-d_6) δ = 145.4-145.2 (m), 143.5-143.2 (m), 141.3 (dt, *J* = 3.7, 15.8 Hz), 125.5 (t, *J* = 18.8 Hz), 121.0 (t, *J* = 15.3 Hz), 16.2, 13.1. MS (ESI+) *m/z* calculated for $C_8H_{11}F_4N_2O_2S$ $[M+NH_4]^+$: 275.05; found: 275.09.

*2,3,5,6-Tetrafluoro-4-propylbenzenesulfonamide (**13**)*: *n*-Butyllithium (1.6 M, 6.9 mL, 10.9 mmol) was added dropwise to a stirred solution of 2,3,5,6-tetrafluorobenzene (1.50 g, 9.99 mmol, *Alfa Aesar*) in THF (50 mL) at -60 °C. The mixture was stirred for 2 h at -45 °C. 1-Bromopropane (1.1 mL, 10.9 mmol) was added dropwise to the mixture. After 0.5 h the mixture was warmed to rt and poured into water (100 mL). The mixture was extracted with Et_2O (3 x 50 mL) and the combined organic layer was dried over anhydrous $MgSO_4$ and Et_2O was removed under atmospheric pressure. The crude product was purified by distillation (up to 140 °C). 2,3,5,6-tetrafluoro-4-propylbenzene (1.52 g, 7.93 mmol, 79%) was obtained as a yellow oil. According to **GP1**, the corresponding sulfonyl chloride was prepared using 2,3,5,6-tetrafluorobenzene (1.52 g, 7.94 mmol), chlorosulfonic acid (2.9 mL, 43.6 mmol) and thionyl chloride (1.3 mL, 17.4 mmol). Sulfonamide **13** was synthesized according to **GP2** using 25% (w/v) aqueous NH_3-solution (3.4 mL, 45.4 mmol) and THF (60 mL). The crude product was purified by flash column chromatography (cyclohexane/EtOAc, 5:1). **13** (0.107 g, 0.394 mmol, 4% over 3 steps) was obtained as a white-yellow solid. ^{1}H NMR (400 MHz, DMSO-d_6) δ = 8.29 (s, 2H), 2.73 (t, *J* = 7.4 Hz, 2H), 1.64-1.56 (m, 2H), 0.91 (t, *J* = 7.4 Hz, 3H). ^{13}C NMR (100 MHz, DMSO-d_6) δ = 145.9-145.6 (m), 143.5-143.2 (m), 141.0 (dt, *J* = 15.7, 3.7 Hz), 124.0 (t, *J* = 19.1 Hz), 121.1 (t, *J* = 15.3 Hz), 24.3, 21.6, 13.2. MS (ESI+) *m/z* calculated for $C_9H_{13}F_4N_2O_2S$ $[M+NH_4]^+$: 289.06; found: 289.12.

Determination of purity

The purity of all ligands was determined by analytical HPLC with a Shimadzu LC-10A system (reversed-phase column: Nucleodur C18, 5 µm, 100 Å, 4.6 x 250 mm, Macherey-Nagel, Düren, Germany). All solvents were HPLC grade and in a gradient run the percentage of acetonitrile was increased 1% solvent min^{-1} at a flow rate of 1 mL min^{-1}. The detection was recorded at a wavelength of 220 nm. ^{1}H and ^{13}C NMR spectra were measured on a JEOL ECX-400 instrument. Chemical shifts are reported in ppm using residual peaks for the deuterated solvent as internal standard [11]: DMSO-d_6, 2.50 ppm (^{1}H NMR), 39.5 ppm (^{13}C NMR). The multiplicity of the signals is described with the following abbreviations: s = singlet, d = doublet, t = triplet and m = multiplet. The coupling constants *J* are given in Hz. MS spectra were measured on a Q-Trap 2000 system with an electrospray interface (ESI).

References

1. Karplus, P.A.; Diederichs, K. Linking Crystallographic Model and Data Quality. *Science* **2012**, *336*, 1030–1033.

2. Arndt, U.W.; Crowther, R.A.; Mallett, J.F.W. A computer-linked cathode-ray tube microdensitometer for X-ray crystallography. *J. Phys. E.* **1968**, *1*, 510–516.

3. Brünger, A.T. Free *R* value: a novel quantity for assessing the accuracy of crystal structures. *Nature* **1992**, *355*, 472–475.

4. Winn, M.D.; Ballard, C.C.; Cowtan, K.D.; Dodson, E.J.; Emsley, P.; Evans, P.R.; Keegan, R.M.; Krissinel, E.B.; Leslie, A.G.W.; McCoy, A.; et al. Overview of the CCP4 suite and current developments. *Acta Crystallogr., Sect. D: Biol. Crystallogr.* **2011**, *67*, 235–242.

5. Laskowski, R.A.; MacArthur, M.W.; Moss, D.S.; Thornton, J.M. PROCHECK: a program to check the stereochemical quality of protein structures. *J. Appl. Crystallogr.* **1993**, *26*, 283–291.

6. Kleywegt, G.J.; Zou, J.Y.; Kjeldgaard, M.; Jones, T.A. Around O. In *International Tables for Crystallography, Vol. F. Crystallography of Biological Macromolecules*; Rossmann, M.G., Arnold, E., Eds.; Dordrecht: Kluwer Academic Publisher, The Netherlands, 2001; pp. 353–356, 366–367.

7. Glöckner, S.; Ngo, K.; Sager, C.P.; Hüfner-Wulsdorf, T.; Heine, A.; Klebe, G. Conformational Changes in Alkyl Chains Determine the Thermodynamic and Kinetic Binding Profiles of Carbonic Anhydrase Inhibitors. *ACS Chem. Biol.* **2020**, DOI: 10.1021/acschembio.9b00895.

8. Dullweber, F.; Stubbs, M.T.; Musil, D.; Stürzebecher, J.; Klebe, G. Factorising Ligand Affinity: A Combined Thermodynamic and Crystallographic Study of Trypsin and Thrombin Inhibition. *J. Mol. Biol.* **2001**, *313*, 593–614.

9. Dumas, P.; Ennifar, E.; Bec, G.; Piñero, A.; Sabín, J.; Muñoz, E.; Rial, J. *Implementation of kinITC into AFFINImeter*; 2015;

10. Vasilief, I. QtiPlot 1.0.0-rc2 2018.

11. Gottlieb, H.E.; Kotlyar, V.; Nudelman, A. NMR Chemical Shifts of Common Laboratory Solvents ans Trace Impurities. *J. Org. Chem.* **1997**, *62*, 7512–7515.
